# siRNA screen of the human signaling proteome identifies the PtdIns(3,4,5)P_3_-mTOR signaling pathway as a primary regulator of transferrin uptake

**DOI:** 10.1186/gb-2007-8-7-r142

**Published:** 2007-07-19

**Authors:** Thierry Galvez, Mary N Teruel, Won Do Heo, Joshua T Jones, Man Lyang Kim, Jen Liou, Jason W Myers, Tobias Meyer

**Affiliations:** 1 Department of Chemical and Systems Biology and Bio-X Program, Stanford University School of Medicine, Stanford, CA 94305, USA

## Abstract

**Background:**

Iron uptake via endocytosis of iron-transferrin-transferrin receptor complexes is a rate-limiting step for cell growth, viability and proliferation in tumor cells as well as non-transformed cells such as activated lymphocytes. Signaling pathways that regulate transferrin uptake have not yet been identified.

**Results:**

We surveyed the human signaling proteome for regulators that increase or decrease transferrin uptake by screening 1,804 dicer-generated signaling small interfering RNAs using automated quantitative imaging. In addition to known transport proteins, we identified 11 signaling proteins that included a striking signature set for the phosphatidylinositol-3,4,5-trisphosphate (PtdIns(3,4,5)P3)-target of rapamycin (mTOR) signaling pathway. We show that the PI3K-mTOR signaling pathway is a positive regulator of transferrin uptake that increases the number of transferrin receptors per endocytic vesicle without affecting endocytosis or recycling rates.

**Conclusion:**

Our study identifies the PtdIns(3,4,5)P3-mTOR signaling pathway as a new regulator of iron-transferrin uptake and serves as a proof-of-concept that targeted RNA interference screens of the signaling proteome provide a powerful and unbiased approach to discover or rank signaling pathways that regulate a particular cell function.

## Abstract 2

A survey of 1,804 human dicer-generated signaling siRNAs using automated quantitative imaging identified the phosphatidylinositol-3,4,5-trisphosphate-mTOR signaling pathway as a primary regulator of iron-transferrin uptake.

## Background

**Figure 1:**
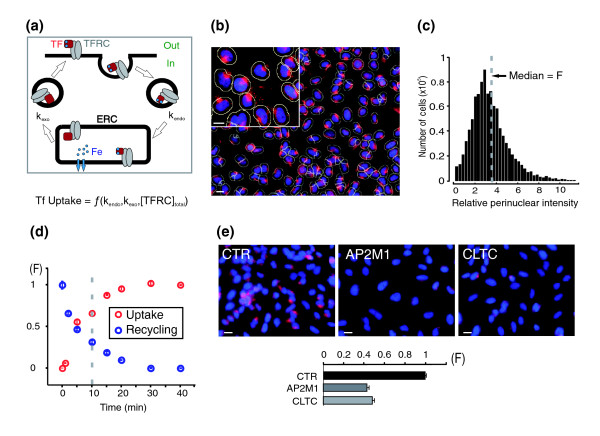
Automated image-based quantification of iron-loaded transferrin uptake in HeLa cells. **(a) **Schematic representation of the transferrin-mediated iron uptake system. Transferrin receptors (TFRC, grey ovals) cycle between the plasma membrane (PM) and the endosomal recycling compartment (ERC) using vesicular carrier (black circles). Iron (Fe^3+^, blue circles) binds to TFRC at the cell surface and is released into the ERC. At the steady-state, the quantity of internalized transferrin depends on the rate of transferrin endocytosis (k_endo_), the rate of transferrin recycling (k_exo_), and on the total number of cycling transferrin receptors. **(b) **Fluorescent transferrin uptake in mock-transfected HeLa cells (red). Hoechst-stained nuclei (blue) were used to segment the images and create the perinuclear regions (yellow) used to measure the fluorescence intensity in the 'red' channel. **(c) **Histogram of single cell fluorescence intensities (the median (F) is the transferrin uptake index used in this study). **(d) **Time-course of fluorescent transferrin uptake (red circles) and recycling (blue circles). The dashed line indicates the time point chosen for the screen. Means ± standard error of the mean (*n *= 4 replicates). **(e) **Images and quantification of fluorescent transferrin uptake in cells transfected with d-siRNAs targeting GL3 luciferase (CTR), the μ2 subunit of the AP2 adapter (AP2M1) or the clathrin heavy chain (CLTC). Means ± standard error of the mean (*n *= 3 experiments). Scale bars, 10 μm.

Iron is an essential nutrient that functions as a co-factor for enzymes that perform single electron oxidation-reduction reactions [1,2]. Intracellular iron deficiency leads to cell cycle arrest in G1 phase and apoptosis [3,4], whereas an excess of cytosolic iron causes oxidative stress and necrosis through the production of reactive oxygen species [5]. Since neither iron deficiency nor excess are tolerated by cells, iron uptake and consumption must be tightly coordinated. Nearly all extracellular iron is bound to transferrin and uptake of iron-loaded transferrin is mediated primarily by the transferrin receptor (also named TfR1 or TFRC), which is internalized by clathrin-mediated endocytosis [6]. Iron is released from transferrin in the acidic endosomal environment and reaches the cytosol via divalent metal transporter 1 [2,7]. Transferrin and its receptor recycle back to the cell surface where transferrin dissociates and is used for further cycles of iron binding and uptake (Figure 1a). There are three main determinants for transferrin and iron uptake: the rate of receptor internalization (k_endo_), the rate of recycling (k_exo_), and the total number of transferrin receptors involved in the endocytic cycle (Figure 1a). Here we performed a targeted small interfering RNA (siRNA) screen of the human signaling proteome to identify signaling molecules and pathways that regulate transferrin uptake and can thereby limit iron availability and cell growth.

## Results and discussion

### A transferrin uptake siRNA screen of the human signaling proteome

We developed a functional genomic approach to survey the human signaling proteome and identify potential signaling pathways controlling transferrin uptake. Increases or decreases in the rate of transferrin uptake were detected by monitoring the endosomal concentration of fluorescent transferrin using automated and quantitative high-throughput imaging. We applied automated image processing to measure the fluorescence intensity of perinuclear recycling endosomes in hundreds of individual cells per well of a 96-well plate (Figure 1b). The median of the measured single cell fluorescence intensity values (F) in a well was used as a measure of transferrin uptake (Figure 1c). This quantification was sufficiently sensitive to measure the time-course of transferrin uptake and recycling (Figure 1d), as well as to detect expected decreases in transferrin uptake when we knocked down elements of the endocytic machinery, μ2 subunit of the AP2 adaptor (AP2M1) or clathrin heavy chain (CLTC), using diced pools of siRNA (Figure 1e). The coefficient of variation of the measured uptake values in a 96-well plate format was less than 5% (Figure S1 in Additional data file 1).

**Figure 2:**
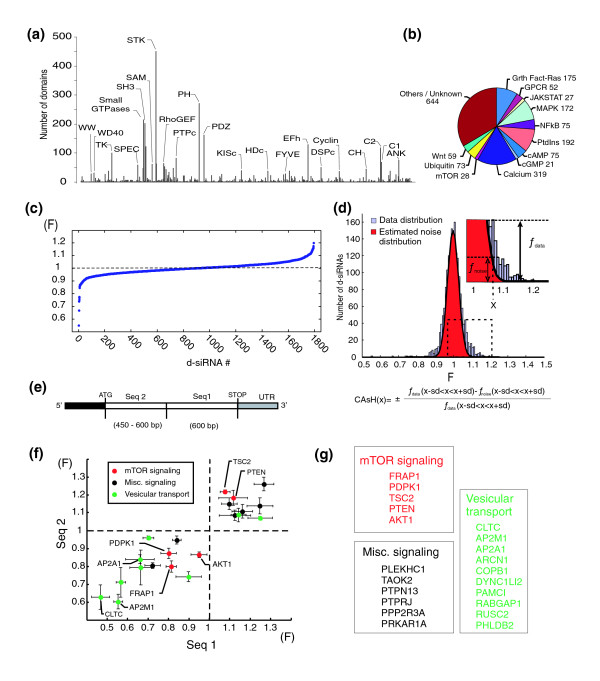
Diced-siRNA screen of the human signaling proteome identifies regulators of transferrin uptake. **(a) **Selected d-siRNAs include proteins with known signaling related domains (as named and annotated in the NCBI CDD database of 20 July 2005 [29]. Related domains were pooled in the same category bin). Transmembrane receptors and transcription factors were excluded. **(b) **Examples of signaling pathways and numbers of their components represented in the selected signaling proteome set. **(c) **Measured transferrin uptake values (F) from the human signaling proteome screen. Data sorted in ascending order. **(d) **Method to calculate hit probabilities by CAsH analysis. The histogram of the screen data (light blue) is compared to the stochastic noise distribution (red). CAsH values correspond to the estimated fraction of hits for each F score. **(e) **Regions within mRNAs selected to design the two independent d-siRNA pools. Seq1 corresponds to the region chosen to synthesize the initial batches of d-siRNAs, Seq2 to the region chosen to synthesize the second, independent batch. **(f) **Transferrin uptake regulators identified by two independent d-siRNA pools. **(g) **Genes increasing or decreasing transferrin uptake in HeLa cells.

In order to probe the signaling pathways that may regulate transferrin uptake, we generated a library of diced siRNAs (d-siRNA) that included a comprehensive set of 1,920 genes from the predicted human signaling proteome (see Additional data file 2 for the complete list of the targeted genes). Targeted gene products were selected based on their signaling domain content, for example, kinase, SH2, PH, or PDZ domains, as well as descriptive key words, such as signal transduction, endocytosis and exocytosis, as annotated by the National Center for Biotechnology Information (NCBI) Refseq and Conserved Domains (CDD) databases (Figure 2a; Additional data file 2). Components of canonical signaling pathways were included (for example, Ca^2+^, cAMP, and ERK), as well as less characterized and putative signaling proteins and pathways (Figure 2b). We also targeted a number of specific vesicular transport-associated proteins expected to affect transferrin trafficking, such as clathrin chains, clathrin adapters and coat proteins, and included them as positive controls. This signaling RNA interference (RNAi) library was synthesized using a 96-well formatted protocol to generate double-stranded RNAs that were digested *in vitro *with recombinant human RNase III Dicer protein to produce target specific d-siRNA pools [8] (see Figure S2 in Additional data file 1 for an illustration of the method). Amongst the initial 1,920 d-siRNAs, 1,804 passed our quality controls (see Materials and methods) and will be eventually considered. D-siRNAs have been shown in previous studies to provide, in nearly all tested cases, more than 50% knock-down of targeted gene products [8-11].

Fluorescent transferrin uptake was measured in duplicate for the 1,804 signaling d-siRNAs 60 hours after transfection. The average of the two normalized F scores is shown for each of the 1,804 d-siRNAs in Figure 2c. The scatter plot of the F scores obtained from duplicated d-siRNAs shows that the majority of hits that strongly deviated from the median were reproducible (Figure S3 in Additional data file 1).

An important step for identifying signaling proteins and pathways in a siRNA screen is to decide whether a particular value significantly deviates from the stochastic experimental noise. We developed a tool that we call CAsH (for Confidence Analysis of siRNA Hits) that compares each measured value to the noise distribution observed in the screen. The distribution of the variances between the duplicate measurements was used to evaluate the noise distribution, assuming that multiple measurements of the same d-siRNA, whether effective hits or not, have the same variance as repetitions of control d-siRNA (Figure 2d). Thus, the experimental noise is directly estimated from the relevant data set with no need to assay in parallel a large population of identical d-siRNAs. For each average F score from each d-siRNA, the calculated CAsH parameter then gives a probability score between -1 and 1. For instance, an effect with a CAsH score equal to -0.90 or 0.90 is expected to be observed 90% of the time as siRNA with suppressor or activator activity, respectively. Whereas the z-scores frequently used in siRNA screens are indicative of the position of a given siRNA relative to the whole data set distribution (therefore, their values depend on the hit content of the library), the CAsH scores reflect the confidence that the effect of a siRNA of interest will be observed again. Our screen had 183 d-siRNAs that resulted in an absolute value of the CAsH score ≥0.95. (see Additional data file 3 for the list of primary hits and Figure S4 in Additional data file 1 for the distribution of the CAsH scores).

To reduce the number of false positive caused by systematic sources of noise occurring during the screening process, the primary hits were assayed again (using a higher concentration of d-siRNAs; 100 nM versus 20 nM). There were 154 genes (84%) that presented similar or stronger effects than observed in the primary screen. Amongst these genes, 91 were selected (based on consistency between replicates, quality of the diced siRNA pools (aspect on gel) and the length of their coding sequence (>900 base-pairs (bp) to allow the synthesis of a second independent batch of d-siRNA; see below)) and further assayed with a new batch of d-siRNA using the same sequence as the one used to perform the initial screen: 71 genes (approximately 80%) showed identical results between the two batches (Additional data file 3). Furthermore, in order to determine whether a particular hit is caused by on- or off-target effects, we performed another round of validation by testing a second set of d-siRNA pools that targeted a different region of the mRNA coding sequence (Figure 2e, and see Additional data file 6 for the nucleotide sequences of the primers used). The probability that off-target effects were observed for such matching pairs of d-siRNAs is predicted to decrease with the square of the off-target rate for a single d-siRNA (off-target rate for paired hits is estimated to be <2.5 × 10^-3^; see Materials and methods). Using both sets of d-siRNAs (at a concentration of 20 nM), we identified 21 pairs among the 71 selected genes that showed a significant effect for both the first and second d-siRNA pools (Figure 2f,g, and see Additional data file 4 for the list of the 21 high confidence hits). This validation rate of 21 out of 71 is likely not a result of off-target effects since we estimate that the upper limit for off-target effects is 5% (see Materials and methods). Systematic variations occurring during the d-siRNA synthesis process or intrinsic differences of the two template regions in producing efficient d-siRNAs, might explain why only one out of two d-siRNA pool is effective.

### Identification of the mTOR pathway as primary signaling module controlling transferrin uptake

**Figure 3:**
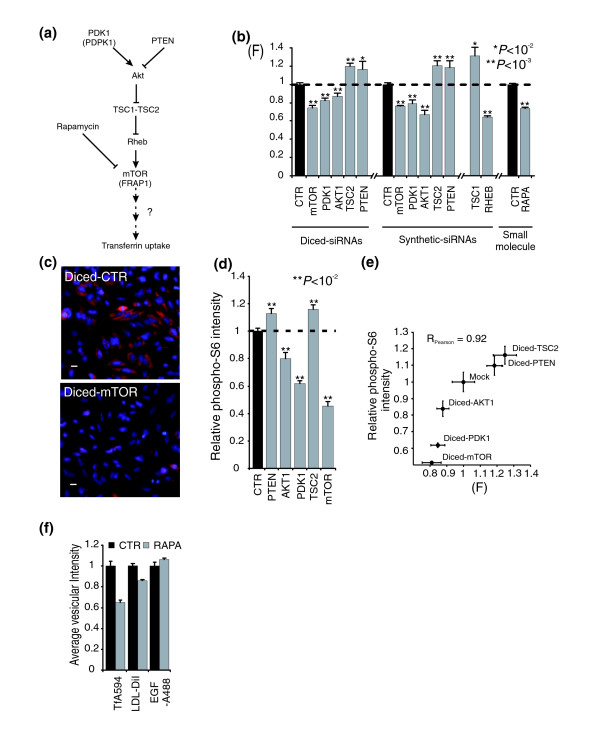
The PtdIns(3,4,5)P3-mTOR signaling pathway controls transferrin uptake. **(a) **Schematic representation of the PtdIns(3,4,5)P3-mTOR signaling pathway. **(b) **Quantification of the effects of different siRNAs targeting the PtdIns(3,4,5)P3-mTOR pathway and rapamycin (RAPA) on transferrin uptake. Means ± standard error of the mean (SEM; for d-siRNAs, *n *= 6; for synthetic siRNAs, *n *= 4; for rapamycin, *n *= 17). **(c-d) **Regulation of S6 protein phosphorylation by the PtdIns(3,4,5)P3-mTOR targeting d-siRNAs. Immunofluorescent images compared the effect of mTOR and GL3 luciferase (CTR) knock-downs on phosphorylated S6 protein staining (red). Nuclei are stained with Hoechst (blue). Scale bars, 10 μm. Quantification of the staining-associated average fluorescence intensity (d). Means ± SEM (*n *= 6). **(e) **Correlation between the effect of d-siRNAs on phosphorylation of S6 protein and transferrin uptake. Means ± standard deviation (*n *= 2 experiments). **(f) **Inhibition of mTOR signaling by rapamycin (20 nM, 24 h) preferentially affects transferrin uptake when compared to DiI-labeled LDL or Alexa Fluor 488-labeled EGF. Average vesicular fluorescence intensities were measured. Mean ± SEM (*n *= 6 replicates).

This set of 21 high confidence regulators of transferrin uptake contained a striking fraction of proteins from the phosphatidylinositol-3,4,5-trisphosphate (PtdIns(3,4,5)P3)-target of rapamycin (mTOR) signaling pathway [12] (Figure 3a). Two of these proteins, TSC2 and PTEN, are known mTOR suppressors whose knock-down enhances transferrin uptake. Three others (PDK1, alias PDPK1, AKT1, and mTOR, alias FRAP1) are known activators whose knock-down reduces transferrin uptake (Figure 2f). We call such a match of the players, as well as of the direction of the effects, a signature of a particular signaling pathway. In addition to signature proteins of the PtdIns(3,4,5)P3-mTOR pathway, the other hits could be grouped into a set of miscellaneous putative signaling proteins that included three protein phosphatases, a PKA regulatory subunit, a cytoskeletal associated protein, PLEKHC1 [13], as well as TAOK2 [14], which has been proposed to be involved in p38 mitogen-activated protein kinase signaling. A third identified group included components and regulators of the vesicular trafficking and endocytosis machinery (Figure 2g, Additional data file 4).

We performed additional experiments to verify that the identified d-siRNAs do act on the PtdIns(3,4,5)P3-mTOR pathway and to investigate whether mTOR signaling preferentially regulates uptake of transferrin. When we compared the effect of d-siRNA pools and synthetic siRNAs, we found that knocking-down the identified activators of this pathway (mTOR, PDK1, AKT1) led in both cases to a reduction of transferrin uptake, whereas knocking-down inhibitors (TSC2, PTEN) increased uptake (Figure 3b, Figure S5a in Additional data file 1). We also tested for the effects of two other players in the mTOR pathway, tuberous sclerosis 1 (TSC1), which was initially not included in our d-siRNA library, and the GTPase RHEB. TSC1 synthetic siRNA increased transferrin uptake as did its partner TSC2, whereas RHEB synthetic siRNA had a strong inhibitory effect on transferrin uptake (Figure 3b). We further confirmed the involvement of the mTOR signaling pathway by treatment with rapamycin (20 nM for 24 hours), a small molecule inhibitor of mTOR [12], which reduced transferrin uptake to a similar extent as mTOR knock-down (Figure 3b).

We then verified whether the identified d-siRNAs targeting the PtdIns(3,4,5)P3-mTOR module interfere with mTOR signaling by using phosphorylation of the ribosomal protein S6 (on Ser235/236) as a cell-based readout for mTOR activity [15,16] (Figure 3c,d). We found marked effects on S6 protein phosphorylation that closely corresponded to the effects on transferrin uptake (Figure 3e). This supports the notion that the identified genes are part of the same signaling module that includes PtdIns(3,4,5)P3 and mTOR signaling. Taken together, these results further argue that transferrin uptake is under the control of the PtdIns(3,4,5)P3-mTOR signaling pathway.

The specificity of mTOR signaling for regulating transferrin uptake was investigated by comparing the effect of rapamycin on uptake of transferrin, low density lipoprotein (LDL) and epidermal growth factor (EGF) using fluorescently labeled ligands (Figure 3f). Consistent with a preferential role of mTOR signaling for transferrin uptake, the effect of mTOR inhibition was significantly stronger for transferrin uptake compared to LDL or EGF uptake.

### The PI3K-mTOR pathway controls iron uptake by regulating the number of transferrin receptors per endocytic vesicle

**Figure 4:**
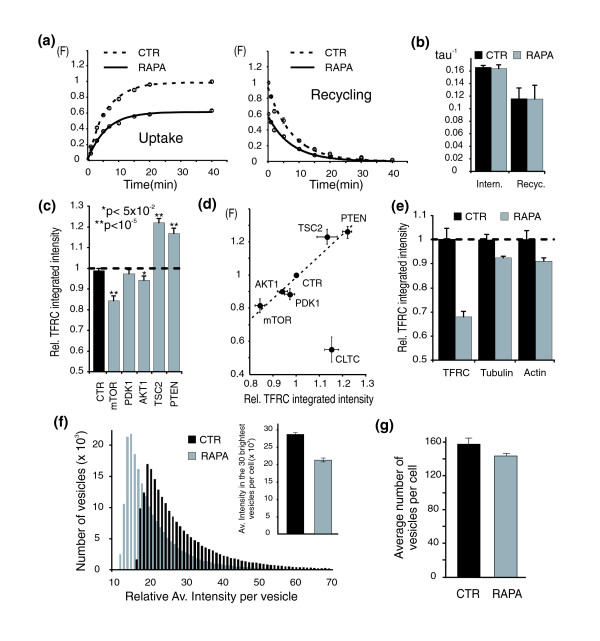
mTOR controls transferrin uptake by regulation of the number of transferrin receptors per vesicle and not endocytosis or recycling rates. **(a) **Effect of rapamycin on the time-course of fluorescent transferrin endocytosis (left) and recycling (right). Means ± standard error of the mean (SEM; *n *= 4 replicates). **(b) **Lack of effect of rapamycin on the rate constants for endocytosis and recycling. The rate constants, tau^-1^, were calculated from a single exponential fit of the time-course data. **(c) **Effect of the PtdIns(3,4,5)P3-mTOR-targeting d-siRNAs on the number of transferrin receptors (TFRC) per cell (measured by immunofluorescence on permeabilized cells). Means ± SEM (*n *= 4). **(d) **Correlation between transferrin uptake (F, vertical axis) and transferrin receptor numbers (immunofluorescence measurements on permeabilized cells, horizontal axis) in HeLa cells in which PtdIns(3,4,5)P3-mTOR regulators were silenced. Means ± SEM (*n *= 4). **(e) **Selective effect of rapamycin on transferrin receptor staining (TFRC) compared to tubulin and actin staining. Means ± SEM (*n *= 6 replicates). **(f) **Rapamycin preferentially reduces the number of transferrin molecules per vesicles. Histogram of the relative transferrin fluorescence intensity per vesicle for control (CTR) and rapamycin (RAPA)-treated cells. Means ± SEM (*n *= 6 replicates). Inset shows the average intensity measured for the 30 brightest vesicles per cell. **(g) **Rapamycin has only a minor effect on the number of endocytic vesicles. Bar diagram shows means ± SEM (*n *= 6 replicates).

Transferrin uptake activity is dependent on endocytic and recycling rates (Figure 1a), and mTOR has been reported to regulate bulk flow as well as specific forms of receptor-mediated endocytosis [17]. We therefore investigated whether part or all of the rapamycin effect on transferrin uptake could be explained by mTOR-mediated regulation of the endocytosis or recycling rates. A rapamycin-induced reduction in either one of the rates would reduce the measured transferrin uptake. While rapamycin treatment reduced the steady state concentration of internalized endosomal transferrin (Figure 4a), neither the time constant for endocytosis nor recycling were significantly altered by rapamycin (Figure 4b).

The lack of effect of rapamycin on the kinetics of endocytosis and recycling suggested that most of the effect of the mTOR pathway on transferrin uptake must result from the regulation of the total concentration of transferrin receptor (Figure 1a). Earlier studies have shown that mTOR can regulate the concentration of specific proteins and cell mass by enhancing translation via the translation regulators S6 kinase 1 and eIF4E-binding proteins [18,19], as well as by regulating protein degradation [20,21]. We tested whether the concentration of the transferrin receptor is affected by using an immunofluorescence-based assay in permeabilized cells, using transferrin receptor-specific antibodies. Targeting mTOR with d-siRNAs caused a 20% reduction of the integrated transferrin receptor fluorescence. Targeting AKT1 and PDK1 had smaller inhibitory effects, whereas targeting TSC2 or PTEN led to an increase in the integrated transferrin receptor fluorescence intensity (Figure 4c, and Figure S5b in Additional data file 1). The amplitude of the effects of different d-siRNAs on transferrin uptake were correlated with the number of transferrin receptors per cell, suggesting that increases and decreases in mTOR signaling are translated into higher and lower numbers of transferrin receptors resulting in increased or decreased transferrin uptake (Figure 4d). As a control, knock-down of clathrin heavy chain d-siRNAs (CLTC), which suppresses transferrin uptake by reducing endocytosis rates, did not show the same concomitant decrease in transferrin receptor concentration (Figure 4d). We also confirmed that rapamycin treatment (20 nM for 24 hours) had a similar effect on the integrated transferrin receptor fluorescence compared to the effect of mTOR d-siRNAs (Figure 4e). As a control, we also found that the number of transferrin receptors is much more strongly affected by mTOR inhibition compared to two other abundant reference proteins, actin and tubulin (Figure 4e). This also suggested that cell mass was only weakly affected for the conditions used in our experiments.

The reduction in transferrin uptake and total transferrin receptor number likely reflects a lower number of transferrin and transferrin receptors per endocytic carrier but could also reflect a lower number of transferrin-containing endocytic carriers. We therefore measured the relative number of transferrin molecules per vesicle by using a texture algorithm that integrated the total intensity of each vesicle over a local background. As shown in the histogram of the fluorescence intensity of individual endocytic vesicles (Figure 4f), rapamycin treatment induced a marked decrease in the transferrin staining per vesicle but only a relatively small decrease in the number of endocytic vesicles (Figure 4g). Taken together, this indicates that the PtdIns(3,4,5)P3-mTOR pathway primarily regulates the number of transferrin receptor molecules per endocytic vesicle rather than recycling rates or endosome number and that this regulation is preferential for transferrin over LDL and EGF uptake.

## Conclusion

Our study identifies the PtdIns(3,4,5)P3-mTOR signaling pathway as an important regulator of transferrin uptake, adding a new function to this important regulatory system for cell growth. The mTOR pathway is known to integrate inputs from growth factor receptors, amino acid availability, AMP/ATP ratio and 'stress' (hypoxia, DNA damage) and to respond by adjusting the rate of cell growth and proliferation [12]. Since excess iron is toxic and since iron uptake is also rate-limiting for cell growth, cells may use PtdIns(3,4,5)P3 -mTOR regulation of transferrin uptake to reduce the toxic effect of iron accumulation as well as to promote cell growth and proliferation. Considering the critical dependence of oxygen-based metabolism on intracellular iron concentration, these results may also explain the reported correlation of mTOR activity with mitochondrial oxygen consumption [22]. The question of how mTOR regulates the number of transferrin receptors remains open. mTOR-dependent stabilization of Hypoxia-inducible factor-1 (HIF-1) or c-myc [21,23], both well characterized transcriptional activators of the transferrin receptor gene [24-28], could account for the observed effect of mTOR activity on transferrin receptor level but other mechanisms based on translation or protein degradation are also plausible.

Our study provides a proof-of-concept that small human siRNA libraries focused on the signaling proteome can serve as powerful and unbiased genomic survey tools to discover or rank signaling pathways that regulate a particular cell function. Here we show that the key role of an important signaling pathway can be demonstrated from what we call a siRNA signature that consists of multiple suppressor and activator proteins in that pathway.

## Materials and methods

### Generation of diced-siRNA pools

The protocol used to synthesize d-siRNA [8] has been scaled-up to synthesize the 1,920 d-siRNAs library as previously reported [11]. Specific primers for each selected gene (Additional data file 5) were automatically designed and were used to amplify from a cDNA library an approximately 600 bp PCR fragment of the 3' region of the coding sequence. A second amplification was performed with a set of nested primers bearing a T7 promoter sequence on their 5' extension. Nested PCR products with no products or products of unexpected size (116 genes among the 1,920) were eliminated from the analysis of the screen. Nested PCR products were transcribed *in vitro *(T7 MEGA script kit, Ambion; Austin, TX, USA) and the resulting double-stranded RNAs were annealed and processed with 30 units/reaction of human recombinant Dicer (Invitrogen; Carlsbad, CA, USA) for 15 h at 37°C. The 21-mer d-siRNAs were separated from incompletely digested fragments using a succession of isopropanol precipitations and filtration on glass fiber plates (Nunc; Rochester, NY, USA). Synthetic siRNAs were from Dharmacon (Lafayette, CO, USA).

### Transfection and transferrin internalization assays

HeLa cells were plated at a density of 1,800 cells per well in 96-well plates in DMEM media supplemented with 10% fetal bovine serum and antibiotics. The d-siRNAs (approximately 20 ng/well) in a final volume of 50 μl were transfected the following day using Gene Silencer (Genlantis; San Diego, CA, USA) according to the manufacturer's protocol. Two and a half days after transfection, cells were washed twice with warmed DMEM/HEPES and incubated with 5 μg/ml Alexa Fluor^® ^594-labelled holotransferrin (Invitrogen) for 10 minutes at 37°C with 5% CO_2_. Surface-bound transferrin was stripped off with a 1 minute incubation in NaCl (500 mM)/acetic acid (200 mM). Following pH neutralization with phosphate-buffered saline, cells were fixed with 4% formaldehyde and nuclei stained with Hoechst.

Time-courses of transferrin uptake (Figures 1d and 4a) were determined as indicated above but transferrin accumulation was stopped at the indicated time. For recycling experiments, cells were first loaded with fluorescent transferrin (5 μg/ml) for 30 minutes at 37°C and then washed and incubated with non-fluorescent transferrin (50 μg/ml) for the indicated times. The rate constants, tau, were calculated from single exponential fits of the time-course data.

### LDL and EGF uptake assays

HeLa cells were incubated with DiI-LDL (1 μg/ml; Invitrogen) or biotinylated EGF in complex with Alexa Fluor^® ^488 streptavidin (200 ng/ml of complex is equivalent to 15 ng/ml of EGF; Invitrogen) for 30 minutes before surface stripping and fixation.

### Immunofluorescence staining

Formaldehyde fixed HeLa cells were permeabilized with 0.1% Triton X100, blocked with 10% goat serum and 2% bovine serum albumin and stained with anti-TFRC antibody (5 μg/ml; M-A712, BD Pharmingen; San Diego, CA, USA), anti-actin antibody (1:200; MAB1501, Chemicon International; Temecula, CA, USA), anti-β-tubulin-Cy3 antibody (1:200; Sigma; St. Louis, MO, USA) or anti-phosphorylated (S235/236) ribosomal S6 protein (1:200; Cell Signaling Technology; Danvers, MA, USA) associated with appropriate secondary antibodies (Invitrogen).

### Automated image acquisition and processing

Images were acquired on the ImageXpress 5000A automated epifluorescence microscope (Molecular Devices; Sunnyvale, CA, USA) using an ELWD 20×/0.45 Plan Fluor objective and a 1,280 × 1,024 pixels cooled CCD camera with 12-bit readout. Thirteen pairs of images (Hoechst and Alexa Fluor^® ^594 channels) were acquired per well. Image analysis was performed using the ImageXpress database and software. Images were corrected for non-uniform illumination and segmented using the nucleus-associated Hoechst fluorescence. Nuclear regions were then symmetrically expanded (+5 μm) in order to include the perinuclear endosomal recycling compartment. For each detected cell, the background-subtracted average intensities of the perinuclear regions were measured in the Alexa Fluor^® ^594 channel. This method is independent of the size of the cells but the size of nuclei might have influenced the scoring but only slightly since average intensities were considered. The scores of d-siRNAs strongly affecting cell viability did not affect the final scoring since 'toxic' d-siRNAs were generally outliers (for example, KIF11/Eg5) or did not score at all because the scores were calculated on few living cells, which were not probably transfected. COPB1 and ARCN1 affected cell viability but were considered as hits because transferrin internalization was greatly reduced even in the remaining cells.

For immunostaining, intensities integrated over the entire cell area were used to estimate the total amount of TFRC, as well as the total amount of actin or tubulin per cell. These values were extracted automatically in two steps: first, nuclear regions were expanded (+20 μm) in order to include the entire cells; and second, expanded regions were shrunk to fit the cell perimeters (using the antibody-associated intensities to threshold the images). The resulting cell areas were used to calculate single cell integrated intensities. To compare transferrin, LDL and EGF uptake, a texture detection algorithm implemented in the ImageXpress 1.0 software suite was used to identify fluorescent punctuate structures and to estimate the vesicular density per cell, as well as the average vesicle intensity. Single cell, average phospho-S6 immunofluorescence intensity was measured in a 3 μm ring around cell nuclei.

### Analysis of the relative number of transferrin molecules per vesicle

For the results presented in Figure 4f,g, a Gaussian filter adjusted for an average vesicle diameter of 2.2 pixels was used to subtract a local background intensity of each transferrin containing vesicle. The average of five pixel intensity values was used as a measure of the intensity of transferrin-loaded vesicles. The 30 brightest vesicles per cell were used for analysis. Rapamycin treatment reduced the average intensity of these 30 brightest vesicles by 25%. This was used to adjust the relative intensity threshold used for counting the number of vesicles in the control case as well as for the rapamycin treated cells.

### Estimation of the number of hits in the primary screen: CAsH analysis

The experimental noise distribution was estimated using the distribution of the deviations of the duplicated F values. The data distribution *f*_data_(x) was derived from the histogram of the experimental F values averaged with a low-pass filter (width = 2 × standard deviations of the noise). The noise distribution *f*_noise_(x) was subtracted from the data distribution *f*_data_(x) to create the hit distribution *f*_hit_(x). CAsH was defined as ±*f*_hit_(x)/*f*_data_(x) to give an estimate of the expected fraction of hits for each F value; a sign was added to indicate that a hit is a suppressor (-) or an enhancer (+) (if 0 ≤ F < 1 ≥ CAsH ≤ 0; if 1 ≤ F ≥ CAsH > 0).

### Statistical analysis

*P *values were based on two-tailed homoscedatic Student's *t*-distributions and reflect a comparison of control versus indicated siRNA, or control versus rapamycin treatment. The estimate for the off-target probability was based on the 10% hit rate of the initial screen, approximately 183/1,804. Hits occurred approximately 5% of the time in a given direction (increase versus decrease). Assuming that all the hits are off-target effects (worst case scenario), the upper boundary for off-target effects from the screen becomes 5% and the probability for two independent siRNAs to both be off-target and go in the same direction is therefore expected to be less than 0.25%.

## Additional data files

The following additional data is available with the online version of this manuscript. Additional data file 1 contains Figure S1 to S5: Figure S1 illustrates the observed well-to-well variability in the transferrin uptake assay. HeLa cells were transfected with CTR d-siRNA (targeting yellow fluorescent protein, eYFP) or d-siRNA targeting the AP2M1 gene. Twelve wells within the same row of a 96-well plate were transfected with the same pool of d-siRNAs to measure the well-to-well variability of the intensity measurements. Means ± standard deviation of two experiments are represented. Figure S2 summarizes the strategy used to generate the diced-siRNA library. Specific primers for the selected genes were designed to amplify approximately 600 bp PCR fragments from a cDNA library. A second amplification was performed with a set of nested primers bearing a T7 promoter sequence extension. Nested PCR products were *in vitro *transcribed, resulting double-stranded RNAs were diced with recombinant enzyme Dicer and the 21 mers d-siRNAs were finally purified from incomplete digests. Figure S3 shows the relationship between duplicated values of F for the 1,920 screened d-siRNAs (red). The data are compared to a simulated, two-dimensional Normal distribution of the stochastic noise (blue). The estimated standard deviation of the noise distribution is based on the measured deviations between duplicated measurements for the same d-siRNA. Figure S4 shows the distribution of the CAsH scores from the primary screen. The population of genes with CAsH >0.95 are indicated in red. Figure S5 is an example of the images used for quantitative analysis. **(a) **Images of HeLa cells illustrating the effect of d-siRNAs targeting mTOR (diced-mTOR) or GL3 luciferase (CTR) on fluorescent transferrin (red) uptake, as well as the effect of 24 h treatment with rapamycin (RAPA) compared to DMSO (CTR). Scale bars: 20 μm. Insets show a magnified view of the indicated image subset. **(b) **Immunofluorescent staining (green) of transferrin receptor performed on permeabilized cells in order to estimate the total number of transferrin receptors. Examples of cells transfected with d-siRNAs targeting mTOR, TSC2 or GL3 luciferase (CTR). Scale bars, 10 μm. Insets show a magnified view of the indicated image subset. Additional data file 2 is the list of the genes targeted by the human d-siRNA signaling library. Additional data file 3 is the list of hits from the primary screen. Additional data file 4 is the list of the 21 identified 'high confidence' genes that increase or decrease transferrin uptake in HeLa cells. Additional data file 5 contains the nucleotide sequences of the designed external (outer) and nested primers for creating Dicer siRNAs targeting 1,920 signaling proteins. Additional data file 6 contains the nucleotide sequences of the designed primers for the two independent sequences used to target the 21 high confidence hits as well as the sequences of the expected amplicons.

## Supplementary Material

Additional data file 1 Figure S1 illustrates the observed well-to-well variability in the transferrin uptake assay. HeLa cells were transfected with CTR d-siRNA (targeting yellow fluorescent protein, eYFP) or d-siRNA targeting the AP2M1 gene. Twelve wells within the same row of a 96-well plate were transfected with the same pool of d-siRNAs to measure the well-to-well variability of the intensity measurements. Means ± standard deviation of two experiments are represented. Figure S2 summarizes the strategy used to generate the diced-siRNA library. Specific primers for the selected genes were designed to amplify approximately 600 bp PCR fragments from a cDNA library. A second amplification was performed with a set of nested primers bearing a T7 promoter sequence extension. Nested PCR products were *in vitro *transcribed, resulting double-stranded RNAs were diced with recombinant enzyme Dicer and the 21 mers d-siRNAs were finally purified from incomplete digests. Figure S3 shows the relationship between duplicated values of F for the 1,920 screened d-siRNAs (red). The data are compared to a simulated, two-dimensional Normal distribution of the stochastic noise (blue). The estimated standard deviation of the noise distribution is based on the measured deviations between duplicated measurements for the same d-siRNA. Figure S4 shows the distribution of the CAsH scores from the primary screen. The population of genes with CAsH >0.95 are indicated in red. Figure S5 is an example of the images used for quantitative analysis. **(a) **Images of HeLa cells illustrating the effect of d-siRNAs targeting mTOR (diced-mTOR) or GL3 luciferase (CTR) on fluorescent transferrin (red) uptake, as well as the effect of 24 h treatment with rapamycin (RAPA) compared to DMSO (CTR). Scale bars: 20 μm. Insets show a magnified view of the indicated image subset. **(b) **Immunofluorescent staining (green) of transferrin receptor performed on permeabilized cells in order to estimate the total number of transferrin receptors. Examples of cells transfected with d-siRNAs targeting mTOR, TSC2 or GL3 luciferase (CTR). Scale bars, 10 μm. Insets show a magnified view of the indicated image subset. Click here for file

Additional data file 2 Genes targeted by the human d-siRNA signaling library. Click here for file

Additional data file 3 Hits from the primary screen. Click here for file

Additional data file 4 The 21 identified 'high confidence' genes that increase or decrease transferrin uptake in HeLa cells. Click here for file

Additional data file 5 Nucleotide sequences of the designed external (outer) and nested primers for creating Dicer siRNAs targeting 1,920 signaling proteins. Click here for file

Additional data file 6 Nucleotide sequences of the designed primers for the two independent sequences used to target the 21 high confidence hits as well as the sequences of the expected amplicons. Click here for file
