# Supplementary figures and images for: siRNA screen of the human signaling proteome identifies the PtdIns(3,4,5)P3-mTOR signaling pathway as a primary regulator of transferrin uptake
Source: Genome Biol. 2007 Jul 19;8(7):R142. doi: 10.1186/gb-2007-8-7-r142 (PMC2323231; doi:10.1186/gb-2007-8-7-r142)

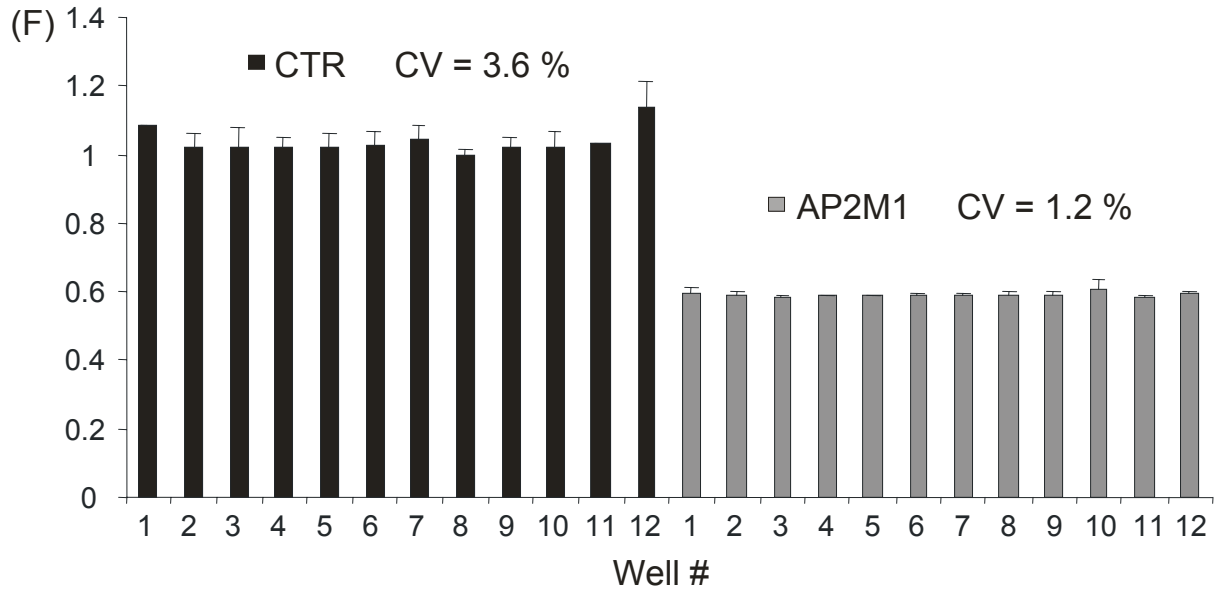

Figure S1

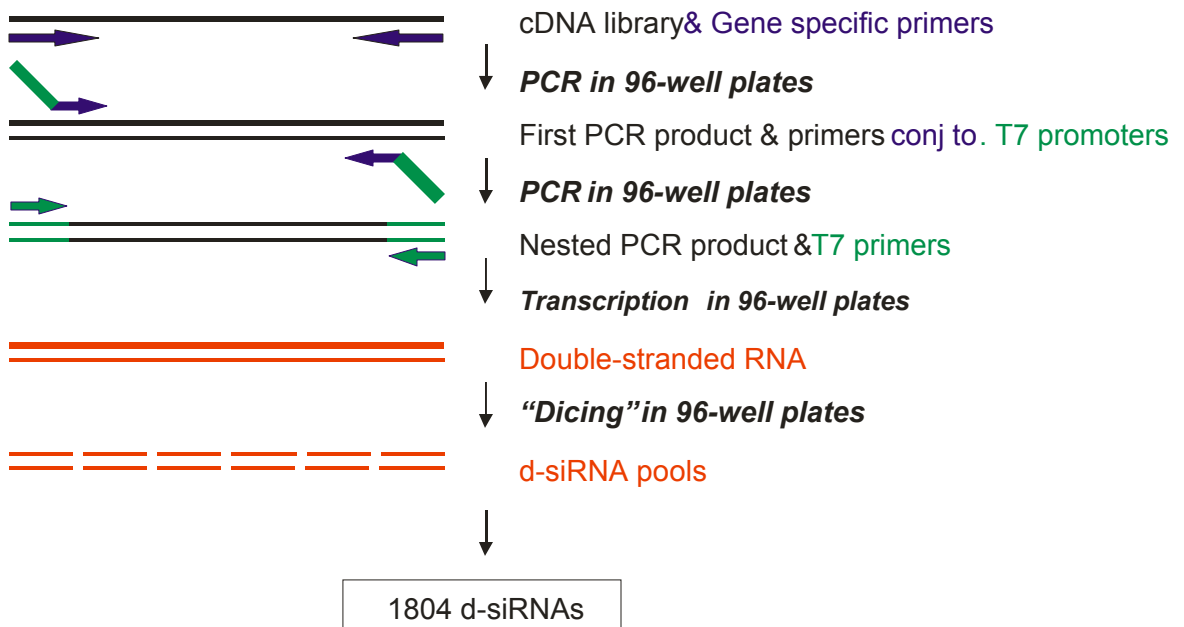

Figure S2

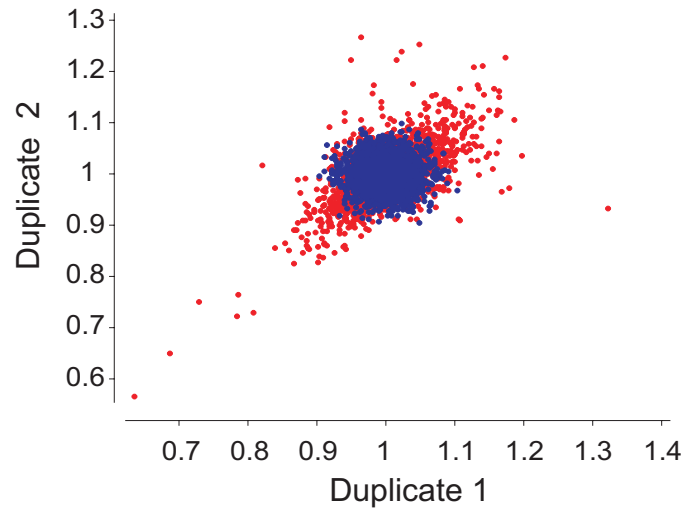

Figure S3

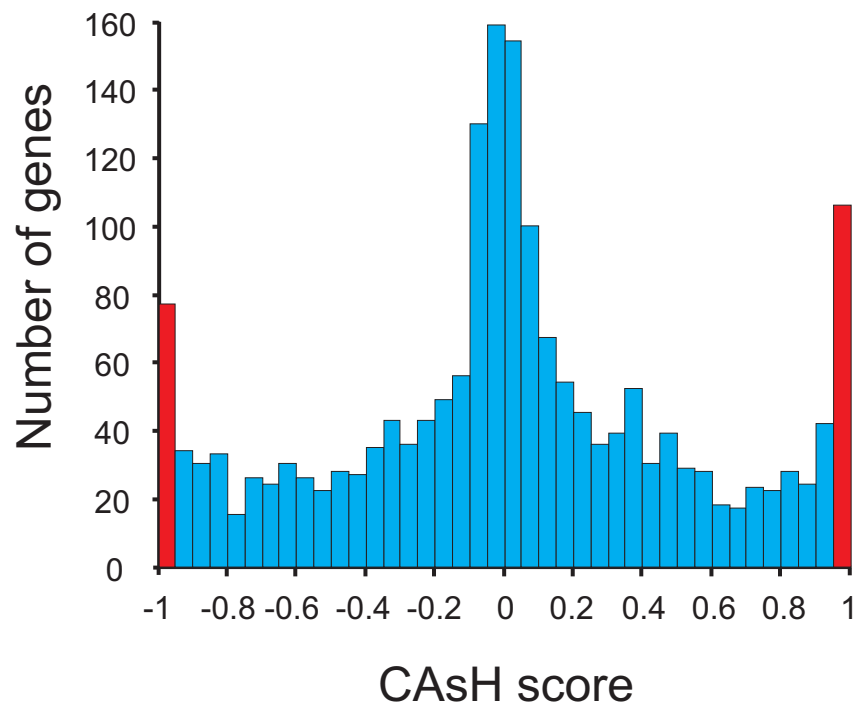

Figure S4

A

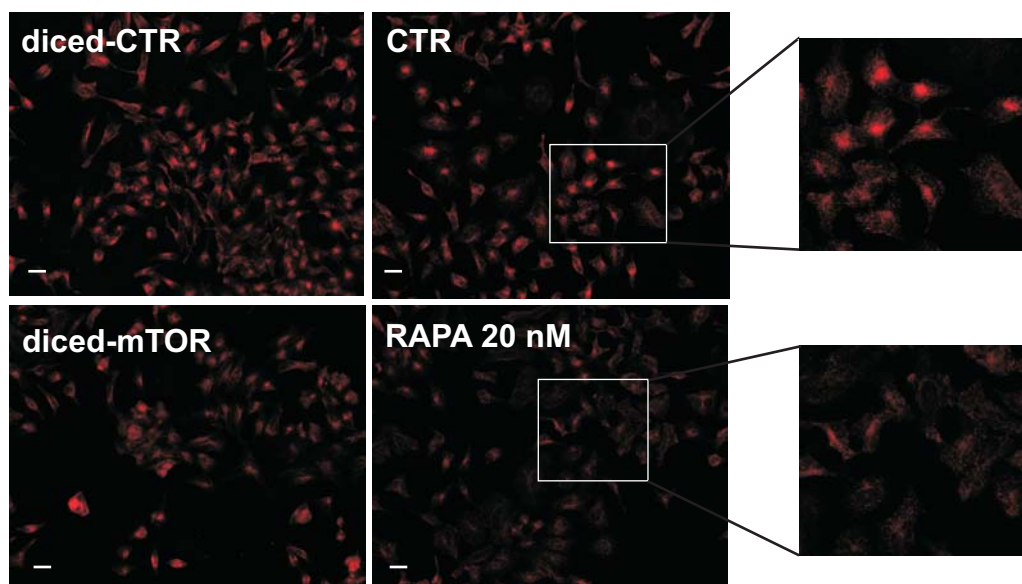

B

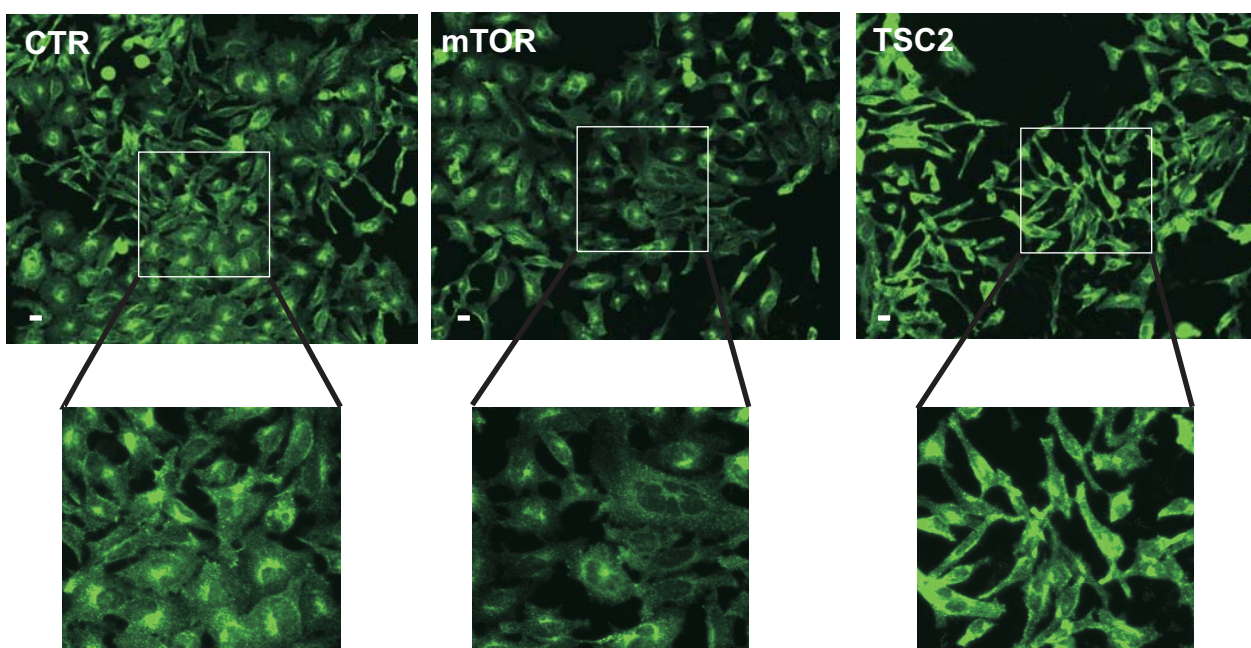

Figure S5

Supplement: Additional data file 1 — Figure S1 illustrates the observed well-to-well variability in the transferrin uptake assay. HeLa cells were transfected with CTR d-siRNA (targeting yellow fluorescent protein, eYFP) or d-siRNA targeting the AP2M1 gene. Twelve wells within the same row of a 96-well plate were transfected with the same pool of d-siRNAs to measure the well-to-well variability of the intensity measurements. Means ± standard deviation of two experiments are represented. Figure S2 summarizes the strategy used to generate the diced-siRNA library. Specific primers for the selected genes were designed to amplify approximately 600 bp PCR fragments from a cDNA library. A second amplification was performed with a set of nested primers bearing a T7 promoter sequence extension. Nested PCR products were in vitro transcribed, resulting double-stranded RNAs were diced with recombinant enzyme Dicer and the 21 mers d-siRNAs were finally purified from incomplete digests. Figure S3 shows the relationship between duplicated values of F for the 1,920 screened d-siRNAs (red). The data are compared to a simulated, two-dimensional Normal distribution of the stochastic noise (blue). The estimated standard deviation of the noise distribution is based on the measured deviations between duplicated measurements for the same d-siRNA. Figure S4 shows the distribution of the CAsH scores from the primary screen. The population of genes with CAsH >0.95 are indicated in red. Figure S5 is an example of the images used for quantitative analysis. (a) Images of HeLa cells illustrating the effect of d-siRNAs targeting mTOR (diced-mTOR) or GL3 luciferase (CTR) on fluorescent transferrin (red) uptake, as well as the effect of 24 h treatment with rapamycin (RAPA) compared to DMSO (CTR). Scale bars: 20 μm. Insets show a magnified view of the indicated image subset. (b) Immunofluorescent staining (green) of transferrin receptor performed on permeabilized cells in order to estimate the total number of transferrin receptors. Exam [file gb-2007-8-7-r142-S1.pdf]
