# Supplementary material for: siRNA screen of the human signaling proteome identifies the PtdIns(3,4,5)P3-mTOR signaling pathway as a primary regulator of transferrin uptake
Source: Genome Biol. 2007 Jul 19;8(7):R142. doi: 10.1186/gb-2007-8-7-r142 (PMC2323231; doi:10.1186/gb-2007-8-7-r142)
Supplement: Additional data file 2 — Genes targeted by the human d-siRNA signaling library. [file gb-2007-8-7-r142-S2.pdf]

Additional data file 2: List of the selected genes included in the human d-siRNA signaling library.

UniqueID	Gene	NP#	NM#	Conserved Domains
H01-0001	BTK	NP_000052	NM_000061	PH/BTK/SH3/SH2/TyrKc
H01-0002	CDK4	NP_000066	NM_000075	S_TKc
H01-0003	CDKN1C	NP_000067	NM_000076	CDI
H01-0004	CDKN2A	NP_000068	NM_000077	P19Arf_N
H01-0005	CHS1	NP_000072	NM_000081	perilipin/Beach/WD40
H01-0006	GSN	NP_000168	NM_000177	GEL
H01-0007	GUCY2D	NP_000171	NM_000180	ANF_receptor/pkinase/CYCc
H01-0008	JAK3	NP_000206	NM_000215	B41/SH2/TyrKc
H01-0009	MTM1	NP_000243	NM_000252	GRAM/PTPc_motif
H01-0010	MYL3	NP_000249	NM_000258	FRQ1
H01-0011	MYO5A	NP_000250	NM_000259	myosin_head
H01-0012	MYO7A	NP_000251	NM_000260	MYSc/MyTH4//B41
H01-0013	NF1	NP_000258	NM_000267	RasGAP/SEC14
H01-0014	NPHP1	NP_000263	NM_000272	SH3
H01-0015	OCRL	NP_000267	NM_000276	IPPc/RhoGAP
H01-0016	PHKG2	NP_000285	NM_000294	S_TKc
H01-0017	PTEN	NP_000305	NM_000314	CDC14
H01-0018	SPTB	NP_000338	NM_000347	SAC6/spectrin
H01-0019	WAS	NP_000368	NM_000377	WH1/PBD
H01-0020	CHM	NP_000381	NM_000390	GDI/GDI
H01-0021	GPD2	NP_000399	NM_000408	GlpA/FRQ1
H01-0022	GUCA1A	NP_000400	NM_000409	FRQ1
H01-0023	MYL2	NP_000423	NM_000432	FRQ1
H01-0024	NCF2	NP_000424	NM_000433	TPR/SH3/PB1
H01-0025	PDE6A	NP_000431	NM_000440	GAF/PDEase
H01-0026	PLEC1	NP_000436	NM_000445	SAC6/Smc/PLEC
H01-0027	SOD1	NP_000445	NM_000454	SPEC/SH3/FRQ1
H01-0028	STK11	NP_000446	NM_000455	S_TKc
H01-0029	TEK	NP_000450	NM_000459	laminin_EGF/IG/FN3/TyrKc
H01-0030	UBE3A	NP_000453	NM_000462	HECTc
H01-0031	RYR1	NP_000531	NM_000540	MIR/SPRY/RyR
H01-0032	NOS3	NP_000594	NM_000603	NO_synthase/CysJ
H01-0033	NOS1	NP_000611	NM_000620	PDZ/NO_synthase/CysJ
H01-0034	ANXA1	NP_000691	NM_000700	annexin
H01-0035	CACNB4	NP_000717	NM_000726	SH3/Ca_channel_B
H01-0036	GUCY1A2	NP_000846	NM_000855	guanylate_cyc
H01-0037	GUCY1A3	NP_000847	NM_000856	CYCc
H01-0038	GUCY1B3	NP_000848	NM_000857	guanylate_cyc
H01-0039	GUK1	NP_000849	NM_000858	GuKc
H01-0040	NQO1	NP_000894	NM_000903	Flavodoxin_2
H01-0041	PDE3A	NP_000912	NM_000921	PDEase
H01-0042	PDE3B	NP_000913	NM_000922	PDEase
H01-0043	PDE4C	NP_000914	NM_000923	PDEase
H01-0044	PDE1B	NP_000915	NM_000924	PDEase
H01-0045	PLCB3	NP_000923	NM_000932	PI-PLC/PI-PLC/C2/SbcC
H01-0046	PLCB4	NP_000924	NM_000933	PI-PLC/PLCc/C2/Smc
H01-0047	PPP3CA	NP_000935	NM_000944	PP2Ac

H01-0048	PPP3R1	NP_000936	NM_000945	FRQ1
H01-0049	RYR2	NP_001026	NM_001035	MIR/SPRY/RyR
H01-0050	RYR3	NP_001027	NM_001036	MIR/SPRY/RyR
H01-0051	ACTN1	NP_001093	NM_001102	SAC6
H01-0052	ACTN2	NP_001094	NM_001103	SAC6/spectrin
H01-0053	ACTN3	NP_001095	NM_001104	SAC6
H01-0054	ACVR1	NP_001096	NM_001105	Activin_recp/GS/pkinase
H01-0055	ACVR2B	NP_001097	NM_001106	Activin_recp/pkinase
H01-0056	ADCY7	NP_001105	NM_001114	guanylate_cyc
H01-0057	ADCY8	NP_001106	NM_001115	guanylate_cyc
H01-0058	ADCY9	NP_001107	NM_001116	guanylate_cyc
H01-0059	AP1G1	NP_001119	NM_001128	Adaptin_N/Alpha_adaptinC2
H01-0060	ANK2	NP_001139	NM_001148	Arp/ANK
H01-0061	ANXA4	NP_001144	NM_001153	annexin
H01-0062	ANXA5	NP_001145	NM_001154	annexin
H01-0063	APBA1	NP_001154	NM_001163	PID/PDZ
H01-0064	ARHGAP5	NP_001164	NM_001173	RAB/FF/RhoGAP
H01-0065	ARHGDIB	NP_001166	NM_001175	Rho_GDI
H01-0066	ARHGDIG	NP_001167	NM_001176	Rho_GDI
H01-0067	ARL1	NP_001168	NM_001177	arf
H01-0068	BMPR1B	NP_001194	NM_001203	Activin_recp/pkinase
H01-0069	BUB1B	NP_001202	NM_001211	S_TKc
H01-0070	CALU	NP_001210	NM_001219	FRQ1
H01-0071	CAV2	NP_001224	NM_001233	Caveolin
H01-0072	CCNA2	NP_001228	NM_001237	cyclin
H01-0073	CCNH	NP_001230	NM_001239	CCL1
H01-0074	CCNT1	NP_001231	NM_001240	CCL1
H01-0075	CDK3	NP_001249	NM_001258	S_TKc
H01-0076	CDK6	NP_001250	NM_001259	S_TKc
H01-0077	CDK8	NP_001251	NM_001260	S_TKc
H01-0078	CDK9	NP_001252	NM_001261	S_TKc
H01-0079	CHEK1	NP_001265	NM_001274	S_TKc
H01-0080	CHUK	NP_001269	NM_001278	S_TKc
H01-0081	AP2B1	NP_001273	NM_001282	Adaptin_N/Alpha_adaptinC2
H01-0082	CSNK1G2	NP_001310	NM_001319	pkinase
H01-0083	DGKA	NP_001336	NM_001345	FRQ1/DAG-bind/DAGKa
H01-0084	DGKG	NP_001337	NM_001346	FRQ1/DAG-bind/C1/DAGKa
H01-0085	DGKQ	NP_001338	NM_001347	DAG-bind/C1/RA/DAGKa
H01-0086	DAPK3	NP_001339	NM_001348	S_TKc
H01-0087	DLG2	NP_001355	NM_001364	PDZ/SH3/GuKc
H01-0088	DLG4	NP_001356	NM_001365	PDZ/SH3/GuKc
H01-0089	DOCK1	NP_001371	NM_001380	SH3
H01-0090	DOK1	NP_001372	NM_001381	IRS
H01-0091	DUSP9	NP_001386	NM_001395	DSPc
H01-0092	DYRK1A	NP_001387	NM_001396	S_TKc
H01-0093	ERN1	NP_001424	NM_001433	S_TKc
H01-0094	FLNA	NP_001447	NM_001456	SAC6/Filamin/IG_FLMN
H01-0095	FLNB	NP_001448	NM_001457	SAC6/IG_FLMN/Filamin
H01-0096	FYB	NP_001456	NM_001465	SH3
H01-0097	GDI1	NP_001484	NM_001493	GDI
H01-0098	GDI2	NP_001485	NM_001494	GDI

H01-0099	GUCY2F	NP_001513	NM_001522	ANF_receptor/TyrKc/CYCc
H01-0100	INPPL1	NP_001558	NM_001567	SH2/IPPc/SAM
H01-0101	IRAK1	NP_001560	NM_001569	pkinase
H01-0102	IRAK2	NP_001561	NM_001570	death/pkinase
H01-0103	ACVR2	NP_001607	NM_001616	Activin_recp/pkinase
H01-0104	ADRBK1	NP_001610	NM_001619	RGS/S_TKc/PH
H01-0105	AIF1	NP_001614	NM_001623	FRQ1
H01-0106	AKT2	NP_001617	NM_001626	PH/S_TKc
H01-0107	ANXA8	NP_001621	NM_001630	annexin
H01-0108	APXL	NP_001640	NM_001649	PDZ/DUF164
H01-0109	ARAF1	NP_001645	NM_001654	RBD/DAG_PE-bind/pkinase
H01-0110	ARCN1	NP_001646	NM_001655	Adap_comp_sub
H01-0111	ARF1	NP_001649	NM_001658	ARF
H01-0112	ARF3	NP_001650	NM_001659	ARF
H01-0113	ARF4	NP_001651	NM_001660	ARF
H01-0114	ARF4L	NP_001652	NM_001661	arf
H01-0115	ARF5	NP_001653	NM_001662	ARF
H01-0116	ARF6	NP_001654	NM_001663	ARF/SAR
H01-0117	RHOA	NP_001655	NM_001664	RHO
H01-0118	ARHGAP4	NP_001657	NM_001666	FCH/RhoGAP/SH3
H01-0119	ARL2	NP_001658	NM_001667	arf
H01-0120	AXL	NP_001690	NM_001699	IG/FN3/TyrKc
H01-0121	BMX	NP_001712.1	NM_001721	PH/BTK/SH2/TyrKc
H01-0122	CALM2	NP_001734	NM_001743	FRQ1
H01-0123	CAMK4	NP_001735	NM_001744	S_TKc
H01-0124	CAPG	NP_001738	NM_001747	GEL
H01-0125	CAPN2	NP_001739	NM_001748	Calpain_III/FRQ1
H01-0126	CAV1	NP_001744	NM_001753	Caveolin
H01-0127	CCND1	NP_001749	NM_053056	cyclin
H01-0128	CCND2	NP_001750	NM_001759	cyclin
H01-0129	CCND3	NP_001751	NM_001760	cyclin
H01-0130	CDK7	NP_001790	NM_001799	S_TKc
H01-0131	CENPE	NP_001804	NM_001813	KISc/Smc
H01-0132	CHML	NP_001812	NM_001821	GDI/GDI
H01-0133	CHN1	NP_001813	NM_001822	SH2/DAG_PE-bind/RhoGAP
H01-0134	CSNK1A1	NP_001883	NM_001892	pkinase
H01-0135	CTNNA1	NP_001894	NM_001903	Vinculin
H01-0136	MPP3	NP_001923	NM_001932	L27/PDZ/SH3/GuKc
H01-0137	DRP2	NP_001930	NM_001939	SPEC//WW/zz
H01-0138	DDR1	NP_001945	NM_001954	F5_F8_type_C/TyrKc
H01-0139	FES	NP_001996	NM_002005	FCH/SH2/TyrKc
H01-0140	FLT1	NP_002010	NM_002019	IG/TyrKc
H01-0141	FRK	NP_002022	NM_002031	SH3/SH2/TyrKc
H01-0142	GAB1	NP_002030	NM_002039	PH
H01-0143	GAP43	NP_002036	NM_002045	IQ
H01-0144	GNA11	NP_002058	NM_002067	G-alpha
H01-0145	GNA15	NP_002059	NM_002068	G-alpha
H01-0146	GNAI1	NP_002060	NM_002069	G-alpha
H01-0147	GNAI2	NP_002061	NM_002070	G-alpha
H01-0148	GNAL	NP_002062	NM_002071	G-alpha
H01-0149	GNAQ	NP_002063	NM_002072	G-alpha

H01-0150	GNAZ	NP_002064	NM_002073	G-alpha
H01-0151	GNB1	NP_002065	NM_002074	COG2319
H01-0152	GRK6	NP_002073	NM_002082	RGS/S_TKc
H01-0153	GRB2	NP_002077	NM_002086	SH3/SH2
H01-0154	GUCA1B	NP_002089	NM_002098	FRQ1
H01-0155	HCK	NP_002101	NM_002110	SH3/SH2/TyrKc
H01-0156	HPCA	NP_002134	NM_002143	FRQ1
H01-0157	ITPKA	NP_002211	NM_002220	IPK
H01-0158	ITPKB	NP_002212	NM_002221	IPK
H01-0159	ITPR1	NP_002213	NM_002222	MIR/PMT1/RYDR_ITPR
H01-0160	ITPR2	NP_002214	NM_002223	MIR/MIR/RYDR_ITPR
H01-0161	ITPR3	NP_002215	NM_002224	MIR/MIR/RYDR_ITPR
H01-0162	JAK1	NP_002218	NM_002227	B41/SH2/pkinase/TyrKc
H01-0163	KDR	NP_002244	NM_002253	IG/IGc2/IG/IG/TyrKc/TyrKc
H01-0164	KIF3C	NP_002245	NM_002254	kinesin
H01-0165	KIFC1	NP_002254	NM_002263	KISc
H01-0166	KPNA2	NP_002257	NM_002266	SRP1
H01-0167	LCP1	NP_002289	NM_002298	SAC6/CH
H01-0168	LTK	NP_002335	NM_002344	TyrKc
H01-0169	LYN	NP_002341	NM_002350	SH3/SH2/TyrKc
H01-0170	MARK3	NP_002367	NM_002376	S_TKc/KA1
H01-0171	MAP3K3	NP_002392	NM_002401	PB1/S_TKc
H01-0172	MAP3K11	NP_002410	NM_002419	SH3/TyrKc
H01-0173	MPP1	NP_002427	NM_002436	PDZ/SH3/GuKc
H01-0174	MSN	NP_002435	NM_002444	Band_41/ERM
H01-0175	MAP3K10	NP_002437	NM_002446	SH3/TyrKc
H01-0176	MX1	NP_002453	NM_002462	dynamin_2/GED
H01-0177	MX2	NP_002454	NM_002463	dynamin_2
H01-0178	MYH9	NP_002464	NM_002473	myosin_head/Myosin_tail
H01-0179	MYH11	NP_002465	NM_002474	myosin_head
H01-0180	MLC1SA	NP_002466	NM_002475	FRQ1
H01-0181	MYL5	NP_002468	NM_002477	FRQ1
H01-0182	PPP1R12A	NP_002471	NM_002480	Arp
H01-0183	PPP1R12B	NP_002472	NM_002481	Arp
H01-0184	NDUFS4	NP_002486	NM_002495	ETC_C1_NDUFA4
H01-0185	NEK2	NP_002488	NM_002497	S_TKc
H01-0186	NTRK3	NP_002521	NM_002530	LRRCT/IG/IGc2/TyrKc
H01-0187	OPHN1	NP_002538	NM_002547	PH/RhoGAP
H01-0188	OSBP	NP_002547	NM_002556	PH/Oxysterol_BP
H01-0189	PAK1	NP_002567	NM_002576	PBD/S_TKc
H01-0190	PAK3	NP_002569	NM_002578	PBD/S_TKc
H01-0191	PCTK2	NP_002586	NM_002595	S_TKc
H01-0192	PDE2A	NP_002590	NM_002599	GAF/PDEase
H01-0193	PDE4B	NP_002591	NM_002600	PDEase
H01-0194	PDE7A	NP_002594	NM_002603	PDEase
H01-0195	PDE9A	NP_002597	NM_002606	PDEase
H01-0196	PDPK1	NP_002604	NM_002613	S_TKc
H01-0197	PDZK1	NP_002605	NM_002614	PDZ
H01-0198	PEX13	NP_002609	NM_002618	Peroxin-13_N/SH3
H01-0199	PFN2	NP_002619	NM_002628	profilin
H01-0200	PIK3C2A	NP_002636	NM_002645	PI3K/PX/C2

H01-0201	PIK3C2B	NP_002637	NM_002646	PI3K/PX/C2
H01-0202	PIK3C3	NP_002638	NM_002647	PI3K
H01-0203	PIM1	NP_002639	NM_002648	pkinase
H01-0204	PIK3CG	NP_002640	NM_002649	PI3K
H01-0205	PIK4CB	NP_002642	NM_002651	PI3Kc
H01-0206	PLCG1	NP_002651	NM_002660	PI-PLC/SH2/SH3/PH/C2/PLEC
H01-0207	PLCG2	NP_002652	NM_002661	PLC/SH2/SH3/PH
H01-0208	PLD1	NP_002653	NM_002662	PX/PH/Cls
H01-0209	PLD2	NP_002654	NM_002663	PX/Cls
H01-0210	PLEK	NP_002655	NM_002664	PH/DEP/PH
H01-0211	PLS1	NP_002661	NM_002670	SAC6
H01-0212	PPP1CA	NP_002699	NM_002708	PP2Ac
H01-0213	PPP1CB	NP_002700	NM_002709	PP2Ac
H01-0214	PPP1CC	NP_002701	NM_002710	PP2Ac
H01-0215	PPP1R7	NP_002703	NM_002712	COG4886
H01-0216	PPP1R10	NP_002705	NM_002714	TFS2N/zf-CCCH
H01-0217	PPP2CA	NP_002706	NM_002715	PP2Ac
H01-0218	PPP2R1B	NP_002707	NM_002716	HEAT
H01-0219	PPP2R2A	NP_002708	NM_002717	CDC55
H01-0220	PPP2R3A	NP_002709	NM_002718	
H01-0221	PPP2R5C	NP_002710	NM_002719	B56
H01-0222	PPP4C	NP_002711	NM_002720	PP2Ac
H01-0223	PPP6C	NP_002712	NM_002721	PP2Ac
H01-0224	PRKACA	NP_002721	NM_002730	S_TKc
H01-0225	PRKACB	NP_002722	NM_002731	S_TKc
H01-0226	PRKACG	NP_002723	NM_002732	S_TKc
H01-0227	PRKAG1	NP_002724	NM_002733	CBS
H01-0228	PRKAR1A	NP_002725	NM_002734	RIIa/cNMP_binding
H01-0229	PRKAR2B	NP_002727	NM_002736	RIIa/cNMP
H01-0230	PRKCA	NP_002728	NM_002737	DAG-bind/C2/S_TKc
H01-0231	PRKCB1	NP_002729	NM_002738	DAG-bind/S_TKc
H01-0232	PRKCG	NP_002730	NM_002739	DAG-bind/C2/S_TKc
H01-0233	PRKCI	NP_002731	NM_002740	PB1/DAG_PE-bind/S_TKc
H01-0234	PKN1	NP_002732	NM_002741	HR1/S_TKc
H01-0235	PRKD1	NP_002733	NM_002742	DAG-bind/PH/S_TKc
H01-0236	PRKCZ	NP_002735	NM_002744	PB1/DAG_PE-bind/S_TKc
H01-0237	MAPK4	NP_002738	NM_002747	S_TKc
H01-0238	MAPK6	NP_002739	NM_002748	S_TKc
H01-0239	MAPK11	NP_002742	NM_002751	S_TKc
H01-0240	MAPK13	NP_002745	NM_002754	S_TKc
H01-0241	MAP2K1	NP_002746	NM_002755	S_TKc
H01-0242	EIF2AK2	NP_002750	NM_002759	DSRM/S_TKc
H01-0243	PRKY	NP_002751	NM_002760	S_TKc
H01-0244	PSD	NP_002770	NM_002779	Sec7/PH
H01-0245	PTPN1	NP_002818	NM_002827	PTPc
H01-0246	PTPN3	NP_002820	NM_002829	Band_41/PDZ/PTPc
H01-0247	PTPN4	NP_002821	NM_002830	Band_41/PDZ/PTPc
H01-0248	PTPN9	NP_002824	NM_002833	CRAL_TRIO/PTPc
H01-0249	PTPN12	NP_002826	NM_002835	PTPc
H01-0250	PTPRH	NP_002833	NM_002842	FN3/PTPc
H01-0251	PTPRJ	NP_002834	NM_002843	FN3/PTPc

H01-0252	PTPRK	NP_002835	NM_002844	MAM/IG/FN3//PTPc
H01-0253	PTPRM	NP_002836	NM_002845	MAM/IG/FN3//PTPc
H01-0254	PTPRN	NP_002837	NM_002846	PTPc
H01-0255	PTPRZ1	NP_002842	NM_002851	carb_anhydrase/fn3/PTPc
H01-0256	RAB2	NP_002856	NM_002865	RAB
H01-0257	RAB3A	NP_002857	NM_002866	RAB
H01-0258	RAB3B	NP_002858	NM_002867	RAB
H01-0259	RAB5B	NP_002859	NM_002868	ras
H01-0260	RAB6A	NP_002860	NM_002869	RAB
H01-0261	RAB13	NP_002861	NM_002870	RAB
H01-0262	RAC2	NP_002863	NM_002872	RHO
H01-0263	RAF1	NP_002871	NM_002880	RBD/DAG-bind/SPS1/pkinase
H01-0264	RALB	NP_002872	NM_002881	RAS
H01-0265	RANGAP1	NP_002874	NM_002883	RNA1
H01-0266	RAP1A	NP_002875	NM_002884	RAS
H01-0267	RAP1GA1	NP_002876	NM_002885	GoLoco/Rap_GAP
H01-0268	RCV1	NP_002894	NM_002903	FRQ1
H01-0269	RDX	NP_002897	NM_002906	Band_41/ERM
H01-0270	RGS1	NP_002913	NM_002922	RGS
H01-0271	RGS2	NP_002914	NM_002923	RGS
H01-0272	RGS10	NP_002916	NM_002925	RGS
H01-0273	RGS12	NP_002917	NM_002926	PDZ/PID/RGS/RBD
H01-0274	RGS16	NP_002919	NM_002928	RGS
H01-0275	GRK1	NP_002920	NM_002929	RGS/S_TKc
H01-0276	RIT2	NP_002921	NM_002930	RAS
H01-0277	ROS1	NP_002935	NM_002944	FN3/TyrKc
H01-0278	RPS6KA1	NP_002944	NM_002953	S_TKc/S_TKc
H01-0279	RYK	NP_002949	NM_002958	WIF/TyrKc
H01-0280	MAPK12	NP_002960	NM_002969	S_TKc
H01-0281	MAP2K4	NP_003001	NM_003010	S_TKc
H01-0282	SH3BP2	NP_003014	NM_003023	PH/SH2
H01-0283	ITSN1	NP_003015	NM_003024	EH/Smc/SH3/RhoGEF/C2
H01-0284	SH3GL1	NP_003016	NM_003025	BAR/SH3
H01-0285	SH3GL2	NP_003017	NM_003026	BAR/SH3
H01-0286	SH3GL3	NP_003018	NM_003027	BAR/SH3
H01-0287	SHB	NP_003019	NM_003028	SH2
H01-0288	SHC1	NP_003020	NM_003029	PID/SH2
H01-0289	SNTA1	NP_003089	NM_003098	PDZ/PH
H01-0290	SPTA1	NP_003117	NM_003126	spectrin
H01-0291	SPTAN1	NP_003118	NM_003127	spectrin
H01-0292	SRI	NP_003121	NM_003130	FRQ1
H01-0293	SRPK1	NP_003128	NM_003137	S_TKc/S_TKc
H01-0294	SRPK2	NP_003129	NM_003138	S_TKc/S_TKc
H01-0295	STAC	NP_003140	NM_003149	DAG_PE-bind/SH3
H01-0296	STAT4	NP_003142	NM_003151	STAT/STAT_bind/SH2
H01-0297	STAT5A	NP_003143	NM_003152	STAT/STAT_bind/SH2
H01-0298	STAT6	NP_003144	NM_003153	STAT/STAT_bind/SH2
H01-0299	STIM1	NP_003147	NM_003156	SAM/Smc
H01-0300	NEK4	NP_003148	NM_003157	S_TKc
H01-0301	STK6	NP_003149	NM_003600	S_TKc
H01-0302	CDKL5	NP_003150	NM_003159	S_TKc

H01-0303	AURKC	NP_003151	NM_003160	S_TKc
H01-0304	RPS6KB1	NP_003152	NM_003161	S_TKc
H01-0305	STX5A	NP_003155	NM_003164	t_SNARE
H01-0306	STXBP1	NP_003156	NM_003165	Sec1
H01-0307	SYK	NP_003168	NM_003177	SH2/TyrKc
H01-0308	SYN2	NP_003169	NM_003178	Synapsin
H01-0309	SYT5	NP_003171	NM_003180	C2
H01-0310	TEC	NP_003206	NM_003215	PH/BTK/SH3/SH2/TyrKc
H01-0311	ARFRP1	NP_003215	NM_003224	arf
H01-0312	TGFBR2	NP_003233	NM_003242	pkinase
H01-0313	TIAM1	NP_003244	NM_003253	PH/RBD/PDZ/RhoGEF
H01-0314	TNNC2	NP_003270	NM_003279	FRQ1
H01-0315	TNNC1	NP_003271	NM_003280	FRQ1
H01-0316	PHLDA2	NP_003302	NM_003311	PH
H01-0317	TTK	NP_003309	NM_003318	S_TKc
H01-0318	TXK	NP_003319	NM_003328	SH3/SH2/TyrKc
H01-0319	TYK2	NP_003322	NM_003331	B41/SH2/TyrKc/
H01-0320	UBE1L	NP_003326	NM_003335	ThiF/UBACT
H01-0321	UBE2A	NP_003327	NM_003336	COG5078
H01-0322	UBE2B	NP_003328	NM_003337	COG5078
H01-0323	UBE2D1	NP_003329	NM_003338	COG5078
H01-0324	UBE2D2	NP_003330	NM_003339	COG5078
H01-0325	UBE2E1	NP_003332	NM_003341	COG5078
H01-0326	UBE2G1	NP_003333	NM_003342	COG5078
H01-0327	UBE2G2	NP_003334	NM_003343	COG5078
H01-0328	UBE2H	NP_003335	NM_003344	UBCc
H01-0329	UBE2L3	NP_003338	NM_003347	UBCc
H01-0330	UBE2N	NP_003339	NM_003348	COG5078
H01-0331	UBE2V2	NP_003341	NM_003350	UQ_con
H01-0332	USP4	NP_003354	NM_003363	DUSP/UBP12
H01-0333	VASP	NP_003361	NM_003370	WH1
H01-0334	VAV2	NP_003362	NM_003371	CH/RhoGEF/PH/DAG-bind/SH3/SH2
H01-0335	VIL2	NP_003370	NM_003379	Band_41/ERM
H01-0336	VRK1	NP_003375	NM_003384	pkinase
H01-0337	VSNL1	NP_003376	NM_003385	FRQ1
H01-0338	WASPIP	NP_003378	NM_003387	
H01-0339	WEE1	NP_003381	NM_003390	pkinase
H01-0340	YWHAH	NP_003396	NM_003405	14-3-3
H01-0341	PTP4A1	NP_003454	NM_003463	CDC14
H01-0342	USP7	NP_003461	NM_003470	COG5077
H01-0343	STAM	NP_003464	NM_003473	VHS/SH3
H01-0344	RASSF7	NP_003466	NM_003475	RA
H01-0345	USP5	NP_003472	NM_003481	UBP14
H01-0346	DYSF	NP_003485	NM_003494	C2/DysFN
H01-0347	AXIN1	NP_003493	NM_003502	RGS/DIX
H01-0348	CDC7	NP_003494	NM_003503	S_TKc
H01-0349	PIP5K1A	NP_003548	NM_003557	PIPKc
H01-0350	PIP5K1B	NP_003549	NM_003558	PIP5K
H01-0351	PIP5K2B	NP_003550	NM_003559	PIPKc
H01-0352	PLA2G6	NP_003551	NM_003560	Arp/Patatin
H01-0353	ULK1	NP_003556	NM_003565	S_TKc

H01-0354	EEA1	NP_003557	NM_003566	Smc/FYVE
H01-0355	BCAR3	NP_003558	NM_003567	SH2/RasGEF
H01-0356	ANXA9	NP_003559	NM_003568	annexin
H01-0357	STK24	NP_003567	NM_003576	S_TKc
H01-0358	NCK2	NP_003572	NM_003581	SH3/SH2
H01-0359	DYRK3	NP_003573	NM_003582	S_TKc
H01-0360	DUSP11	NP_003575	NM_003584	DSPc
H01-0361	DOC2B	NP_003576	NM_003585	C2
H01-0362	DOC2A	NP_003577	NM_003586	C2
H01-0363	IRS4	NP_003595	NM_003604	PH/IRS
H01-0364	CDC42BPA	NP_003598	NM_003607	S_TKc/SbcC/DAG-bind/PH/CNH/PBD
H01-0365	RGS5	NP_003608	NM_003617	RGS
H01-0366	MAP4K3	NP_003609	NM_003618	S_TKc/CNH
H01-0367	PPM1D	NP_003611	NM_003620	PP2Cc
H01-0368	PPFIA2	NP_003616	NM_003625	Smc/SAM
H01-0369	PIK3R3	NP_003620	NM_003629	SH2/SH2
H01-0370	DGKZ	NP_003637	NM_003646	C1/DAGKa/Arp
H01-0371	DGKE	NP_003638	NM_003647	DAG-bind/DAGKa
H01-0372	CAMK1	NP_003647	NM_003656	S_TKc
H01-0372	MKNK1	NP_003675.2	NM_003684	S_TKc
H01-0374	PDLIM4	NP_003678	NM_003687	PDZ/LIM
H01-0375	CASK	NP_003679	NM_003688	S_TKc/L27/PDZ/SH3/GuKc
H01-0376	PRKRA	NP_003681	NM_003690	DSRM/dsrm
H01-0377	STK16	NP_003682	NM_003691	pkinase
H01-0378	RGS20	NP_003693	NM_003702	RGS
H01-0379	PLA2G4C	NP_003697	NM_003706	PLA2_B
H01-0380	VDP	NP_003706	NM_003715	Smc
H01-0381	TP73L	NP_003713	NM_003722	P53/SAM
H01-0382	SCAP1	NP_003717	NM_003726	PH/SH3
H01-0383	SOCS1	NP_003736	NM_003745	SH2/SOCS
H01-0384	TNKS	NP_003738	NM_003747	Arp/SAM/PARP
H01-0385	VAMP4	NP_003753	NM_003762	synaptobrevin
H01-0386	STX16	NP_003754	NM_003763	COG5325
H01-0387	STX11	NP_003755	NM_003764	SynN
H01-0388	STX10	NP_003756	NM_003765	t_SNARE
H01-0389	CTNNAL1	NP_003789	NM_003798	Vinculin
H01-0390	RNGTT	NP_003791	NM_003800	DSPc
H01-0391	RIPK1	NP_003795	NM_003804	TyrKc/death
H01-0392	RIPK2	NP_003812	NM_003821	pkinase/CARD
H01-0393	NAPG	NP_003817	NM_003826	
H01-0394	NAPA	NP_003818	NM_003827	
H01-0395	MPDZ	NP_003820	NM_003829	PDZ
H01-0396	RGS9	NP_003826	NM_003835	GGL/RGS
H01-0397	DYRK4	NP_003836	NM_003845	S_TKc
H01-0398	INPP4B	NP_003857	NM_003866	
H01-0399	IQGAP1	NP_003861	NM_003870	IQG1/WW/RasGAP
H01-0400	SOCS2	NP_003868	NM_003877	SH2/SOCS
H01-0401	CDK5R1	NP_003876	NM_003885	CDK5_activator
H01-0402	DDEF2	NP_003878	NM_003887	PH/ArfGap/SH3
H01-0403	SYNJ1	NP_003886	NM_003895	Syja_N/IPPc
H01-0404	SYNJ2	NP_003889	NM_003898	Syja_N

H01-0405	ARHGEF7	NP_003890	NM_003899	SH3/RhoGEF/PH/
H01-0406	CPNE3	NP_003900	NM_003909	C2/VWA
H01-0407	MTMR2	NP_003903	NM_016156	GRAM/PTPc_motif
H01-0408	PRPF4B	NP_003904	NM_003913	S_TKc
H01-0409	RAB7L1	NP_003920	NM_003929	RAB
H01-0410	WASF1	NP_003922	NM_003931	WH2
H01-0411	BAIAP3	NP_003924	NM_003933	C2
H01-0412	CDK5R2	NP_003927	NM_003936	CDK5_activator
H01-0413	AP3D1	NP_003929	NM_003938	Adaptin_N
H01-0414	USP13	NP_003931	NM_003940	UBP14
H01-0415	RPS6KA4	NP_003933	NM_003942	S_TKc/S_TKc
H01-0416	HAPIP	NP_003938	NM_003947	SEC14/SPEC/RhoGEF/PH
H01-0417	RPS6KB2	NP_003943	NM_003952	S_TKc
H01-0418	MAP3K14	NP_003945	NM_003954	S_TKc
H01-0419	SOCS3	NP_003946	NM_003955	SH2/SOCS
H01-0420	STK29	NP_003948	NM_003957	S_TKc
H01-0421	UBE1C	NP_003959	NM_003968	ThiF/UBACT
H01-0422	DOK2	NP_003965	NM_003974	PH/PTBI
H01-0423	SH2D2A	NP_003966	NM_003975	SH2
H01-0424	PSTPIP1	NP_003969	NM_003978	FCH/SH3
H01-0425	TNK1	NP_003976	NM_003985	TyrKc
H01-0426	DMD	NP_004001	NM_004010	CH/spectrin/SbcC//WW
H01-0427	INPP4A	NP_004018	NM_004027	
H01-0428	ANXA6	NP_004024	NM_004033	annexin
H01-0429	ANXA7	NP_004025	NM_004034	
H01-0430	ADCY3	NP_004027	NM_004036	guanylate_cyc
H01-0431	ANXA2	NP_004030	NM_004039	annexin
H01-0432	RHOB	NP_004031	NM_004040	RHO
H01-0433	CCNG1	NP_004051	NM_004060	cyclin/CYCLIN
H01-0434	CDKN1B	NP_004055.1	NM_004064	CDI
H01-0435	CETN1	NP_004057.1	NM_004066	FRQ1
H01-0436	CHN2	NP_004058	NM_004067	SH2/DAG_PE-bind/RhoGAP
H01-0437	AP2M1	NP_004059	NM_004068	Adap_comp_sub
H01-0438	CLK1	NP_004062	NM_004071	S_TKc
H01-0439	PLK3	NP_004064	NM_004073	S_TKc/POLO_box
H01-0440	DGKB	NP_004071	NM_004080	FRQ1/DAGbinding
H01-0441	DCTN1	NP_004073	NM_004082	NIP100/CAP_GLY/Smc
H01-0442	DLG1	NP_004078	NM_004087	PDZ/SH3/GuKc
H01-0443	DUSP3	NP_004081.1	NM_004090	DSPc
H01-0444	FLT3	NP_004110	NM_004119	ig/TyrKc
H01-0445	GMFB	NP_004115	NM_004124	cofilin_ADF
H01-0446	GUCY1B2	NP_004120	NM_004129	CYCc
H01-0447	MYO9B	NP_004136	NM_004145	RA/MYSc/C1/RhoGAP
H01-0448	PPP2CB	NP_004147	NM_004156	PP2Ac
H01-0449	PRKAR2A	NP_004148	NM_004157	RIIa/cNMP_binding
H01-0450	RAB1A	NP_004152	NM_004161	ras
H01-0451	RAB5A	NP_004153	NM_004162	ras
H01-0452	RAB27B	NP_004154	NM_004163	ras
H01-0453	RRAD	NP_004156	NM_004165	RAS
H01-0454	STX3A	NP_004168	NM_004177	Syntaxin
H01-0455	UCHL1	NP_004172	NM_004181	Peptidase_C12

H01-0456	CDKL1	NP_004187	NM_004196	S_TKc
H01-0457	STK19	NP_004188	NM_004197	
H01-0458	PKMYT1	NP_004194	NM_004203	S_TKc
H01-0459	AURKB	NP_004208	NM_004217	S_TKc
H01-0460	RAB11B	NP_004209	NM_004218	RAB
H01-0461	UBE2L6	NP_004214	NM_004223	UQ_con
H01-0462	STK17B	NP_004217	NM_004226	S_TKc
H01-0463	PSCD3	NP_004218	NM_004227	Sec7/PH
H01-0464	SOCS6	NP_004223	NM_004232	SH2/SOCS
H01-0465	TRIP12	NP_004229	NM_004238	SRP1/WWE/HECTc
H01-0466	TRIP10	NP_004231	NM_004240	FCH/SH3
H01-0467	RAB28	NP_004240	NM_004249	RAB
H01-0468	RAB9A	NP_004242	NM_004251	ras
H01-0469	HOMER1	NP_004263	NM_004272	WH1/Smc
H01-0470	AKAP6	NP_004265	NM_004274	spectrin
H01-0471	CABP1	NP_004267	NM_004276	FRQ1
H01-0472	RAB3D	NP_004274	NM_004283	RAB
H01-0473	PSCDBP	NP_004279	NM_004288	PDZ
H01-0474	RIN1	NP_004283	NM_004292	SH2/VPS9/RA
H01-0475	GNA14	NP_004288	NM_004297	G-alpha
H01-0476	ALK	NP_004295	NM_004304	MAM/TyrKc
H01-0477	ANXA13	NP_004297	NM_004306	annexin
H01-0478	ARHGAP1	NP_004299	NM_004308	SEC14/RhoGAP
H01-0479	ARHGDIA	NP_004300	NM_004309	Rho_GDI
H01-0480	RHOH	NP_004301	NM_004310	RHO
H01-0481	ARL3	NP_004302	NM_004311	arf
H01-0482	ARRB2	NP_004304	NM_004313	arrestin/arrestin_C
H01-0483	KIF1A	NP_004312	NM_004321	kinesin/FHA/PH
H01-0484	BMPR1A	NP_004320	NM_004329	Activin_recp/pkinase
H01-0485	BRAF	NP_004324	NM_004333	RBD/DAG_PE-bind/pkinase
H01-0486	BUB1	NP_004327	NM_004336	S_TKc
H01-0487	CETN2	NP_004335	NM_004344	FRQ1
H01-0488	CETN3	NP_004356	NM_004365	FRQ1
H01-0489	CSK	NP_004374	NM_004383	SH3/SH2/TyrKc
H01-0490	CSNK1G3	NP_004375	NM_004384	pkinase
H01-0491	CTNNA2	NP_004380	NM_004389	Vinculin
H01-0492	DBN1	NP_004386	NM_004395	cofilin_ADF
H01-0493	DNM1	NP_004399	NM_004408	dynamin_2/PH/GED
H01-0494	DUSP1	NP_004408	NM_004417	RHOD/DSPc/CDC14
H01-0495	DUSP2	NP_004409	NM_004418	Rhodanese/DSPc
H01-0496	DUSP5	NP_004410	NM_004419	RHOD/DSPc
H01-0497	DUSP8	NP_004411	NM_004420	RHOD/DSPc
H01-0498	DVL1	NP_004412	NM_004421	DIX/PDZ/DEP
H01-0499	DVL2	NP_004413	NM_004422	DIX/Dishevelled/PDZ/DEP
H01-0500	DVL3	NP_004414	NM_004423	DIX/Dishevelled/PDZ/DEP
H01-0501	PHC2	NP_004418	NM_004427	SAM
H01-0502	EPHA2	NP_004422	NM_004431	EPH_lbd/FN3//TyrKc/SAM
H01-0503	EPHA4	NP_004429	NM_004438	EPH_lbd/FN3//TyrKc/SAM
H01-0504	EPHA5	NP_004430	NM_004439	EPH_lbd/FN3/TyrKc
H01-0505	EPHB3	NP_004434	NM_004443	EPH_lbd/VSP/fn3/TyrKc/SAM
H01-0506	EPHB4	NP_004435	NM_004444	EPH_lbd/VSP/FN3TyrKc/SAM

H01-0507	EPS8	NP_004438	NM_004447	PTB/SH3
H01-0508	FGD1	NP_004454	NM_004463	RhoGEF/FYVE/PH
H01-0509	GRB14	NP_004481	NM_004490	RA/PH/SH2
H01-0510	GRLF1	NP_004482	NM_004491	ras/FF/RhoGAP
H01-0511	HRB	NP_004495	NM_004504	ArfGap
H01-0512	USP6	NP_004496	NM_004505	
H01-0513	ILK	NP_004508	NM_004517	Arp/pkinase
H01-0514	KIF2	NP_004511	NM_004520	KISc
H01-0515	KIF5B	NP_004512	NM_004521	kinesin/Smc
H01-0516	KIF5C	NP_004513	NM_004522	kinesin/Smc
H01-0517	KIF11	NP_004514	NM_004523	KISc
H01-0518	NEB	NP_004534	NM_004543	Nebulin/SH3
H01-0519	PIK3C2G	NP_004561	NM_004570	PI3K
H01-0520	PLCB2	NP_004564	NM_004573	PI-PLC-X/PI-PLC-Y/C2/Smc
H01-0521	PPP2R2B	NP_004567	NM_004576	CDC55
H01-0522	RAB4A	NP_004569	NM_004578	RAB
H01-0523	MAP4K2	NP_004570	NM_004579	S_TKc/CNH
H01-0524	RAB27A	NP_004571	NM_004580	ras
H01-0525	RABGGTA	NP_004572	NM_004581	BET4
H01-0526	RABGGTB	NP_004573	NM_004582	CAL1
H01-0527	RAB5C	NP_004574	NM_004583	ras
H01-0528	RPS6KA3	NP_004577	NM_004586	S_TKc/S_TKc
H01-0529	STX1A	NP_004594	NM_004603	Syntaxin
H01-0530	TGFBR1	NP_004603	NM_004612	Activin_recp/GS/pkinase
H01-0531	MAPKAPK3	NP_004626	NM_004635	S_TKc
H01-0532	RAB7	NP_004628	NM_004637	
H01-0533	AP3B2	NP_004635	NM_004644	Adaptin_N
H01-0534	USP9X	NP_004643	NM_004652	COG5077
H01-0535	AXIN2	NP_004646	NM_004655	RGS/DAX
H01-0536	RASAL1	NP_004649	NM_004658	C2/RasGAP/BTK
H01-0537	RAB11A	NP_004654	NM_004663	RAB
H01-0538	LIN7A	NP_004655	NM_004664	L27/PDZ
H01-0539	MTMR6	NP_004676	NM_004685	PTPc_motif
H01-0540	MTMR4	NP_004678	NM_004687	PTPc_motif/FYVE
H01-0541	LATS1	NP_004681	NM_004690	UBA/S_TKc
H01-0542	CCNE2	NP_004693	NM_004702	cyclin
H01-0543	RABEP1	NP_004694	NM_004703	Rabaptin/Rabaptin
H01-0544	HGS	NP_004703	NM_004712	VHS/FYVE
H01-0545	DGKI	NP_004708	NM_004717	DAGKa/Arp
H01-0546	MAP3K13	NP_004712	NM_004721	pkinase
H01-0547	AP4M1	NP_004713	NM_004722	Adap_comp_sub
H01-0548	DCAMKL1	NP_004725	NM_004734	DCX/DCX/S_TKc
H01-0549	BAIAP1	NP_004733	NM_004742	GuKc/WW/PDZ
H01-0550	DLGAP2	NP_004736	NM_004745	GKAP
H01-0551	DLG5	NP_004738	NM_004747	SbcC/PDZ/GuKc
H01-0552	RPS6KA5	NP_004746	NM_004755	S_TKc/S_TKc
H01-0553	STK17A	NP_004751	NM_004760	S_TKc
H01-0554	RGL2	NP_004752	NM_004761	RasGEF/RA
H01-0555	SNAP29	NP_004773	NM_004782	t_SNARE
H01-0556	TAOK2	NP_004774	NM_004783	S_TKc
H01-0557	UBE4A	NP_004779	NM_004788	UFD2

H01-0558	RAB33A	NP_004785	NM_004794	RAB
H01-0559	KIF3B	NP_004789	NM_004798	kinesin
H01-0560	OTOF	NP_004793	NM_004802	C2
H01-0561	GRAP2	NP_004801	NM_004810	SH3/SH2
H01-0562	PARG1	NP_004806	NM_004815	C1/RhoGAP
H01-0563	TJP2	NP_004808	NM_004817	PDZ/GuKc
H01-0564	EIF2AK3	NP_004827	NM_004836	COG1520/S_TKc
H01-0565	HOMER3	NP_004829	NM_004838	WH1
H01-0566	HOMER2	NP_004830	NM_004839	WH1
H01-0567	ARHGEF6	NP_004831	NM_004840	CH/RhoGEF/PH
H01-0568	RASAL2	NP_004832	NM_004841	C2/RasGAP
H01-0569	ROCK2	NP_004841	NM_004850	S_TKc/Smc/C1
H01-0570	STX8	NP_004844	NM_004853	MtPK/t_SNARE
H01-0571	AKAP5	NP_004848	NM_004857	
H01-0572	CLTC	NP_004850	NM_004859	Clathrin/CLH
H01-0573	GMFG	NP_004868	NM_004877	cofilin_ADF
H01-0574	SEC22L1	NP_004883	NM_004892	SNC1
H01-0575	PITPNM1	NP_004901	NM_004910	IP_trans/DDHD/SMP2
H01-0576	CALB1	NP_004920	NM_004929	FRQ1
H01-0577	CDK5	NP_004926	NM_004935	pkinase/S_TKc/TyrKc
H01-0578	DNM2	NP_004936	NM_004945	dynamin/PH/GED
H01-0579	MARK2	NP_004945	NM_004954	S_TKc/KA1
H01-0580	FRAP1	NP_004949	NM_004958	TEL1/FRB/PI3_PI4_kinase/FATC
H01-0581	GUCY2C	NP_004954	NM_004963	ANF_receptor/TyrKc/CYCc
H01-0582	JAK2	NP_004963	NM_004972	B41/SH2/TyrKc
H01-0583	KIF5A	NP_004975	NM_004984	kinesin/Smc
H01-0584	KTN1	NP_004977	NM_004986	
H01-0585	MYO1E	NP_004989	NM_004998	MYSc/SH3
H01-0586	MYO6	NP_004990	NM_004999	MYSc
H01-0587	ROR1	NP_005003	NM_005012	IG/Fz/KR/TyrKc
H01-0588	PDE1A	NP_005010	NM_005019	PDEase
H01-0589	PDE1C	NP_005011	NM_005020	PDEase
H01-0590	PFN1	NP_005013	NM_005022	PROF
H01-0591	PIK3CD	NP_005017	NM_005026	PI3K
H01-0592	PIP5K2A	NP_005019	NM_005028	PIP5K
H01-0593	PLK1	NP_005021	NM_005030	S_TKc/POLO_box
H01-0594	PLS3	NP_005023	NM_005032	SAC6
H01-0595	PRF1	NP_005032	NM_005041	MACPF/C2
H01-0596	RAC3	NP_005043	NM_005052	RHO
H01-0597	RANBP2L1	NP_005045	NM_005054	Ran_BP1/GRIP
H01-0598	RAPSN	NP_005046	NM_005055	COG5540
H01-0599	PLA2G4B	NP_005081	NM_005090	C2/PLA2_B
H01-0600	OSR1	NP_005100	NM_005109	S_TKc
H01-0601	PPP4R1	NP_005125	NM_005134	HEAT
H01-0602	ANXA3	NP_005130	NM_005139	annexin
H01-0603	USP10	NP_005144	NM_005153	UBP5
H01-0604	USP8	NP_005145	NM_005154	Rhodanese/UBP5
H01-0605	ADRBK2	NP_005151	NM_005160	RGS/S_TKc/PH
H01-0606	AKT1	NP_005154	NM_005163	PH/S_TKc
H01-0607	PPP2CZ	NP_005158	NM_005167	PP2Cc
H01-0608	ARHE	NP_005159	NM_005168	RHO

H01-0609	CALM3	NP_005175	NM_005184	FRQ1
H01-0610	CALML3	NP_005176	NM_005185	FRQ1
H01-0611	CAPN1	NP_005177	NM_005186	Calpain_III/FRQ1
H01-0612	CCNC	NP_005181	NM_005190	CCL1
H01-0613	CDKN3	NP_005183	NM_005192	CDC14
H01-0614	MAP3K8	NP_005195	NM_005204	S_TKc
H01-0615	CRK	NP_005197	NM_005206	SH2/SH3
H01-0616	CRKL	NP_005198	NM_005207	SH2/SH3
H01-0617	EPHA1	NP_005223	NM_005232	EPH_lbd/FN3/TyrKc/SAM
H01-0618	FER	NP_005237	NM_005246	FCH/SH2/TyrKc
H01-0619	FGR	NP_005239	NM_005248	SH3/SH2/TyrKc
H01-0620	GAK	NP_005246	NM_005255	S_TKc/Y_phosphatase/DnaJ
H01-0621	GEM	NP_005252	NM_005261	RAS
H01-0622	GNAT2	NP_005263	NM_005272	G-alpha
H01-0623	GRK4	NP_005298	NM_005307	RGS/S_TKc/GRK4
H01-0624	GRK5	NP_005299	NM_005308	RGS/S_TKc/GRK5
H01-0625	GRB7	NP_005301	NM_005310	RA/PH/SH2
H01-0626	GRB10	NP_005302	NM_005311	RA/PH/SH2
H01-0627	RAPGEF1	NP_005303	NM_005312	RasGEFN/RasGEF
H01-0628	HCLS1	NP_005326	NM_005335	HS1_rep/SH3
H01-0629	HIP1	NP_005329	NM_005338	Smc/ILWEQ
H01-0630	HRAS	NP_005334	NM_005343	RAS
H01-0631	KIF25	NP_005346	NM_005355	KISc
H01-0632	LCK	NP_005347	NM_005356	SH3/SH2/TyrKc
H01-0633	MCF2	NP_005360	NM_005369	SEC14/SPEC/RhoGEF/PH
H01-0634	RAB8A	NP_005361	NM_005370	ras
H01-0635	MOS	NP_005363	NM_005372	pkinase
H01-0636	MPP2	NP_005365	NM_005374	L27/PDZ/SH3/GuKc
H01-0637	MYO1A	NP_005370	NM_005379	MYSc
H01-0638	PLXNB3	NP_005384	NM_005393	Sema/PSI/TIG/RasGAP
H01-0639	PPP1R3C	NP_005389	NM_005398	CBM_21
H01-0640	PRKAB2	NP_005390	NM_005399	
H01-0641	PRKCE	NP_005391	NM_005400	C2/DAG_PE-bind/S_TKc
H01-0642	PTPN14	NP_005392	NM_005401	B41/PTPc
H01-0643	ROCK1	NP_005397	NM_005406	S_TKc/C1
H01-0644	SRC	NP_005408	NM_005417	SH3/SH2/TyrKc
H01-0645	TIE	NP_005415	NM_005424	EGF_Lam/fn3/fn3/fn3/TyrKc
H01-0646	TP53BP2	NP_005417	NM_005426	SbcC/ank/SH3
H01-0647	TP73	NP_005418	NM_005427	P53/SAM
H01-0648	VAV1	NP_005419	NM_005428	CH/RhoGEF/PH/DAG-bind/SH3/SH2
H01-0649	YES1	NP_005424	NM_005433	SH3/SH2/TyrKc
H01-0650	ARHGEF5	NP_005426	NM_005435	RhoGEF/SH3
H01-0651	ARHN	NP_005431	NM_005440	RHO
H01-0652	PAMCI	NP_005438	NM_005447	RA
H01-0653	PDLIM7	NP_005442	NM_005451	PDZ/LIM
H01-0654	GUCA1C	NP_005450	NM_005459	FRQ1
H01-0655	AKT3	NP_005456	NM_005465	PH/S_TKc
H01-0656	ABI1	NP_005461	NM_005470	SH3
H01-0657	LNK	NP_005466	NM_005475	PH/SH2
H01-0658	THRAP5	NP_005472	NM_005481	WD40
H01-0659	SH2D3C	NP_005480	NM_005489	SH2/RasGEF

H01-0660	SH2D3A	NP_005481	NM_005490	SH2
H01-0661	AP1M2	NP_005489	NM_005498	Adap_comp_sub
H01-0662	APBA2	NP_005494	NM_005503	PID/PDZ
H01-0663	CFL1	NP_005498	NM_005507	ADF
H01-0664	INPP5A	NP_005530	NM_005539	IPPc
H01-0665	INPP5D	NP_005532	NM_005541	SH2/IPPc
H01-0666	ITK	NP_005537	NM_005546	PH/BTK/SH3/SH2/TyrKc
H01-0667	KIFC3	NP_005541	NM_005550	kinesin
H01-0668	LCP2	NP_005556	NM_005565	SAM/SH2/SH2
H01-0669	MUSK	NP_005583	NM_005592	IG/IG/IGc2/Fz/TyrKc
H01-0670	PPP3CC	NP_005596	NM_005605	PP2Ac
H01-0671	PTK2	NP_005598	NM_005607	B41/TyrKc/Focal_AT
H01-0672	RGS4	NP_005604	NM_005613	RGS
H01-0673	RHEB	NP_005605	NM_005614	RAS
H01-0674	SDCBP	NP_005616	NM_005625	PDZ
H01-0675	SGK	NP_005618	NM_005627	S_TKc
H01-0676	SOS1	NP_005624	NM_005633	H2A/RhoGEF/PH/RasGEF
H01-0677	SYBL1	NP_005629	NM_005638	synaptobrevin
H01-0678	SYT1	NP_005630	NM_005639	C2/C2
H01-0679	EPM2A	NP_005661	NM_005670	CBM_20/DSPc
H01-0680	USH1C	NP_005700	NM_005709	PDZ
H01-0681	RGS19IP1	NP_005707	NM_005716	PDZ
H01-0682	ARPC5	NP_005708	NM_005717	
H01-0683	ARPC4	NP_005709	NM_005718	
H01-0684	ARPC3	NP_005710	NM_005719	P21-Arc
H01-0685	ARPC1B	NP_005711	NM_005720	COG2319
H01-0686	ACTR3	NP_005712	NM_005721	ACTIN
H01-0687	ACTR2	NP_005713	NM_005722	ACTIN
H01-0688	KIF20A	NP_005724	NM_005733	KISc/kinesin
H01-0689	HIPK3	NP_005725	NM_005734	S_TKc/S_TKc
H01-0690	ARL7	NP_005728	NM_005737	arf
H01-0691	ARL4A	NP_005729	NM_005738	arf
H01-0692	RASGRP1	NP_005730	NM_005739	RasGEF/DAG_PE-bind
H01-0693	G3BP	NP_005745	NM_005754	NTF2/RRM
H01-0694	ABI2	NP_005750	NM_005759	SH3
H01-0695	FARP1	NP_005757	NM_005766	Band_41/RhoGEF/PH
H01-0696	SCAM-1	NP_005766	NM_005775	Sorb/SH3
H01-0697	ACK1	NP_005772	NM_005781	TyrKc/SH3/DedD
H01-0698	PRKD3	NP_005804	NM_005813	DAG_PE-bind/PH/S_TKc
H01-0699	STX6	NP_005810	NM_005819	t_SNARE
H01-0700	STAM2	NP_005834	NM_005843	VHS/SH3
H01-0701	AKAP8	NP_005849	NM_005858	
H01-0702	NET1	NP_005854	NM_005863	RhoGEF
H01-0703	RGS19	NP_005864	NM_005873	RGS
H01-0704	PAK4	NP_005875	NM_005884	PBD/S_TKc
H01-0705	MAK	NP_005897	NM_005906	S_TKc
H01-0706	MAP3K5	NP_005914	NM_005923	S_TKc
H01-0707	MLLT4	NP_005927	NM_005936	RA/FHA/DIL/PDZ
H01-0708	PTK6	NP_005966	NM_005975	SH3/SH2/TyrKc
H01-0709	STK10	NP_005981	NM_005990	S_TKc/SbcC
H01-0710	UCHL3	NP_005993	NM_006002	Peptidase_C12

H01-0711	CPNE6	NP_006023	NM_006032	C2/VWA
H01-0712	CDC42BPB	NP_006026	NM_006035	S_TKc/DAG_PE-bind/CNH/PBD
H01-0713	UBE4B	NP_006039	NM_006048	UFD2
H01-0714	RRAGB	NP_006055	NM_006064	ras
H01-0715	HRBL	NP_006067	NM_006076	ArfGap
H01-0716	DLC1	NP_006085	NM_006094	SAM/RhoGAP/START
H01-0717	MYL9	NP_006088	NM_006097	FRQ1
H01-0718	RAPGEF3	NP_006096.2	NM_006105	DEP/cNMP_binding/RasGEF
H01-0719	VAV3	NP_006104.3	NM_006113	CH/RhoGEF/PH/DAG_PE-bind/SH2/SH3
H01-0720	MAP3K7IP1	NP_006107	NM_006116	PP2C
H01-0721	DYNC1LI2	NP_006132	NM_006141	RAD55
H01-0722	LASP1	NP_006139	NM_006148	LIM/NEBU/SH3
H01-0723	NCK1	NP_006144	NM_006153	SH3/SH2
H01-0724	NSF	NP_006169	NM_006178	cdc48_N/SpoVK
H01-0725	DDR2	NP_006173	NM_006182	F5_F8_type_C/TyrKc
H01-0726	PDE4A	NP_006193	NM_006202	PDEase
H01-0727	PDE4D	NP_006194	NM_006203	PDEase
H01-0728	PDE6C	NP_006195	NM_006204	GAF/GAF/PDEase
H01-0729	PHKG1	NP_006204	NM_006213	S_TKc
H01-0730	PIK3CA	NP_006209	NM_006218	PI3K
H01-0731	PIK3CB	NP_006210	NM_006219	PI3K
H01-0732	PITPNA	NP_006215	NM_006224	IP_trans
H01-0733	PLCD1	NP_006216	NM_006225	PH/PLC/C2
H01-0734	PLCL1	NP_006217	NM_006226	PLCXc/PLCYc/C2
H01-0735	PPEF2	NP_006230	NM_006239	PP2Ac/PP2Ac/FRQ1
H01-0736	PPEF1	NP_006231	NM_006240	PP2Ac/FRQ1
H01-0737	PPP1R3D	NP_006233	NM_006242	CBM_21
H01-0738	PPP2R5A	NP_006234	NM_006243	B56
H01-0739	PPP2R5B	NP_006235	NM_006244	B56
H01-0740	PPP2R5D	NP_006236	NM_006245	B56
H01-0741	PPP2R5E	NP_006237	NM_006246	B56
H01-0742	PPP5C	NP_006238	NM_006247	NrfG/PP2Ac
H01-0743	PRKAA1	NP_006242	NM_006251	S_TKc
H01-0744	PRKAA2	NP_006243	NM_006252	S_TKc
H01-0745	PRKCD	NP_006245	NM_006254	DAG_PE-bind/DAG_PE-bind/S_TKc
H01-0746	PRKCH	NP_006246	NM_006255	C2/DAG_PE-bind/S_TKc
H01-0747	PKN2	NP_006247	NM_006256	HR1/S_TKc
H01-0748	PRKCQ	NP_006248	NM_006257	DAG_PE-bind/S_TKc
H01-0749	PRKG1	NP_006249	NM_006258	cNMP/S_TKc
H01-0750	PRKG2	NP_006250	NM_006259	S_TKc
H01-0751	RALGDS	NP_006257	NM_006266	RasGEFN/RasGEF/RA
H01-0752	RANBP2	NP_006258	NM_006267	Nrf/Ran_BP1/zf-RanBP
H01-0753	RRAS	NP_006261	NM_006270	RAS
H01-0754	STK3	NP_006272	NM_006281	S_TKc
H01-0755	STK4	NP_006273	NM_006282	S_TKc
H01-0756	TESK1	NP_006276	NM_006285	pkinase
H01-0757	TLN1	NP_006280	NM_006289	Band_41/ILWEQ
H01-0758	TSG101	NP_006283	NM_006292	UBCc/Smc
H01-0759	TYRO3	NP_006284	NM_006293	IG/fn3/fn3/TyrKc
H01-0760	VRK2	NP_006287	NM_006296	pkinase
H01-0761	MAP3K12	NP_006292	NM_006301	pkinase

H01-0762	USP15	NP_006304	NM_006313	UBP12/UBP12
H01-0763	CNKSR1	NP_006305	NM_006314	SAM/PH
H01-0764	MERTK	NP_006334	NM_006343	IG/IFN3/TyrKc
H01-0765	CAP2	NP_006357	NM_006366	CAP
H01-0766	VTI1B	NP_006361	NM_006370	t_SNARE
H01-0767	STK25	NP_006365	NM_006374	S_TKc
H01-0768	UNC13B	NP_006368.2	NM_006377	C2/DAG_PE-bind/C2/C2
H01-0769	CIB2	NP_006374	NM_006383	FRQ1
H01-0770	IPO8	NP_006381	NM_006390	SXM1
H01-0771	IPO7	NP_006382	NM_006391	SXM1
H01-0772	DCTN2	NP_006391	NM_006400	
H01-0773	NEDD9	NP_006394	NM_006403	SH3
H01-0774	ARPC1A	NP_006400	NM_006409	COG2319
H01-0775	ARFGEF2	NP_006411	NM_006420	COG5307
H01-0776	ARFGEF1	NP_006412	NM_006421	COG5307
H01-0777	RABAC1	NP_006414	NM_006423	PRA1
H01-0778	USP16	NP_006438	NM_006447	UBP12/UBP12
H01-0779	CDC42EP3	NP_006440	NM_006449	PBD
H01-0780	PDLIM5	NP_006448	NM_006457	PDZ/LIM
H01-0781	STAMBP	NP_006454	NM_006463	Mov34
H01-0782	RRP22	NP_006468	NM_006477	RAS
H01-0783	RGS14	NP_006471	NM_006480	RGS/RBD
H01-0784	DYRK2	NP_006473	NM_006482	S_TKc
H01-0785	GNAI3	NP_006487	NM_006496	G-alpha
H01-0786	RASA2	NP_006497	NM_006506	C2/RasGAP/BTK
H01-0787	USP3	NP_006528	NM_006537	UBP14/UBP12
H01-0788	YKT6	NP_006546	NM_006555	SNC1
H01-0789	RRAGA	NP_006561	NM_006570	COG1100
H01-0790	GNA13	NP_006563	NM_006572	G-alpha
H01-0791	MAP4K5	NP_006566	NM_006575	S_TKc/CNH
H01-0792	AVIL	NP_006567	NM_006576	GEL/VHP
H01-0793	AP4B1	NP_006585	NM_006594	Adaptin_N
H01-0794	KIF1C	NP_006603	NM_006612	KISc/FHA
H01-0795	GRAP	NP_006604	NM_006613	SH3/SH2
H01-0796	PLK2	NP_006613	NM_006622	S_TKc/POLO_box/POLO_box
H01-0797	IQGAP2	NP_006624	NM_006633	IQG1/IQ/RasGAP
H01-0798	WASF3	NP_006637	NM_006646	WH2
H01-0799	FRS2	NP_006645	NM_006654	IRS
H01-0800	PDE10A	NP_006652	NM_006661	FhlA/GAF/PDEase
H01-0801	RAI	NP_006654	NM_006663	SH3
H01-0802	USP20	NP_006667	NM_006676	UBP12/UBP12/DUSP/DUSP
H01-0803	RPIP8	NP_006686	NM_006695	RUN
H01-0804	NTE	NP_006693	NM_006702	cNMP_binding/RssA
H01-0805	ABLIM1	NP_006711	NM_006720	VHP
H01-0806	MAP3K4	NP_006715	NM_006724	S_TKc
H01-0807	PSKH1	NP_006733	NM_006742	S_TKc
H01-0808	SLA	NP_006739	NM_006748	SH3/SH2
H01-0809	YWHAE	NP_006752	NM_006761	14-3-3
H01-0810	CDC42EP2	NP_006770	NM_006779	PBD
H01-0811	WDR3	NP_006775	NM_006784	WD40
H01-0812	RALBP1	NP_006779	NM_006788	RhoGAP

H01-0813	AP3M2	NP_006794	NM_006803	Adap_comp_sub
H01-0814	RAB40B	NP_006813	NM_006822	RAB/SOCS
H01-0815	YWHAQ	NP_006817	NM_006826	14-3-3
H01-0816	PLEKHC1	NP_006823	NM_006832	B41/PH/B41
H01-0817	RAB32	NP_006825	NM_006834	RAB
H01-0818	CCNI	NP_006826	NM_006835	cyclin
H01-0819	KIF2C	NP_006836	NM_006845	kinesin
H01-0820	RABL4	NP_006851	NM_006860	ras
H01-0821	RAB35	NP_006852	NM_006861	RAB
H01-0822	RAB31	NP_006859	NM_006868	RAS/arf/ARF/RAB/ras/RHO/RAN
H01-0823	CENTA1	NP_006860	NM_006869	ArfGap/PH
H01-0824	DSTN	NP_006861	NM_006870	cofilin_ADF
H01-0825	RIPK3	NP_006862	NM_006871	pkinase
H01-0826	PIM2	NP_006866	NM_006875	S_TKc
H01-0827	CALM1	NP_008819	NM_006888	FRQ1
H01-0828	MYO9A	NP_008832	NM_006901	RA/MYSc/DAG_PE-bind/RhoGAP
H01-0829	RIT1	NP_008843	NM_006912	RAS
H01-0830	SPTBN2	NP_008877	NM_006946	SAC6/SPEC/PH
H01-0831	SYN1	NP_008881	NM_006950	Synapsin_C
H01-0832	RPH3AL	NP_008918	NM_006987	RPH3A_effector
H01-0833	WASF2	NP_008921	NM_006990	
H01-0834	WWP2	NP_008945	NM_007014	C2/PRP40/WW/HECTc
H01-0835	CEP1	NP_008949	NM_007018	Smc
H01-0836	UBE2C	NP_008950	NM_007019	UBCc
H01-0837	RAPGEF4	NP_008954	NM_007023	cNMP_binding/DEP//RasGEF
H01-0838	DUSP14	NP_008957	NM_007026	DSPc
H01-0839	HRIHFB2122	NP_008963	NM_007032	PH/Smc
H01-0840	PTPN21	NP_008970	NM_007039	B41/PTPc
H01-0841	KIF3A	NP_008985	NM_007054	kinesin
H01-0842	TBC1D8	NP_008994	NM_007063	GRAM/COG5210
H01-0843	TRAD	NP_008995	NM_007064	RhoGEF/PH/IG/FN3/S_TKc
H01-0844	CALB2	NP_009019	NM_007088	FRQ1
H01-0845	CLTCL1	NP_009029	NM_007098	Clathrin/CLH
H01-0846	TRIO	NP_009049	NM_007118	SEC14/SPEC/SH3/RhoGEF/PH/IG/S_TKc
H01-0847	VCP	NP_009057	NM_007126	cdc48_N/SpoVK
H01-0848	VIL1	NP_009058	NM_007127	GEL/VHP
H01-0849	PICALM	NP_009097	NM_007166	ENTH
H01-0850	TESK2	NP_009101	NM_007170	pkinase
H01-0851	NUP50	NP_009103	NM_007172	RanBD
H01-0852	MAP4K1	NP_009112	NM_007181	S_TKc/CNH
H01-0853	SEC23IP	NP_009121	NM_007190	SAM/DDHD
H01-0854	ANXA10	NP_009124	NM_007193	annexin
H01-0855	IRAK3	NP_009130	NM_007199	death/pkinase
H01-0856	AKAP10	NP_009133	NM_007202	RGS/RGS/RGS
H01-0857	DUSP10	NP_009138	NM_007207	RHOD/DSPc
H01-0858	C12orf2	NP_009142	NM_007211	RA
H01-0859	CHP	NP_009167	NM_007236	FRQ1
H01-0860	DUSP12	NP_009171	NM_007240	DSPc
H01-0861	STXBP3	NP_009200	NM_007269	Sec1
H01-0862	STK38	NP_009202	NM_007271	S_TKc/S_TK_X
H01-0863	PTK9L	NP_009215	NM_007284	ADF/ADF

H01-0864	ABL1	NP_009297	NM_007313	SH3/SH2/TyrKc
H01-0865	KIF22	NP_015556	NM_007317	KISc/ComEA
H01-0866	RANBP3	NP_015560	NM_007321	
H01-0867	ZFYVE9	NP_015563	NM_007324	FYVE
H01-0868	AP4E1	NP_031373	NM_007347	Adaptin_N
H01-0869	GNA12	NP_031379	NM_007353	G-alpha
H01-0870	RASA3	NP_031394	NM_007368	C2/RasGAP/BTK
H01-0871	DNM1L	NP_036193	NM_012063	dynamin_2
H01-0872	AP3M1	NP_036227	NM_012095	Adap_comp_sub
H01-0873	APPL	NP_036228	NM_012096	COG2905/PH/PTB
H01-0874	BRDG1	NP_036240	NM_012108	SH2
H01-0875	CCRK	NP_036251	NM_012119	S_TKc
H01-0876	CD2AP	NP_036252	NM_012120	SH3
H01-0877	CDC42EP4	NP_036253	NM_012121	
H01-0878	FKBP8	NP_036313	NM_012181	FKBP/TPR
H01-0879	RABGAP1	NP_036329	NM_012197	PTB/TBC/Smc
H01-0880	GCA	NP_036330	NM_012198	EF-hand/FRQ1
H01-0881	MRAS	NP_036351	NM_012219	RAS
H01-0882	RHOQ	NP_036381	NM_012249	RHO
H01-0883	RRAS2	NP_036382	NM_012250	RAS
H01-0884	PLD3	NP_036400	NM_012268	Cls
H01-0885	CENTB2	NP_036419	NM_012287	PH/ArfGap/Arp
H01-0886	TLK1	NP_036422	NM_012290	S_TKc
H01-0887	RAPGEF5	NP_036426	NM_012294	RasGEFN/RasGEF
H01-0888	CABIN1	NP_036427	NM_012295	
H01-0889	G3BP2	NP_036429	NM_012297	NTF2/RRM
H01-0890	AIP1	NP_036433	NM_012301	PDZ/GuKc/WW
H01-0891	AP2A2	NP_036437	NM_012305	Adaptin_N/Alpha_adaptinC
H01-0892	EPB41L3	NP_036439	NM_012307	B41/Band_41/B41/ERM
H01-0893	SHANK2	NP_036441	NM_012309	PDZ/SAM
H01-0894	KIF4A	NP_036442	NM_012310	KISc/Smc
H01-0895	MYO10	NP_036466	NM_012334	MYSc/PH/MyTH4/B41
H01-0896	MYO1F	NP_036467	NM_012335	MYSc/SH3
H01-0897	OSTF1	NP_036515	NM_012383	SH3
H01-0898	PEF1	NP_036524	NM_012392	FRQ1
H01-0899	PFTK1	NP_036527	NM_012395	S_TKc
H01-0900	PITPNB	NP_036531	NM_012399	IP_trans
H01-0901	PLXNB2	NP_036533	NM_012401	RasGAP
H01-0902	PRKCABP	NP_036539	NM_012407	PDZ
H01-0903	PTPN22	NP_036543	NM_012411	PTPc
H01-0904	RAB3GAP2	NP_036546	NM_012414	
H01-0905	PITPNC1	NP_036549	NM_012417	IP_trans
H01-0906	RGS17	NP_036551	NM_012419	RGS
H01-0907	RPS6KC1	NP_036556	NM_012424	PX/MIT/S_TKc/S_TKc
H01-0908	SEC22L2	NP_036562	NM_012430	synaptobrevin
H01-0909	STAT5B	NP_036580	NM_012448	STAT/STAT_bind/SH2
H01-0910	TBL2	NP_036585	NM_012453	COG2319/WD40
H01-0911	TIAM2	NP_036586	NM_012454	RBD/PDZ/RhoGEF
H01-0912	PSD4	NP_036587	NM_012455	Sec7/PH
H01-0913	STK39	NP_037365	NM_013233	S_TKc
H01-0914	PR48	NP_037371	NM_013239	

H01-0915	TBK1	NP_037386	NM_013254	S_TKc
H01-0916	MYLIP	NP_037394	NM_013262	Band_41/COG5236
H01-0917	CTNNA3	NP_037398	NM_013266	Vinculin
H01-0918	RACGAP1	NP_037409	NM_013277	Smc/DAG_PE-bind/RhoGAP
H01-0919	HUMMLC2B	NP_037424	NM_013292	FRQ1
H01-0920	EEF2K	NP_037434	NM_013302	MHCK_EF2_kinase/COG0790
H01-0921	BLNK	NP_037446	NM_013314	Occludin/SH2
H01-0922	TPTE	NP_037447	NM_013315	CDC14
H01-0923	EPN1	NP_037465	NM_013333	ENTH
H01-0924	PKN3	NP_037487	NM_013355	HR1/HR1/S_TKc
H01-0925	GGA1	NP_037497	NM_013365	VHS/GAT/Alpha_adaptinC2
H01-0926	PSCD4	NP_037517	NM_013385	Sec7/PH
H01-0927	NRBP	NP_037524	NM_013392	S_TKc
H01-0928	USP25	NP_037528	NM_013396	COG5077/UCH-2
H01-0929	RABL2A	NP_038198	NM_013412	RAB
H01-0930	NCF4	NP_038202	NM_013416	PX/SH3
H01-0931	ARHGAP6	NP_038286	NM_013427	RhoGAP
H01-0932	NOX1	NP_039249	NM_013955	Ferric_reduct
H01-0933	VCL	NP_054706	NM_014000	Vinculin
H01-0934	IKBKE	NP_054721	NM_014002	S_TKc
H01-0935	REM1	NP_054731	NM_014012	RAS
H01-0936	SACM1L	NP_054735	NM_014016	Syja_N
H01-0937	GIT1	NP_054749	NM_014030	ArfGap/GIT
H01-0938	DBNL	NP_054782	NM_014063	ADF/SH3
H01-0939	HIPK2	NP_054794	NM_022740	S_TKc
H01-0940	DUOX2	NP_054799	NM_014080	FRQ1/Ferric_reduct
H01-0941	STXBP6	NP_054897	NM_014178	
H01-0942	ITPK1	NP_055031	NM_014216	RimK
H01-0943	RAGE	NP_055041	NM_014226	S_TKc
H01-0944	SLC25A13	NP_055066	NM_014251	FRQ1/mito_carr
H01-0945	PLK4	NP_055079	NM_014264	S_TKc
H01-0946	FREQ	NP_055101	NM_014286	FRQ1/EFh
H01-0947	CAPN6	NP_055104	NM_014289	CysPc/Calpain_III/C2
H01-0948	PIK3R5	NP_055123	NM_014308	
H01-0949	RASD2	NP_055125	NM_014310	RAS
H01-0950	RUSC1	NP_055143	NM_014328	RUN/SH3
H01-0951	PPP1R15A	NP_055145	NM_014330	
H01-0952	RAB26	NP_055168	NM_014353	RAB
H01-0953	PTPN18	NP_055184	NM_014369	PTPc
H01-0954	STK23	NP_055185	NM_014370	S_TKc/S_TKc
H01-0955	LAT	NP_055202	NM_014387	
H01-0956	NEK6	NP_055212	NM_014397	S_TKc
H01-0957	HRI	NP_055228	NM_014413	S_TKc/pkinase
H01-0958	PIB5PA	NP_055237	NM_014422	IPPc
H01-0959	TJP3	NP_055243	NM_014428	PDZ/GuKc
H01-0960	ARHGEF16	NP_055263	NM_014448	RhoGEF
H01-0961	SIT1	NP_055265.1	NM_014450	
H01-0962	RND1	NP_055285	NM_014470	RHO
H01-0963	PDLIM3	NP_055291	NM_014476	PDZ/LIM
H01-0964	RPS6KA6	NP_055311	NM_014496	S_TKc/S_TKc
H01-0965	SH3BP4	NP_055336	NM_014521	SH3

H01-0966	BCAR1	NP_055382	NM_014567	SH3
H01-0967	ARFGAP3	NP_055385	NM_014570	ArfGap
H01-0968	LATS2	NP_055387	NM_014572	S_TKc
H01-0969	RHOD	NP_055393	NM_014578	RHO
H01-0970	HUNK	NP_055401	NM_014586	S_TKc
H01-0971	HERC3	NP_055421	NM_014606	HECTc
H01-0972	ARHGEF10	NP_055444	NM_014629	RhoGEF
H01-0973	SH3MD1	NP_055446	NM_014631	SH3
H01-0974	MICAL2	NP_055447	NM_014632	UbiH/SAC6/LIM
H01-0975	PPM1F	NP_055449	NM_014634	PP2Cc
H01-0976	RALGPS1	NP_055451	NM_014636	RasGEF/PH
H01-0977	IQCB1	NP_055457	NM_014642	IQ
H01-0978	PDE4DIP	NP_055459	NM_014644	Myosin_tail/ERM
H01-0979	ENTH	NP_055481	NM_014666	ENTH
H01-0980	RIMS2	NP_055492	NM_014677	PDZ/C2
H01-0981	ULK2	NP_055498	NM_014683	S_TKc
H01-0982	RAB11FIP3	NP_055515	NM_014700	FRQ1/Smc
H01-0983	DOCK4	NP_055520	NM_014705	SH3
H01-0984	CENTB1	NP_055531	NM_014716	PH/ArfGap/Arp
H01-0985	SLK	NP_055535	NM_014720	S_TKc
H01-0986	SNPH	NP_055538	NM_014723	COG3883
H01-0987	STARD8	NP_055540	NM_014725	RhoGAP/START
H01-0988	ZFYVE16	NP_055548	NM_014733	FYVE
H01-0989	RIMS3	NP_055562	NM_014747	C2
H01-0990	CENTG1	NP_055585	NM_014770	ras/PH/PH/ArfGap
H01-0991	GIT2	NP_055591	NM_014776	ArfGap/GIT
H01-0992	ARHGAP11A	NP_055598	NM_014783	RhoGAP
H01-0993	ARHGEF11	NP_055599	NM_014784	PDZ/RGS/RhoGEF
H01-0994	ARHGEF17	NP_055601	NM_014786	RhoGEF
H01-0995	MELK	NP_055606	NM_014791	S_TKc/KA1
H01-0996	KIAA0528	NP_055617	NM_014802	C2
H01-0997	FARP2	NP_055623	NM_014808	Band_41/ERM/RhoGEF/PH/PH
H01-0998	KIAA0317	NP_055636	NM_014821	Filamin/HECTc
H01-0999	FCHSD2	NP_055639	NM_014824	SH3
H01-1000	TBC1D4	NP_055647	NM_014832	PTB/TBC
H01-1001	RHOBTB1	NP_055651	NM_014836	RHO/BTB
H01-1002	ARK5	NP_055655	NM_014840	S_TKc
H01-1003	SNAP91	NP_055656	NM_014841	ENTH
H01-1004	KIAA0274	NP_055660	NM_014845	Syja_N
H01-1005	SRGAP3	NP_055665	NM_014850	FCH/RhoGAP/SH3
H01-1006	KIAA0672	NP_055674	NM_014859	BAR/RhoGAP
H01-1007	IQSEC1	NP_055684	NM_014869	Sec7/PH
H01-1008	KIF14	NP_055690	NM_014875	KISc/FHA/ERM
H01-1009	ARHGAP25	NP_055697	NM_014882	PH/RhoGAP/COG4694
H01-1010	RHOBTB3	NP_055714	NM_014899	ras/BTB
H01-1011	DLGAP4	NP_055717	NM_014902	GKAP
H01-1012	RAB11FIP2	NP_055719	NM_014904	C2
H01-1013	PPM1E	NP_055721	NM_014906	PP2Cc
H01-1014	FRMPD1	NP_055722	NM_014907	PDZ/B41
H01-1015	AAK1	NP_055726	NM_014911	S_TKc
H01-1016	CENTG2	NP_055729	NM_014914	ras/PH/PH/ArfGap/ank

H01-1017	LMTK2	NP_055731	NM_014916	TyrKc
H01-1018	CNKSR2	NP_055742	NM_014927	SAM/PDZ/PH
H01-1019	PLEKHA6	NP_055750	NM_014935	PH
H01-1020	INPP5F	NP_055752	NM_014937	COG5329
H01-1021	RPH3A	NP_055769	NM_014954	RPH3A_effector/C2
H01-1022	RIPX	NP_055776	NM_014961	RUN/Smc
H01-1023	KIFAP3	NP_055785	NM_014970	
H01-1024	RIMS1	NP_055804	NM_014989	RPH3A_effector/PDZ/C2
H01-1025	WDFY3	NP_055806	NM_014991	Beach/COG2319/FYVE
H01-1026	RAB21	NP_055814	NM_014999	ras
H01-1027	STK38L	NP_055815	NM_015000	S_TKc
H01-1028	PDZD2	NP_835260	NM_178140	PDZ
H01-1029	NEDL1	NP_055867	NM_015052	C2/HECTc
H01-1030	ARHGAP26	NP_055886	NM_015071	BAR/PH/RhoGAP/SH3
H01-1031	KIF1B	NP_055889	NM_015074	KISc/FHA/PH
H01-1032	SARM1	NP_055892	NM_015077	SAM/SAM/TIR
H01-1033	MCF2L2	NP_055893	NM_015078	SEC14/RhoGEF/PH
H01-1034	SMG1	NP_055907	NM_015092	FAT/PI3_PI4_kinase/FATC
H01-1035	MAST2	NP_055927	NM_015112	S_TKc/PDZ
H01-1036	RGL1	NP_055964	NM_015149	RasGEFN/RasGEF/RA
H01-1037	PMPCA	NP_055975	NM_015160	PqqL
H01-1038	SYNE2	NP_055995	NM_015180	SAC6/Smc/SbcC/SPEC
H01-1039	PLCL2	NP_055999	NM_015184	PH/PLCXc/PLCYc/C2
H01-1040	ARHGEF9	NP_056000	NM_015185	SH3/RhoGEF
H01-1041	PLCB1	NP_056007	NM_015192	PI-PLC-X/PI-PLC-Y/C2
H01-1042	EHBP1	NP_056067	NM_015252	SAC6
H01-1043	KIF13B	NP_056069	NM_015254	KISc/SbcC/CAP_GLY
H01-1044	NEDD4L	NP_056092	NM_015277	C2/WW/HUL4
H01-1045	SASH1	NP_056093	NM_015278	SAM
H01-1046	MBC2	NP_056107	NM_015292	COG5038/C2
H01-1047	DOCK9	NP_056111	NM_015296	PH
H01-1048	PSD3	NP_056125	NM_015310	Sec7/PH
H01-1049	ARHGEF12	NP_056128	NM_015313	PDZ/RhoGEF
H01-1050	PPP1R13B	NP_056131	NM_015316	PRP38/SbcC/Arp/SH3
H01-1051	ARHGEF18	NP_056133	NM_015318	ROM1/Dbl/RhoGEF/PH
H01-1052	SCRIB	NP_056171	NM_015356	COG4886/PDZ
H01-1053	SORBS1	NP_056200	NM_015385	Sorb/SH3
H01-1054	PTPN23	NP_056281	NM_015466	BRO1/Smc/PTPc
H01-1055	RAB11FIP5	NP_056285	NM_015470	C2
H01-1056	SAMHD1	NP_056289	NM_015474	SAM/COG1078
H01-1057	SEC31L2	NP_056305	NM_015490	COG2319
H01-1058	SH2B	NP_056318	NM_015503	PH/SH2
H01-1059	SIPA1L1	NP_056371	NM_015556	Rap_GAP/PDZ
H01-1060	PPP1R16B	NP_056383	NM_015568	Arp
H01-1061	DNM3	NP_056384	NM_015569	dynamin/PH/GED
H01-1062	RAPGEF2	NP_056407	NM_014247	cNMP/RasGEFN/PDZ/RA
H01-1063	SGEF	NP_056410	NM_015595	RhoGEF/SH3
H01-1064	RAP1B	NP_056461	NM_015646	RAS
H01-1065	SH3YL1	NP_056492	NM_015677	COG2930/SH3
H01-1066	SDCBP2	NP_056500	NM_015685	PDZ
H01-1067	STK36	NP_056505	NM_015690	S_TKc

H01-1068	RUTBC3	NP_056520	NM_015705	TBC/SH3/RUN
H01-1069	LMO7	NP_056668	NM_015843	PDZ
H01-1070	VILL	NP_056957	NM_015873	GEL/VHP
H01-1071	EDD	NP_056986	NM_015902	ZnF_UBR1/PolyA/HECTc
H01-1072	TNNI3K	NP_057062	NM_015978	Arp/TyrKc
H01-1073	UBE2D4	NP_057067	NM_015983	COG5078
H01-1074	SH3GLB1	NP_057093	NM_016009	BAR/SH3
H01-1075	RASD1	NP_057168	NM_016084	RAS
H01-1076	MK-STYX	NP_057170	NM_016086	RHOD/DSPc
H01-1077	SAR1B	NP_057187	NM_016103	SAR
H01-1078	ASB1	NP_057198	NM_016114	Arp/ANK/SOCS
H01-1079	RAB10	NP_057215	NM_016131	ras
H01-1080	DNCLI1	NP_057225	NM_016141	COG4178
H01-1081	SHANK1	NP_057232	NM_016148	Arp/SH3/PDZ/SAM
H01-1082	ASB2	NP_057234	NM_016150	Arp/ank/SOCS
H01-1083	TAOK2	NP_057235	NM_016151	S_TKc
H01-1084	RAB4B	NP_057238	NM_016154	RAB
H01-1085	CRNN	NP_057274.1	NM_016190	S_100
H01-1086	PRKAG2	NP_057287	NM_016203	COG2524
H01-1087	PACSIN3	NP_057307	NM_016223	FCH/SH3
H01-1088	SNX9	NP_057308	NM_016224	SH3/PX
H01-1089	NLK	NP_057315	NM_016231	S_TKc
H01-1090	MYO15A	NP_057323	NM_016239	MYSc/MyTH4/SH3/B41
H01-1091	HPCAL4	NP_057341	NM_016257	FRQ1
H01-1092	PLEKHO1	NP_057358	NM_016274	PH
H01-1093	RAB23	NP_057361	NM_016277	ras
H01-1094	TAOK3	NP_057365	NM_016281	S_TKc
H01-1095	IHPK2	NP_057375	NM_016291	IPK
H01-1096	RAB14	NP_057406	NM_016322	RAB
H01-1097	SFMBT1	NP_057413	NM_016329	MBT/SAM
H01-1098	EVL	NP_057421	NM_016337	WH1
H01-1099	IPO11	NP_057422	NM_016338	CSE1
H01-1100	RAPGEFL1	NP_057423	NM_016339	RasGEF
H01-1101	RAPGEF6	NP_057424	NM_016340	PDZ/RA/RasGEF
H01-1102	PLCE1	NP_057425	NM_016341	RasGEF/PLC/C2/RA
H01-1103	NIN	NP_057434	NM_016350	Smc
H01-1104	DUSP13	NP_057448	NM_016364	DSPc
H01-1105	CABP5	NP_057451	NM_016367	FRQ1
H01-1106	RAB9B	NP_057454	NM_016370	ras
H01-1107	ANKFY1	NP_057460	NM_016376	BTB/Arp/ank/FYVE
H01-1108	AKAP7	NP_057461	NM_016377	LigT
H01-1109	TCRIM	NP_057472	NM_016388	
H01-1110	VRK3	NP_057524	NM_016440	pkinase
H01-1111	PLEK2	NP_057529	NM_016445	PH/DEP/PH
H01-1112	COPB1	NP_057535	NM_016451	Adaptin_N
H01-1113	NCKIPSD	NP_057537	NM_016453	SH3
H01-1114	PRKD2	NP_057541	NM_016457	DAG_PE-bind/S_TKc
H01-1115	PDZK11	NP_057568	NM_016484	PDZ
H01-1116	RANGNRF	NP_057576	NM_016492	
H01-1117	CRKRS	NP_057591	NM_016507	S_TKc
H01-1118	CDKL3	NP_057592	NM_016508	S_TKc

H01-1119	ICK	NP_057597	NM_016513	S_TKc
H01-1120	UBAP1	NP_057609	NM_016525	
H01-1121	RAB8B	NP_057614	NM_016530	ras
H01-1122	SKIP	NP_057616	NM_016532	IPPc
H01-1123	MST4	NP_057626	NM_016542	S_TKc
H01-1124	RBJ	NP_057628	NM_016544	RAB/DnaJ
H01-1125	ANKMY1	NP_057636	NM_016552	ank/Arp
H01-1126	RASL12	NP_057647	NM_016563	RAS
H01-1127	USP21	NP_057656	NM_016572	UBP5
H01-1128	GMIP	NP_057657	NM_016573	FCH/C1/RhoGAP
H01-1129	RAB6B	NP_057661	NM_016577	ras
H01-1130	C5orf5	NP_057687	NM_016603	RhoGAP
H01-1131	SPTBN5	NP_057726	NM_016642	SAC6/SPEC/PH
H01-1132	LIMK2	NP_057952	NM_016733	LIM//Dlg/PDZ/TyrKc
H01-1133	LIMK1	NP_058015	NM_016735	LIM/PDZ
H01-1134	MAPT	NP_058525	NM_016841	tubulin-binding
H01-1135	SHC3	NP_058544	NM_016848	PID/SH2
H01-1136	STX18	NP_058626	NM_016930	
H01-1137	PARD6A	NP_058644	NM_016948	PB1/PDZ
H01-1138	PDE11A	NP_058649	NM_016953	GAF/PDEase
H01-1139	USP18	NP_059110	NM_017414	COG5077
H01-1140	CALML5	NP_059118	NM_017422	FRQ1
H01-1141	PRKAG3	NP_059127	NM_017431	COG2524
H01-1142	MYO3A	NP_059129	NM_017433	S_TKc/MYSc
H01-1143	BAIAP2	NP_059344	NM_017450	SbcC/SH3
H01-1144	PSCD1	NP_059430	NM_017456	Sec7/PH
H01-1145	PSCD2	NP_059431	NM_017457	Sec7/PH
H01-1146	WDR1	NP_059830	NM_017491	WD40
H01-1147	MKNK2	NP_060042	NM_017572	S_TKc
H01-1148	BMP2K	NP_060063	NM_017593	S_TKc
H01-1149	DIRAS2	NP_060064	NM_017594	RAS
H01-1150	NKIRAS2	NP_060065	NM_017595	ras
H01-1151	PPP1R9A	NP_060120	NM_017650	SAM
H01-1152	AHI1	NP_060121	NM_017651	SH3
H01-1153	GIPC2	NP_060125	NM_017655	PDZ
H01-1154	OTUB1	NP_060140	NM_017670	COG5539
H01-1155	C20orf42	NP_060141	NM_017671	B41/PH
H01-1156	MTMR8	NP_060147	NM_017677	PTPc_motif
H01-1157	FLJ20184	NP_060170	NM_017700	RhoGEF
H01-1158	ARHGAP8	NP_060171	NM_017701	
H01-1159	DDEFL1	NP_060177	NM_017707	PH/ArfGap/Arp
H01-1160	SNRK	NP_060189	NM_017719	S_TKc
H01-1161	STAP2	NP_060190	NM_017720	SH2
H01-1162	FLJ20241	NP_060191	NM_017721	DM14/C2
H01-1163	PPP1R14D	NP_060196	NM_017726	
H01-1164	OSBPL7	NP_060201	NM_017731	PH/Oxysterol_BP
H01-1165	FNBP1L	NP_060207	NM_017737	SH3
H01-1166	PRG-3	NP_060223	NM_017753	PAP2
H01-1167	PXK	NP_060241	NM_017771	PX/S_TKc
H01-1168	OSBPL10	NP_060254	NM_017784	PH/Oxysterol_BP
H01-1169	UBE2R2	NP_060281	NM_017811	COG5078

H01-1170	RAB20	NP_060287	NM_017817	ras
H01-1171	DUSP23	NP_060293	NM_017823	CDC14
H01-1172	AYTL1	NP_060309	NM_017839	PlsC/FRQ1
H01-1173	SSH3	NP_060327	NM_017857	DSPc
H01-1174	FLJ20574	NP_060356	NM_017886	S_TKc
H01-1175	TESC	NP_060369	NM_017899	FRQ1
H01-1176	STX17	NP_060389	NM_017919	t_SNARE
H01-1177	FLJ20701	NP_060403	NM_017933	PTB
H01-1178	CADPS2	NP_060424	NM_017954	PH
H01-1179	EPN3	NP_060427	NM_017957	ENTH
H01-1180	PLEKHB2	NP_060428	NM_017958	PH
H01-1181	RUFY2	NP_060457	NM_017987	RUN/FYVE
H01-1182	SCYL2	NP_060458	NM_017988	S_TKc/TyrKc
H01-1183	FRMD4A	NP_060497	NM_018027	B41
H01-1184	RALGPS2	NP_060507	NM_018037	RasGEF
H01-1185	PLEKHJ1	NP_060519	NM_018049	PH
H01-1186	ARHGAP17	NP_060524	NM_018054	BAR/RhoGAP
H01-1187	FLJ10324	NP_060529	NM_018059	DIL/PDZ
H01-1188	FLJ10357	NP_060541	NM_018071	PH
H01-1189	ECT2	NP_060568	NM_018098	BRCT/BRCT/RhoGEF
H01-1190	EFHC1	NP_060570	NM_018100	DM10/FRQ1
H01-1191	DOK4	NP_060580	NM_018110	PH/PTBI
H01-1192	DIP13B	NP_060641	NM_018171	PH/PTB
H01-1193	FLJ10665	NP_060643	NM_018173	RhoGEF
H01-1194	ARL8B	NP_060654	NM_018184	arf
H01-1195	UBE1L2	NP_060697	NM_018227	ThiF/UBACT
H01-1196	C14orf108	NP_060699	NM_018229	Adap_comp_sub
H01-1197	MULK	NP_060708	NM_018238	LCB5
H01-1198	KIF24	NP_060748	NM_018278	
H01-1199	ARHGAP12	NP_060757	NM_018287	SH3/WW/PH/RhoGAP
H01-1200	RHOT1	NP_060777	NM_018307	RHO/RAB
H01-1201	UEV3	NP_060784	NM_018314	UBCc/Mdh/ldh_C
H01-1202	PI4K2B	NP_060793	NM_018323	
H01-1203	MCTP2	NP_060819	NM_018349	C2
H01-1204	FGD6	NP_060821	NM_018351	RhoGEF/FYVE/PH
H01-1205	LIN7C	NP_060832	NM_018362	L27/PDZ
H01-1206	DEPDC1B	NP_060839	NM_018369	DEP/RhoGAP
H01-1207	SYNJ2BP	NP_060843	NM_018373	PDZ
H01-1208	PLCXD1	NP_060860	NM_018390	PI-PLC-X
H01-1209	CENTA2	NP_060874	NM_018404	ArfGap/PH
H01-1210	STYK1	NP_060893	NM_018423	TyrKc
H01-1211	PAG	NP_060910	NM_018440	
H01-1212	PPM2C	NP_060914	NM_018444	PP2C
H01-1213	TIP120A	NP_060918	NM_018448	Adaptin_N
H01-1214	ARHGAP15	NP_060930	NM_018460	PH/RhoGAP
H01-1215	TOPK	NP_060962	NM_018492	pkinase
H01-1216	MARK1	NP_061120	NM_018650	S_TKc/TyrKc/UBA/KA1
H01-1217	ANLN	NP_061155	NM_018685	PH
H01-1218	ERBB2IP	NP_061165	NM_018695	PDZ
H01-1219	MYO5C	NP_061198	NM_018728	MYSc/Smc/Myosin_tail/ERM/DIL
H01-1220	LOC55971	NP_061330	NM_018842	SH3

H01-1221	PDE7B	NP_061818	NM_018945	PDEase
H01-1222	SH3BP1	NP_061830	NM_018957	BAR/RhoGAP
H01-1223	UBASH3A	NP_061834	NM_018961	UBP14/SH3/GpmB
H01-1224	SNTG1	NP_061840	NM_018967	PDZ/PH
H01-1225	SNTG2	NP_061841	NM_018968	PDZ
H01-1226	PRKWNK1	NP_061852	NM_018979	S_TKc
H01-1227	SSH1	NP_061857	NM_018984	DSPc
H01-1228	SH3TC1	NP_061859	NM_018986	SH3
H01-1229	CXorf9	NP_061863	NM_018990	SH3/SAM
H01-1230	RIN2	NP_061866	NM_018993	VPS9/RA
H01-1231	TOLLIP	NP_061882	NM_019009	C2/CUE
H01-1232	PLEKHA5	NP_061885	NM_019012	PH/Smc
H01-1233	RHOF	NP_061907	NM_019034	RHO
H01-1234	APBB1IP	NP_061916	NM_019043	RA/PH
H01-1235	MTMR12	NP_061934	NM_019061	
H01-1236	CCNJ	NP_061957	NM_019084	cyclin_C
H01-1237	ARFRP2	NP_061960	NM_019087	arf
H01-1238	PLEKHA3	NP_061964	NM_019091	PH
H01-1239	ARHGEF3	NP_062455	NM_019555	RhoGEF
H01-1240	PARD3	NP_062565	NM_019619	PDZ/Prc
H01-1241	PPP4R2	NP_062827	NM_019853	
H01-1242	GSK3A	NP_063937	NM_019884	S_TKc
H01-1243	INPP5E	NP_063945	NM_019892	IPPc
H01-1244	GRIPAP1	NP_064522	NM_020137	
H01-1245	SH3GLB2	NP_064530	NM_020145	BAR
H01-1246	SAR1A	NP_064535	NM_020150	SAR
H01-1247	PAK6	NP_064553	NM_020168	PBD/S_TKc
H01-1248	DUSP22	NP_064570	NM_020185	DSPc
H01-1249	KNSL7	NP_064627	NM_020242	KISc
H01-1250	CABC1	NP_064632	NM_020247	AarF
H01-1251	ARRB1	NP_064647	NM_020251	arrestin
H01-1252	CCNL1	NP_064703	NM_020307	CYCLIN/bZIP
H01-1253	ACVR1B	NP_064733	NM_020328	Activin_recp/pkinase
H01-1254	NKIRAS1	NP_065078	NM_020345	ras
H01-1255	C20orf32	NP_065089	NM_020356	SH3
H01-1256	RPGRIP1	NP_065099	NM_020366	Smc/C2
H01-1257	HRASLS	NP_065119	NM_020386	
H01-1258	RAB25	NP_065120	NM_020387	RAB
H01-1259	LOC57117	NP_065128	NM_020395	PHD
H01-1260	GOPC	NP_065132	NM_020399	PDZ
H01-1261	ADCK1	NP_065154	NM_020421	AarF
H01-1262	PACE-1	NP_065156	NM_020423	pkinase
H01-1263	CAMK1G	NP_065172	NM_020439	S_TKc
H01-1264	EPHA8	NP_065387	NM_020526	EPH_lbd/FN3/TyrKc/SAM
H01-1265	ADCY2	NP_065433	NM_020546	guanylate_cyc
H01-1266	AMHR2	NP_065434	NM_020547	Activin_recp/pkinase
H01-1267	RIPK4	NP_065690	NM_020639	pkinase/Arp/ank
H01-1268	RCN3	NP_065701	NM_020650	FRQ1
H01-1269	RHOJ	NP_065714	NM_020663	RHO
H01-1270	CLK4	NP_065717	NM_020666	S_TKc
H01-1271	RAB22A	NP_065724	NM_020673	ras

H01-1272	CASKIN2	NP_065804	NM_020753	Arp/SAM
H01-1273	CASKIN1	NP_065815	NM_020764	Arp/SAM
H01-1274	CGN	NP_065821	NM_020770	Myosin_tail
H01-1275	SYT4	NP_065834	NM_020783	C2
H01-1276	LRRC7	NP_065845	NM_020794	PDZ
H01-1277	PACSIN1	NP_065855	NM_020804	FCH/SH3
H01-1278	ARHGAP21	NP_065875	NM_020824	PDZ/PH/RhoGAP
H01-1279	SYT13	NP_065877	NM_020826	C2
H01-1280	WDFY1	NP_065881	NM_020830	COG2319
H01-1281	OSBPL8	NP_065892	NM_020841	PH/Oxysterol_BP
H01-1282	PITPNM2	NP_065896	NM_020845	IP_trans/DDHD/SMP2
H01-1283	SHRM	NP_065910	NM_020859	PDZ/SbcC
H01-1284	STIM2	NP_065911	NM_020860	SAM/Smc
H01-1285	USP28	NP_065937	NM_020886	UCH-2
H01-1286	OSBPL5	NP_065947	NM_020896	PH/Oxysterol_BP
H01-1287	PLEKHA4	NP_065955	NM_020904	PH
H01-1288	PRKWNK3	NP_065973	NM_020922	S_TKc
H01-1289	CPNE5	NP_065990	NM_020939	C2/VWA
H01-1290	PRX	NP_066007	NM_020956	PDZ
H01-1291	MAGI-3	NP_066016	NM_020965	GuKc/WW/PDZ
H01-1292	APS	NP_066189	NM_020979	PH/SH2
H01-1293	ADCY6	NP_066193	NM_020983	guanylate_cyc
H01-1294	ANK3	NP_066267	NM_020987	Arp/ank/ZU5/DEATH
H01-1295	GNAO1	NP_066268	NM_020988	G-alpha
H01-1296	PDLIM1	NP_066272	NM_020992	PDZ/LIM
H01-1297	SNTB1	NP_066301	NM_021021	PDZ/PH
H01-1298	RAP2A	NP_066361	NM_021033	RAS
H01-1299	TSC2	NP_066400	NM_021056	Tuberin/Rap_GAP
H01-1300	ARGBP2	NP_066547	NM_021069	Sorb/SH3
H01-1301	DLG3	NP_066943	NM_021120	PDZ/SH3/GuKc
H01-1302	PPP3CB	NP_066955	NM_021132	PP2Ac
H01-1303	RPS6KA2	NP_066958	NM_021135	S_TKc
H01-1304	UNG2	NP_066970	NM_021147	cyclin
H01-1305	COTL1	NP_066972	NM_021149	ADF
H01-1306	TRIB3	NP_066981	NM_021158	S_TKc
H01-1307	RAP1GDS1	NP_066982	NM_021159	SRP1
H01-1308	RAB40C	NP_066991	NM_021168	RAB
H01-1309	RAP2C	NP_067006	NM_021183	RAS
H01-1310	PLEKHB1	NP_067023	NM_021200	PH
H01-1311	SRPRB	NP_067026	NM_021203	arf
H01-1312	RHOU	NP_067028	NM_021205	RHO
H01-1313	MYL7	NP_067046	NM_021223	FRQ1
H01-1314	RRAGD	NP_067067	NM_021244	COG1100
H01-1315	RAB18	NP_067075	NM_021252	RAB
H01-1316	AFAP	NP_067651	NM_021638	PH
H01-1317	TRIB2	NP_067675	NM_021643	S_TKc
H01-1318	SMAP1	NP_068759	NM_021940	ArfGap
H01-1319	ABR	NP_068781	NM_021962	RhoGEF/C2/RhoGAP
H01-1320	UBE2V1	NP_068823	NM_021988	UQ_con
H01-1321	DEF6	NP_071330	NM_022047	PH
H01-1322	CSNK1G1	NP_071331	NM_022048	pkinase

H01-1323	SH2D4A	NP_071354	NM_022071	SH2
H01-1324	DUSP21	NP_071359	NM_022076	DSPc
H01-1325	LOC63928	NP_071380	NM_022097	FRQ1
H01-1326	FKBPL	NP_071393	NM_022110	TPR
H01-1327	KIF13A	NP_071396	NM_022113	KISc/Smc
H01-1328	SAMSN1	NP_071419	NM_022136	SAM
H01-1329	RRAGC	NP_071440	NM_022157	COG1100
H01-1330	LIN7B	NP_071448	NM_022165	L27/PDZ
H01-1331	ITGB1BP1	NP_071729	NM_022334	PTB
H01-1332	RAB38	NP_071732	NM_022337	RAB
H01-1333	ZFYVE20	NP_071735	NM_022340	FYVE
H01-1334	KIF9	NP_071737	NM_022342	kinesin
H01-1335	EFCBP1	NP_071746	NM_022351	COG1359
H01-1336	ARL6IP2	NP_071769	NM_022374	GBP
H01-1337	GNB1L	NP_071891	NM_053004	WD40
H01-1338	RAB17	NP_071894	NM_022449	ras
H01-1339	RAB3IP	NP_071901	NM_022456	Myosin_tail
H01-1340	MPP5	NP_071919	NM_022474	PDZ/SH3/GuKc
H01-1341	CENTD3	NP_071926	NM_022481	SAM/PH/ArfGap/RhoGAP
H01-1342	TNS	NP_072174	NM_022648	PTPc_motif/SH2/PTB
H01-1343	RASA1	NP_072179	NM_022650	SH3/PH/RasGAP
H01-1344	DUSP6	NP_073143	NM_022652	RHOD/DSPc
H01-1345	LOC64744	NP_073570	NM_022733	ArfGap
H01-1346	LPPR2	NP_073574	NM_022737	PAP2
H01-1347	SMURF2	NP_073576	NM_022739	HUL4
H01-1348	TENS1	NP_073585	NM_022748	SH2/PTB
H01-1349	CERK	NP_073603	NM_022766	LCB5
H01-1350	EPS8L2	NP_073609	NM_022772	PTB/SH3
H01-1351	OSBPL11	NP_073613	NM_022776	PH/Oxysterol_BP
H01-1352	DEPDC6	NP_073620	NM_022783	DEP/PDZ
H01-1353	FLJ23588	NP_073622	NM_022785	FRQ1
H01-1354	KLC2	NP_073733	NM_022822	MreC/PilF
H01-1355	MARCKSL1	NP_075385	NM_023009	
H01-1356	RASL11B	NP_076429	NM_023940	RAS
H01-1357	MGC1136	NP_076930	NM_024025	DSPc
H01-1358	MGC8407	NP_076951	NM_024046	S_TKc
H01-1359	ZFYVE21	NP_076976	NM_024071	FYVE
H01-1360	CARD14	NP_077015	NM_024110	Myosin_tail/PDZ/GuKc
H01-1361	PLEKHF1	NP_077286	NM_024310	FYVE
H01-1362	PLA2G4A	NP_077734	NM_024420	PLAc
H01-1363	NOX5	NP_078781	NM_024505	FRQ1/Ferric_reduct
H01-1364	FYCO1	NP_078789	NM_024513	RUN/Smc/FYVE
H01-1365	EFCAB1	NP_078869	NM_024593	FRQ1
H01-1366	HECTD3	NP_078878	NM_024602	HECTc
H01-1367	ARHGAP10	NP_078881	NM_024605	SH3
H01-1368	PPP1R3B	NP_078883	NM_024607	CBM_21
H01-1369	PLEKHF2	NP_078889	NM_024613	PH/FYVE
H01-1370	FLJ12604	NP_078897	NM_024621	PH
H01-1371	ASB13	NP_078977	NM_024701	Arp/ank
H01-1372	C20orf23	NP_078980	NM_024704	kinesin/PX
H01-1373	MCTP1	NP_078993	NM_024717	C2

H01-1374	C9orf86	NP_078994	NM_024718	ras
H01-1375	MICAL-L2	NP_078999	NM_024723	ERM
H01-1376	CLMN	NP_079010	NM_024734	SAC6
H01-1377	NEK11	NP_079076	NM_024800	S_TKc
H01-1378	RABEP2	NP_079092	NM_024816	Rabaptin
H01-1379	AYTL2	NP_079106	NM_024830	FRQ1
H01-1380	RIN3	NP_079108	NM_024832	VPS9
H01-1381	FLJ21687	NP_079135	NM_024859	PDZ
H01-1382	DOK3	NP_079148	NM_024872	IRS
H01-1383	ADCK4	NP_079152	NM_024876	AarF
H01-1384	FLJ13265	NP_079153	NM_024877	cyclin
H01-1385	FLJ11535	NP_079164	NM_024888	PAP2
H01-1386	RIN3	NP_079168	NM_024892	SH2
H01-1387	PDZK7	NP_079171	NM_024895	PDZ
H01-1388	PLEKHH3	NP_079203	NM_024927	PH/MyTH4/B41
H01-1389	PHC3	NP_079223	NM_024947	FCS/SAM
H01-1390	MCF2L	NP_079255	NM_024979	SEC14/SPEC/RhoGEF
H01-1391	ARL14	NP_079323	NM_025047	arf
H01-1392	YSK4	NP_079328	NM_025052	S_TKc
H01-1393	LAK	NP_079420	NM_025144	MHCK_EF2_kinase
H01-1394	RAB11FIP1	NP_079427	NM_025151	C2
H01-1395	LRRC1	NP_079444	NM_025168	COG4886
H01-1396	DEPDC2	NP_079446	NM_025170	PH/DEP/PDZ
H01-1397	ITPKC	NP_079470	NM_025194	IPK
H01-1398	TRIB1	NP_079471	NM_025195	S_TKc
H01-1399	EFHD1	NP_079478	NM_025202	FRQ1
H01-1400	I-4	NP_079486	NM_025210	
H01-1401	GKAP1	NP_079487	NM_025211	Smc
H01-1402	SPTBN4	NP_079489	NM_025213	SAC6/SPEC
H01-1403	TNKS2	NP_079511	NM_025235	Arp/SAM/PARP
H01-1404	RAPH1	NP_079528	NM_025252	RA/PH
H01-1405	CENTB5	NP_085152	NM_030649	PH/ArfGap/Arp
H01-1406	PTPRO	NP_109596	NM_030671	PTPc
H01-1407	LOC388462	NP_109597	NM_030672	RhoGAP
H01-1408	OSBP2	NP_110385	NM_030758	PH/Oxysterol_BP
H01-1409	ILKAP	NP_110395	NM_030768	PP2Cc
H01-1410	SGPP1	NP_110418	NM_030791	PAP2
H01-1411	STK33	NP_112168	NM_030906	S_TKc
H01-1412	C1orf89	NP_112169	NM_030907	ras
H01-1413	CDADC1	NP_112173	NM_030911	ComEB
H01-1414	CCNL2	NP_112199	NM_030937	CYCLIN
H01-1415	NUAK2	NP_112214	NM_030952	S_TKc
H01-1416	RAB1B	NP_112243	NM_030981	ras
H01-1417	CABP2	NP_112481	NM_031204	FRQ1
H01-1418	KIF18A	NP_112494	NM_031217	kinesin
H01-1419	CDC2L5	NP_112557	NM_031267	S_TKc
H01-1420	RAB33B	NP_112586	NM_031296	RAB
H01-1421	ARHGAP24	NP_112595	NM_031305	RhoGAP
H01-1422	COL4A3BP	NP_112729	NM_031361	PH/START
H01-1423	MARK4	NP_113605	NM_031417	S_TKc/RIO/TyrKc/UBA
H01-1424	C9orf58	NP_113614	NM_031426	FRQ1

H01-1425	RASSF5	NP_113625	NM_031437	C1/RA
H01-1426	RPS6KL1	NP_113652	NM_031464	MIT/S_TKc
H01-1427	CALN1	NP_113656	NM_031468	FRQ1
H01-1428	URP2	NP_113659	NM_031471	B41/PH
H01-1429	SH3KBP1	NP_114098	NM_031892	SH3
H01-1430	USP26	NP_114113	NM_031907	UBP14/UCH-2
H01-1431	RAB34	NP_114140	NM_031934	RAB
H01-1432	CENTG3	NP_114152	NM_031946	RAS/PH/ArfGap
H01-1433	NYD-SP11	NP_114157	NM_031951	WD40
H01-1434	CCNB1	NP_114172	NM_031966	COG5024
H01-1435	MAP2K6	NP_114365	NM_031988	S_TKc
H01-1436	RASSF4	NP_114412	NM_032023	RA
H01-1437	STK22D	NP_114417	NM_032028	S_TKc
H01-1438	FKSG42	NP_114421	NM_032032	RhoGAP
H01-1439	TSSK6	NP_114426	NM_032037	S_TKc
H01-1440	NCALD	NP_114430	NM_032041	FRQ1
H01-1441	PLEKHN1	NP_115505	NM_032129	PH
H01-1442	PPP1R1B	NP_115568	NM_032192	
H01-1443	FLJ23356	NP_115613	NM_032237	pkinase
H01-1444	TBC1D3B	NP_115634	NM_032258	TBC
H01-1445	PSD2	NP_115665	NM_032289	Sec7/PH
H01-1446	SGIP1	NP_115667	NM_032291	Adap_comp_sub
H01-1447	GARNL3	NP_115669	NM_032293	Rap_GAP/CNH
H01-1448	SYT3	NP_115674	NM_032298	C2
H01-1449	EFCAB2	NP_115704	NM_032328	FRQ1
H01-1450	CAPNS2	NP_115706	NM_032330	FRQ1
H01-1451	WNK4	NP_115763	NM_032387	S_TKc
H01-1452	PINK1	NP_115785	NM_032409	pkinase
H01-1453	BRSK1	NP_115806	NM_032430	S_TKc
H01-1454	AP1M1	NP_115882	NM_032493	Adap_comp_sub
H01-1455	ARHGAP9	NP_115885	NM_032496	SH3/PH/RhoGAP
H01-1456	PARD6G	NP_115899	NM_032510	PB1/PDZ
H01-1457	PDZK4	NP_115901	NM_032512	PDZ
H01-1458	KIAA1914	NP_115939	NM_032550	PH
H01-1459	DAB2IP	NP_115941	NM_032552	C2/RasGAP/Smc
H01-1460	KIF2B	NP_115948	NM_032559	kinesin
H01-1461	USP32	NP_115971	NM_032582	FRQ1/UBP12/UCH-2
H01-1462	PPP1R9B	NP_115984	NM_032595	PDZ/Smc
H01-1463	CAPS2	NP_115995	NM_032606	FRQ1
H01-1464	MYO18B	NP_115997	NM_032608	MYSc
H01-1465	PTP4A3	NP_116000	NM_032611	CDC14
H01-1466	LNX1	NP_116011	NM_032622	PDZ
H01-1467	PLEKHA8	NP_116028	NM_032639	PH
H01-1468	BLK	NP_116046	NM_001715	SH3/SH2/TyrKc
H01-1469	EFCAB4B	NP_116069	NM_032680	FRQ1
H01-1470	PLCD4	NP_116115	NM_032726	PH/PLC/C2
H01-1471	PTPN5	NP_116170	NM_032781	PTPc
H01-1472	C9orf100	NP_116207	NM_032818	RhoGEF
H01-1473	MASTL	NP_116233	NM_032844	S_TKc/S_TKc
H01-1474	RAB2B	NP_116235	NM_032846	RAB
H01-1475	ZFYVE19	NP_116239	NM_032850	FYVE

H01-1476	HSH2D	NP_116244	NM_032855	SH2
H01-1477	TNS4	NP_116254	NM_032865	SH2/PTB
H01-1478	ARHGAP19	NP_116289	NM_032900	RhoGAP
H01-1479	PPP1R16A	NP_116291	NM_032902	Arp
H01-1480	RERG	NP_116307	NM_032918	RAS
H01-1481	SYTL2	NP_116561	NM_032943	C2
H01-1482	STK31	NP_116562	NM_032944	TUDOR/pkinase
H01-1483	MAPKAPK2	NP_116584	NM_032960	S_TKc
H01-1484	SEC22L3	NP_116752	NM_032970	
H01-1485	ARHGEF4	NP_127462	NM_032995	SH3/RhoGEF
H01-1486	PCTK1	NP_148979	NM_033019	S_TKc
H01-1487	SYDE1	NP_149014	NM_033025	RhoGAP
H01-1488	MACF1	NP_149033	NM_033044	SAC6/spectrin
H01-1489	RTKN	NP_149035	NM_033046	PH
H01-1490	MPP4	NP_149055	NM_033066	L27/PDZ/SH3/GuKc
H01-1491	RHPN2	NP_149094	NM_033103	HR1/BRO1/PDZ
H01-1492	STON2	NP_149095	NM_033104	Adap_comp_sub
H01-1493	NEK9	NP_149107	NM_033116	S_TKc/ATS1
H01-1494	MYLK2	NP_149109	NM_033118	S_TKc
H01-1495	PLCZ1	NP_149114	NM_033123	PLC/C2
H01-1496	PSKH2	NP_149117	NM_033126	S_TKc
H01-1497	SCIN	NP_149119	NM_033128	GEL
H01-1498	TRIM23	NP_150231	NM_033228	RING/BBC/arf
H01-1499	PPP1R14A	NP_150281	NM_033256	
H01-1500	ERN2	NP_150296	NM_033266	S_TKc/PUG
H01-1501	CDC14A	NP_201570	NM_033313	CDC14
H01-1502	CDC14B	NP_201589	NM_033332	CDC14
H01-1503	CAV3	NP_203123	NM_033337	Caveolin
H01-1504	RGS8	NP_203131	NM_033345	RGS
H01-1505	KRAS	NP_203524	NM_033360	RAS
H01-1506	MYO1C	NP_203693	NM_033375	MYSc
H01-1507	CDC2	NP_203698	NM_033379	S_TKc
H01-1508	MAPK8IP3	NP_203750	NM_033392	SbcC
H01-1509	PDE5A	NP_246273	NM_033437	GAF/PDEase
H01-1510	FCHSD1	NP_258260	NM_033449	FCH/SH3
H01-1511	ARHGAP18	NP_277050	NM_033515	RhoGAP
H01-1512	MRLC2	NP_291024	NM_033546	FRQ1
H01-1513	MGC16733	NP_291025	NM_033547	Adaptin_N
H01-1514	CCNDBP1	NP_411241	NM_037370	
H01-1515	CDC42	NP_426359	NM_044472	RHO
H01-1516	CORO2A	NP_438171	NM_052820	WD40
H01-1517	CDK2	NP_439892	NM_052827	S_TKc
H01-1518	TSSK3	NP_443073	NM_052841	S_TKc
H01-1519	ADCK2	NP_443085	NM_052853	AarF/ABC1
H01-1520	SNAG1	NP_443102	NM_052870	SH3/PX
H01-1521	STX1B2	NP_443106	NM_052874	Syntaxin
H01-1522	RHPN1	NP_443156	NM_052924	HR1/BRO1/PDZ
H01-1523	NOSTRIN	NP_443178	NM_052946	SH3
H01-1524	HAK	NP_443179	NM_052947	IG/MHCK_EF2_kinase
H01-1525	SNX26	NP_443180	NM_052948	SH3/RhoGAP
H01-1526	CDK10	NP_443713	NM_052987	S_TKc

H01-1527	TSSK2	NP_443732	NM_053006	S_TKc
H01-1528	GLMN	NP_444504	NM_053274	
H01-1529	IHPK3	NP_473452	NM_054111	IPK
H01-1530	CIB3	NP_473454	NM_054113	FRQ1
H01-1531	DUSP4	NP_476499	NM_057158	DSPc
H01-1532	CCNE1	NP_476530	NM_057182	cyclin
H01-1533	UBE2J2	NP_477515	NM_058167	UQ_con
H01-1534	CDKN1A	NP_510867	NM_078467	CDI
H01-1535	MYO18A	NP_510880	NM_078471	PDZ/MYSc
H01-1536	CDKN2D	NP_524145	NM_079421	Arp
H01-1537	MYL1	NP_524146	NM_079422	FRQ1
H01-1538	MYL6	NP_524149	NM_079425	FRQ1
H01-1539	PTP4A2	NP_536316	NM_080391	CDC14
H01-1540	PTPN2	NP_536348	NM_080423	PTPc
H01-1541	GNAS	NP_536351	NM_080426	G-alpha
H01-1542	GAB2	NP_536739	NM_080491	PH
H01-1543	AP1G2	NP_536806	NM_080545	Adaptin_N/Alpha_adaptinC2
H01-1544	PTPN6	NP_536859	NM_080549	SH2/PTPc
H01-1545	PTPN7	NP_542156	NM_080589	PTPc
H01-1546	CAPS	NP_542157	NM_080590	FRQ1
H01-1547	PTPN11	NP_542168	NM_080601	SH2/SH2/PTPc
H01-1548	DUSP15	NP_542178	NM_080611	DSPc
H01-1549	GAB3	NP_542179	NM_080612	PH
H01-1550	PTPN13	NP_542416	NM_080685	B41/PDZ/PTPc
H01-1551	SYTL4	NP_542775	NM_080737	RPH3A_effector/C2
H01-1552	C20orf152	NP_543024	NM_080834	cNMP_binding
H01-1553	STK35	NP_543026	NM_080836	S_TKc
H01-1554	PTPRA	NP_543031	NM_080841	PTPc
H01-1555	SOCS4	NP_543143	NM_080867	SH2/SOCS
H01-1556	ASB10	NP_543147	NM_080871	Arp/ank
H01-1557	ASB11	NP_543149	NM_080873	Arp/ank/SOCS
H01-1558	ASB5	NP_543150	NM_080874	Arp/ank
H01-1559	DUSP19	NP_543152	NM_080876	DSPc
H01-1560	RAB40A	NP_543155	NM_080879	RAB
H01-1561	PTPRC	NP_563579	NM_080922	HRD1/FN3/PTPc
H01-1562	ASB14	NP_569058	NM_130387	Arp/SOCS
H01-1563	ASB12	NP_569059	NM_130388	Arp/ank
H01-1564	PTPRD	NP_569077	NM_130393	IG/FN3/PTPc
H01-1565	PTPRE	NP_569119	NM_130435	PTPc
H01-1566	PTPRF	NP_569707	NM_130440	IG/FN3/PTPc
H01-1567	RAB24	NP_570137	NM_130781	ras
H01-1568	RGS18	NP_570138	NM_130782	RGS
H01-1569	TPTE2	NP_570141	NM_130785	CDC14
H01-1570	AP2A1	NP_570603	NM_130787	Adaptin_N/Alpha_adaptinC
H01-1571	SNAP23	NP_570710	NM_130798	t_SNARE
H01-1572	CPNE4	NP_570720	NM_130808	C2/VWA
H01-1573	SNAP25	NP_570824	NM_130811	t_SNARE/t_SNARE
H01-1574	OPA1	NP_570850	NM_130837	dynamin
H01-1575	PTPRN2	NP_570858	NM_130843	PTPc
H01-1576	PTPRR	NP_570897	NM_130846	PTPc
H01-1577	GIPC3	NP_573568	NM_133261	PDZ

H01-1578	FER1L3	NP_579899	NM_133337	C2/DysFN/C2
H01-1579	PLCD3	NP_588614	NM_133373	PH/PLC
H01-1580	GEFT	NP_597840	NM_133483	RhoGEF
H01-1581	NEK7	NP_598001	NM_133494	S_TKc
H01-1582	SYN3	NP_598344	NM_133633	Synapsin
H01-1583	RHOV	NP_598378	NM_133639	RHO
H01-1584	ZAK	NP_598407	NM_133646	pkinase
H01-1585	HPCAL1	NP_602293	NM_134421	FRQ1
H01-1586	CREB1	NP_604391	NM_134442	pKID/bZIP
H01-1587	RPIB9	NP_612147	NM_138290	RUN
H01-1588	LRSAM1	NP_612370	NM_138361	ERM
H01-1589	KIF12	NP_612433	NM_138424	kinesin
H01-1590	FAM83F	NP_612444	NM_138435	Cls
H01-1591	ARL11	NP_612459	NM_138450	arf
H01-1592	RAB3C	NP_612462	NM_138453	ras
H01-1593	KIF23	NP_612565	NM_138555	kinesin/Smc
H01-1594	PP1R8	NP_612568	NM_138558	
H01-1595	CTTN	NP_612632	NM_138565	HS1_rep/SH3
H01-1596	GGA3	NP_619525	NM_138619	VHS/GAT/Alpha_adaptinC2
H01-1597	GGA2	NP_619581	NM_138640	VHS
H01-1598	PPP1R14B	NP_619634	NM_138689	
H01-1599	CALML6	NP_619650	NM_138705	FRQ1
H01-1600	RHOT2	NP_620124	NM_138769	RHO/ras
H01-1601	SYTL5	NP_620135	NM_138780	RPH3A_effector/C2
H01-1602	ARL8A	NP_620150	NM_138795	arf
H01-1603	MAPK1	NP_620407	NM_138957	S_TKc
H01-1604	MAPK10	NP_620448	NM_138982	S_TKc
H01-1605	MYO3B	NP_620482	NM_138995	S_TKc/MYSc
H01-1606	MAPK14	NP_620583	NM_139014	S_TKc
H01-1607	MAPK15	NP_620590	NM_139021	S_TKc
H01-1608	MAPK7	NP_620603	NM_139034	S_TKc
H01-1609	MAPK8	NP_620637	NM_139049	S_TKc
H01-1610	EPS8L3	NP_620641	NM_139053	PTB/SH3
H01-1611	CSNK1D	NP_620693	NM_139062	pkinase
H01-1612	MAPK9	NP_620709	NM_139070	S_TKc
H01-1613	MAPKAPK5	NP_620777	NM_139078	S_TKc
H01-1614	ALS2CR7	NP_631897	NM_139158	S_TKc
H01-1615	CENTD2	NP_631920	NM_139181	PH/ArfGap/RhoGAP
H01-1616	CENTD1	NP_631921	NM_139182	PH/RhoGAP/PH
H01-1617	EPS8L1	NP_631943	NM_139204	PTB/SH3
H01-1618	GRK7	NP_631948	NM_139209	RGS/S_TKc
H01-1619	FGD4	NP_640334	NM_139241	RhoGEF/FYVE/PH
H01-1620	PPM1L	NP_640338	NM_139245	PP2Cc
H01-1621	ADCY4	NP_640340	NM_139247	guanylate_cyc
H01-1622	AKAP1	NP_644804	NM_139275	
H01-1623	STAT3	NP_644805	NM_139276	STAT/STAT_bind/SH2
H01-1624	TA-PP2C	NP_644812	NM_139283	PTC1
H01-1625	AKAP4	NP_647450	NM_139289	
H01-1626	AMPH	NP_647477	NM_139316	BAR/SH3
H01-1627	YWHAB	NP_647539	NM_139323	14-3-3
H01-1628	BIN1	NP_647601	NM_139351	BAR/SH3

H01-1629	MATK	NP_647612	NM_139355	SH3/SH2/TyrKc
H01-1630	RGS3	NP_652760	NM_144489	RGS
H01-1631	AKAP12	NP_653080	NM_144497	
H01-1632	GNAT1	NP_653082	NM_144499	G-alpha
H01-1633	ZFYVE27	NP_653189	NM_144588	FYVE
H01-1634	RHEBL1	NP_653194	NM_144593	RAS
H01-1635	PPM1M	NP_653242	NM_144641	PP2Cc
H01-1636	CAPSL	NP_653248	NM_144647	FRQ1
H01-1637	PXDNL	NP_653252	NM_144651	An_peroxidase/vwc
H01-1638	WDR66	NP_653269	NM_144668	WD40/FRQ1
H01-1639	HIPK4	NP_653286	NM_144685	S_TKc
H01-1640	C3orf48	NP_653315	NM_144714	PP2Cc
H01-1641	AKAP13	NP_658913	NM_144767	RhoGEF
H01-1642	SOCS5	NP_659198	NM_144949	SH2/SOCS
H01-1643	FLJ30058	NP_659404	NM_144967	RhoGAP
H01-1644	ANKRD23	NP_659431	NM_144994	NLS/Arp/ank
H01-1645	ARL13B	NP_659433	NM_144996	arf
H01-1646	STK32A	NP_659438	NM_145001	S_TKc
H01-1647	TSNARE1	NP_659440	NM_145003	COG5325
H01-1648	C10orf9	NP_659449	NM_145012	cyclin
H01-1649	CDC42EP5	NP_659494	NM_145057	
H01-1650	STAC3	NP_659501	NM_145064	C1/SH3
H01-1651	CISH	NP_659508	NM_145071	SH2/SOCS
H01-1652	MAP2K5	NP_660145	NM_145162	S_TKc
H01-1653	DIRAS1	NP_660156	NM_145173	RAS
H01-1654	MAP2K7	NP_660186	NM_145185	S_TKc
H01-1655	CABP4	NP_660201	NM_145200	FRQ1
H01-1656	CSNK1A1L	NP_660204	NM_145203	pkinase
H01-1657	VTI1A	NP_660207	NM_145206	
H01-1658	C14orf143	NP_660274	NM_145231	FRQ1
H01-1659	STYX	NP_660294	NM_145251	DSPc
H01-1660	ACVR1C	NP_660302	NM_145259	Activin_recp/GS/pkinase
H01-1661	PLEKHK1	NP_660350	NM_145307	PH
H01-1662	MAP3K6	NP_663159	NM_145319	S_TKc
H01-1663	OSBPL3	NP_663164	NM_145324	PH/Oxysterol_BP
H01-1664	MAP3K7	NP_663306	NM_145333	pkinase
H01-1665	MAP3K7IP2	NP_663317	NM_145342	CUE
H01-1666	MAP4K4	NP_663720	NM_145687	S_TKc/CNH
H01-1667	YWHAZ	NP_663723	NM_145690	14-3-3
H01-1668	AP1B1	NP_663782	NM_145730	Adaptin_N/Alpha_adaptinC2
H01-1669	PHLDB2	NP_665696	NM_145753	Smc/PH
H01-1670	KIFC2	NP_665697	NM_145754	KISc
H01-1671	CHEK2	NP_665861	NM_145862	FHA/S_TKc
H01-1672	ASB3	NP_665862	NM_145863	Arp/ANK/SOCS
H01-1673	ANKS4B	NP_665872	NM_145865	Arp/SAM
H01-1674	ANXA11	NP_665876	NM_145869	annexin
H01-1675	ASB4	NP_665879	NM_145872	Arp/ank
H01-1676	PALM2-AKAP2	NP_671492	NM_147150	
H01-1677	ITSN2	NP_671494	NM_147152	EH/Smc/SH3
H01-1678	PPP3R2	NP_671709	NM_147180	FRQ1
H01-1679	AKAP9	NP_671714	NM_147185	Smc/NtpI

H01-1680	OSBPL9	NP_683707	NM_148909	PH/Oxysterol_BP
H01-1681	EPN2	NP_683723	NM_148921	ENTH
H01-1682	CSNK1E	NP_689407	NM_152221	pkinase
H01-1683	IPMK	NP_689416	NM_152230	IPK/IPK
H01-1684	CDC42EP1	NP_689449	NM_152243	
H01-1685	SYT11	NP_689493	NM_152280	C2
H01-1686	C9orf111	NP_689499	NM_152286	cNMP_binding/RssA
H01-1687	FLJ40342	NP_689560	NM_152347	FRQ1
H01-1688	SGPP2	NP_689599	NM_152386	PAP2
H01-1689	PTPDC1	NP_689635	NM_152422	CDC14
H01-1690	FRMPD2	NP_689641	NM_152428	PDZ
H01-1691	FLJ32810	NP_689645	NM_152432	RhoGAP
H01-1692	SAMD11	NP_689699	NM_152486	SAM
H01-1693	DUSP18	NP_689724	NM_152511	DSPc
H01-1694	FLJ40432	NP_689736	NM_152523	cyclin
H01-1695	ALS2CR19	NP_689739	NM_152526	PDZ
H01-1696	NEK10	NP_689747	NM_152534	S_TKc
H01-1697	FGD5	NP_689749	NM_152536	RhoGEF/PH/FYVE
H01-1698	DKFZp761G058	NP_689755	NM_152542	PP2Cc
H01-1699	RASGEF1B	NP_689758	NM_152545	RasGEF
H01-1700	SH3RF2	NP_689763	NM_152550	RING/SH3
H01-1701	RASEF	NP_689786	NM_152573	FRQ1
H01-1702	SPRED1	NP_689807	NM_152594	WH1
H01-1703	DCAMKL2	NP_689832	NM_152619	DCX/S_TKc
H01-1704	MGC39715	NP_689841	NM_152628	PX/B41
H01-1705	KNDC1	NP_689856	NM_152643	RasGEF
H01-1706	MLKL	NP_689862	NM_152649	TyrKc
H01-1707	RALGPS2	NP_689876	NM_152663	PH
H01-1708	PLD5	NP_689879	NM_152666	Cls
H01-1709	HIPK1	NP_689909	NM_152696	S_TKc
H01-1710	MGC26597	NP_689913	NM_152700	PIPKc
H01-1711	NEK3	NP_689933	NM_152720	S_TKc
H01-1712	DOK6	NP_689934	NM_152721	PTBI
H01-1713	CPNE2	NP_689940	NM_152727	C2/VWA
H01-1714	PDIK1L	NP_690048	NM_152835	S_TKc
H01-1715	ARPC2	NP_690601	NM_152862	p34-Arc
H01-1716	PTK7	NP_690621	NM_152882	IG/TyrKc
H01-1717	DKFZp434K1815	NP_690852	NM_152892	COG4886/WD40
H01-1718	DGKH	NP_690874	NM_152910	PH/C1/DAGK
H01-1719	CPNE1	NP_690908	NM_152931	C2/VWA
H01-1720	LOC129285	NP_694539	NM_152994	
H01-1721	SPATA13	NP_694568	NM_153023	SH3/RhoGEF
H01-1722	FYN	NP_694593	NM_153048	SH3/TyrKc
H01-1723	MTMR3	NP_694691	NM_153051	PTPc_motif/FYVE
H01-1724	KIF19	NP_694941	NM_153209	kinesin
H01-1725	ARHGEF19	NP_694945	NM_153213	RhoGEF
H01-1726	SIPA1	NP_694985	NM_153253	Rap_GAP/PDZ/Smc
H01-1727	SYT14	NP_694994	NM_153262	C2
H01-1728	PLCXD2	NP_695000	NM_153268	PI-PLC-X
H01-1729	SH3PX3	NP_695003	NM_153271	SH3/PX
H01-1730	IHPK1	NP_695005	NM_153273	IPK

H01-1731	UBE1	NP_695012	NM_153280	ThiF/UBACT
H01-1732	LYK5	NP_699166	NM_153335	S_TKc
H01-1733	MGC42105	NP_699192	NM_153361	S_TKc
H01-1734	LNX2	NP_699202	NM_153371	RAD18/PDZ
H01-1735	CAMK1D	NP_705718	NM_153498	S_TKc
H01-1736	Rgr	NP_705843	NM_153615	RasGEF
H01-1737	CPNE9	NP_705899	NM_153635	C2/VWA
H01-1738	FAM43A	NP_710157	NM_153690	PTB
H01-1739	CABYR	NP_722452	NM_153768	RIIa
H01-1740	RASGRF1	NP_722522	NM_153815	RasGEFN/RasGEF
H01-1741	RASGRP2	NP_722541	NM_153819	RasGEF/DAG_PE-bind
H01-1742	MINK1	NP_722549	NM_153827	S_TKc/CNH
H01-1743	RASGRP4	NP_733749	NM_170604	RasGEF/DAG_PE-bind
H01-1744	RASGRP3	NP_733772	NM_170672	RasGEF/DAG_PE-bind
H01-1745	SGK2	NP_733794	NM_170693	S_TKc
H01-1746	SGK3	NP_733827	NM_170709	PX/S_TKc
H01-1747	RASSF1	NP_733835	NM_170717	DAG_PE-bind
H01-1748	TENC1	NP_736610	NM_170754	C1/SH2/PTB
H01-1749	RASSF2	NP_739580	NM_170774	RA
H01-1750	CAMK2A	NP_741960	NM_171825	S_TKc
H01-1751	USP2	NP_741994	NM_171997	UBP5
H01-1752	RAB39B	NP_741995	NM_171998	RAB
H01-1753	CAMK2B	NP_742081	NM_172084	S_TKc
H01-1754	CAMK2D	NP_742126	NM_172128	S_TKc
H01-1755	CAMK2G	NP_751913	NM_172173	S_TKc
H01-1756	CAMKK1	NP_757343	NM_172206	S_TKc
H01-1757	IL16	NP_757366	NM_172217	PDZ
H01-1758	CAPN3	NP_775110	NM_173087	CysPc/Calpain_III/FRQ1
H01-1759	PTK2B	NP_775268	NM_173176	B41/TyrKc/Focal_AT
H01-1760	SNF1LK	NP_775490	NM_173354	S_TKc
H01-1761	UBE2Q2	NP_775740	NM_173469	UQ_con
H01-1762	USH1G	NP_775748	NM_173477	Arp/SAM
H01-1763	CNTD	NP_775749	NM_173478	cyclin
H01-1764	PIP5KL1	NP_775763	NM_173492	PIPKc
H01-1765	MPP7	NP_775767	NM_173496	L27/PDZ/SH3/GuKc
H01-1766	TTBK2	NP_775771	NM_173500	pkinase
H01-1767	EFCAB3	NP_775774	NM_173503	FRQ1
H01-1768	CNKSR3	NP_775786	NM_173515	SAM/PDZ
H01-1769	FLJ38964	NP_775798	NM_173527	ras
H01-1770	CNBD1	NP_775809	NM_173538	cNMP_binding
H01-1771	ANKS6	NP_775822	NM_173551	SAM
H01-1772	FGD2	NP_775829	NM_173558	RhoGEF/FYVE/PH
H01-1773	STK32C	NP_775846	NM_173575	S_TKc
H01-1774	KSR2	NP_775869	NM_173598	pkinase
H01-1775	RNF149	NP_775918	NM_173647	PA/COG5540
H01-1776	EPHA6	NP_775926	NM_173655	TyrKc
H01-1777	ARL10	NP_775935	NM_173664	arf
H01-1778	ARHGEF15	NP_776089	NM_173728	RhoGEF
H01-1779	PDZD8	NP_776152	NM_173791	PDZ/DAG_PE-bind
H01-1780	LOC201175	NP_777579	NM_174919	SH3
H01-1781	TSSK4	NP_777604	NM_174944	S_TKc

H01-1782	SLA2	NP_778252	NM_175077	SH3/SH2
H01-1783	ARFGAP1	NP_783202	NM_175609	ArfGap
H01-1784	TPO	NP_783653	NM_175722	An_peroxidase/CCP/EGF_CA
H01-1785	SYT9	NP_783860	NM_175733	C2
H01-1786	RAB37	NP_783865	NM_175738	ras
H01-1787	RHOC	NP_786886	NM_175744	RHO
H01-1788	TXLNA	NP_787048	NM_175852	Smc
H01-1789	KIS	NP_787062	NM_175866	pkinase/RRM
H01-1790	EXOC8	NP_787072	NM_175876	PH
H01-1791	DUOX1	NP_787954	NM_175940	An_peroxidase/FRQ1/Ferric_reduct
H01-1792	MTMR1	NP_789746	NM_176789	GRAM/PTPc_motif
H01-1793	PDLIM2	NP_789847	NM_176871	PDZ/LIM
H01-1794	INADL	NP_795353	NM_176878	PDZ
H01-1795	PPAP2A	NP_795714	NM_176895	PAP2
H01-1796	SYT2	NP_796376	NM_177402	C2
H01-1797	RAB7B	NP_796377	NM_177403	RAB
H01-1798	PPAP2B	NP_803133	NM_177414	PAP2
H01-1799	KLC3	NP_803136	NM_177417	
H01-1800	PPFIA1	NP_803172	NM_177423	Smc/SAM
H01-1801	STX12	NP_803173	NM_177424	COG5325
H01-1802	RASSF6	NP_803876	NM_177532	RA
H01-1803	PPAP2C	NP_808211	NM_177543	PAP2
H01-1804	CSNK2A1	NP_808228	NM_177560	S_TKc
H01-1805	PPM1A	NP_808821	NM_177952	PP2Cc
H01-1806	DOK5	NP_808874	NM_177959	PTBI
H01-1807	PPM1B	NP_808907	NM_177968	PP2Cc
H01-1808	ARL6	NP_816931	NM_177976	arf
H01-1809	PPM1G	NP_817092	NM_177983	PP2Cc
H01-1810	PAK7	NP_817127	NM_177990	PBD/S_TKc
H01-1811	ASB6	NP_821066	NM_177999	ank
H01-1812	PPP2R4	NP_821070	NM_178003	PTPA
H01-1813	PLA2G4D	NP_828848	NM_178034	PLA2_B
H01-1814	NEK8	NP_835464	NM_178170	S_TKc/ATS1
H01-1815	SPTBN1	NP_842565	NM_178313	SAC6/SPEC
H01-1816	CDC2L2	NP_076916	NM_024011	S_TKc/TyrKc
H01-1817	IRS2	NP_003740	NM_003749	PH/IRS_PTBI
H01-1818	YWHAE	NP_006752	NM_006761	14_3_3
H01-1819	LOC389842	XP_372200	XM_372200	RanBD
H01-1820	DFNB31	NP_056219	NM_015404	PDZ
H01-1821	KIAA0674	XP_376903	XM_376903	Myosin_tail/SbcC/Smc/ERM
H01-1822	L3MBTL3	NP_115814	NM_032438	MBT/SAM
H01-1823	ANKRD13	NP_149112	NM_033121	ANK
H01-1824	MAP3K9	NP_149132	NM_033141	TyrKc/S_TKc
H01-1825	ZFYVE1	NP_067083	NM_021260	GBP/FYVE
H01-1826	DMWD	NP_004934	NM_004943	WD40
H01-1827	SLC25A25	NP_443133	NM_052901	FRQ1
H01-1828	PPFIA3	NP_003651	NM_003660	Smc/SAM
H01-1829	RASGRF2	NP_008840	NM_006909	RasGEF/RhoGEF/PH
H01-1830	STK40	NP_114406	NM_032017	S_TKc
H01-1831	RANBP6	NP_036548	NM_012416	ARM/KAP95
H01-1832	PPP2R2C	NP_065149	NM_020416	WD40/CDC55

H01-1833	PARD6B	NP_115910	NM_032521	PB1/PDZ
H01-1834	LOC90342	XP_001127410	XM_001127410	C2
H01-1835	PLEKHH1	NP_065766	NM_020715	PH/MyTH4/B41
H01-1836	WDR22	NP_003852	NM_003861	WD40
H01-1837	SHD	NP_064594	XM_031857	SH2
H01-1838	MAST1	NP_055790	NM_014975	S_TKc/PDZ
H01-1839	SIPA1L3	NP_055888	NM_015073	Rap_GAP/PDZ
H01-1840	SHF	NP_612365	NM_138356	SH2
H01-1841	C21orf25	XP_032945	XM_032945	C2
H01-1842	MICAL3	NP_065844	NM_020793	SAC6/CH/LIM
H01-1843	DLGAP3	XP_035601	XM_035601	GKAP
H01-1844	FAM62B	NP_065779	NM_020728	C2
H01-1845	LOC440804	XP_036936	XM_036936	SH3/FN3
H01-1846	CABLES2	NP_112492	NM_031215	CYCLIN
H01-1847	DUSP7	NP_001938	NM_001947	DSPc/DSP_MapKP
H01-1848	WDR18	NP_077005	NM_024100	WD40
H01-1849	MAST3	XP_038150	XM_038150	S_TKc/TyrKc/PDZ
H01-1850	LOC91461	NP_612379	NP_612379	pkinase/S_TKc/TyrKc
H01-1851	UNC13A	XP_038604	XM_038604	C2/C2
H01-1852	DUSP16	NP_085143	NM_030640	DSPc/DSP_MapKP
H01-1853	MICAL-L1	NP_203744	NM_033386	CH
H01-1854	ARHGAP20	NP_065860	NM_020809	RhoGAP
H01-1855	SNF1LK2	NP_056006	NM_015191	SPS1/S_TKc
H01-1856	SHC2	XP_001129272	XM_001129272	PTB/SH2
H01-1857	MAP3K1	XP_042066	XM_042066	
H01-1858	ROPN1B	NP_001012337	NM_001012337	RIIa
H01-1859	PLCH1	NP_055811	NM_014996	FRQ1/PLC/C2
H01-1860	USP22	XP_042698	XM_042698	UBP14
H01-1861	SH3RF1	NP_065921	NM_020870	RING/SH3
H01-1862	SOS2	NP_008870	NM_006939	RasGEF
H01-1863	PDP2	NP_065837	NM_020786	PP2C
H01-1864	PIK3R1	NP_852556	NM_181504	SH2
H01-1865	GAPVD1	NP_056450	NM_015635	RasGAP/VPS9
H01-1866	ULK3	NP_056333	NM_015518	S_TKc/TyrKc
H01-1867	MTMR7	NP_004677	NM_004686	PTPc_motif
H01-1868	CIT	NP_009105	NM_007174	Smc/S_TKc/C1/PH/CNH
H01-1869	NEDD4	NP_006145	NM_006154	C2/WW/HUL4/PRP40/HECTc
H01-1870	C20orf74	NP_065076	NM_020343	Rap_GAP
H01-1871	PPFIA4	NP_055868	NM_015053	SAM
H01-1872	WDR75	NP_115544	NM_032168	WD40
H01-1873	PIP5K1C	NP_036530	NM_012398	PIPKc
H01-1874	ZAP70	NP_001070	NM_001079	SH2/TyrKc
H01-1875	RUSC2	NP_055621	NM_014806	RUN/SH3
H01-1876	KIAA1026	NP_056024	NM_015209	Smc/ERM
H01-1877	SWAP70	NP_055870	NM_015055	PH
H01-1878	KIF26A	XP_050278	XM_050278	KISc/kinesin
H01-1879	DNMBP	NP_056036	NM_015221	SH3/RhoGEF/BAR
H01-1880	PPM1H	XP_350881	XM_350880	PP2C/PTC1
H01-1881	LPPR4	NP_055654	NM_014839	PAP2/acidPPc/PgpB
H01-1882	DOCK10	NP_055504	NM_014689	PH
H01-1883	FNBP1	NP_055848	NM_015033	SH3

H01-1884	FGD3	XP_053487	XM_053487	RhoGEF/PH/FYVE
H01-1885	PCTK3	NP_002587	NM_002596	S_TKc
H01-1886	NA	XP_054936	XM_054936	DSPc
H01-1887	MAPK3	NP_002737	NM_002746	S_TKc/TyrKc
H01-1888	LMTK3	XP_055866	XM_055866	TyrKc/SPS1/S_TKc
H01-1889	ATG16L2	NP_203746	NM_033388	WD40
H01-1890	ANKS3	NP_597707	NM_133450	ANK/SAM
H01-1891	LOC126520	NP_001011716	NM_001011716	/S_TKc
H01-1892	SFN	NP_006133	NM_006142	14_3_3
H01-1893	SRGAP2	NP_056141	NM_015326	RhoGAP/SH3
H01-1894	IQGAP3	NP_839943	XM_059223	IQG1/RasGAP
H01-1895	CIB4	NP_001025052	NM_001029881	FRQ1/EFh
H01-1896	FAM58A	NP_689487	NM_152274	CYCLIN
H01-1897	FLJ36748	NP_689619	NM_152406	PH
H01-1898	NCF1	NP_000256	NM_000265	PX/SH3
H01-1899	ANKRD19	NP_001010925	NM_001010925	ANK
H01-1900	PNCK	NP_940854	NM_198452	S_TKc
H01-1901	TCHHL1	NP_001008536	NM_001008536	S_100/MDN1
H01-1902	SHE	NP_001010846	NM_001010846	SH2
H01-1903	NA	XP_060271	XM_060271	
H01-1904	NA	XP_060609	XM_060609	SPEC
H01-1905	NA	XP_061562	XM_061562	RA
H01-1906	NA	XP_062204	XM_062204	14-3-3/BMH1
H01-1906	YWHAZ	NP_003397	NM_003406	14-3-3/BMH1
H01-1907	NA	XP_062330	XM_062330	
H01-1908	NA	XP_062867	XM_062867	ARF/SAR/RAB
H01-1909	LOC647279	XP_935434	XM_930341	SPS1/pkinase/S_TKc/TyrKc
H01-1910	PLA2G4E	XP_523058	XM_523058	PLA2_B
H01-1911	LNX1	NP_116011	NM_032622	PDZ
H01-1912	NA	XP_064197	XM_064197	FRQ1/Efh
H01-1913	LOC124685	XP_064265	XM_064265	FRQ1
H01-1914	LOC390801	XP_064336	XM_064336	S_100
H01-1915	KA36	NP_872303	NM_182497	filament/ERM/Smc
H01-1916	NA	XP_064635	XM_064635	RHO/ras
H01-1917	KIAA1957	XP_065166	XM_065166	C2
H01-1918	RALBP1	NP_006779	XM_065279	RhoGAP/ARID
H01-1919	NA	XP_065311	XM_065311	PI3_PI4_kinase/PI3Kc/TEL1
H01-1920	NA	XP_065724	XM_065724	RA

NA indicates that the refseq accession number was recently removed during the genome annotation process
