# Supplementary material for: siRNA screen of the human signaling proteome identifies the PtdIns(3,4,5)P3-mTOR signaling pathway as a primary regulator of transferrin uptake
Source: Genome Biol. 2007 Jul 19;8(7):R142. doi: 10.1186/gb-2007-8-7-r142 (PMC2323231; doi:10.1186/gb-2007-8-7-r142)
Supplement: Additional data file 3 — Hits from the primary screen. [file gb-2007-8-7-r142-S3.pdf]

Additional data file 3. List of hits from the primary screen. Genes with absolute values of CAsH scores ≥ 0.95 are shown. Genes for which two batches of d-siRNAs targeting the same sequence had consistent effects, are indicated (*). Genes for which two batches of d-siRNAs targeting the two distinct sequences had consistent effects, are indicated (red, **).

UniqueID	Plate#	Row#	Column#	Gene	NP#	NM#	CAsH	F (Screen 20 nM)	F (Screen 100 nM)
H01-1570	17	C	10	AP2A1**	NP_570603	NM_130787.2	**-1.00**	0.75	0.63
H01-0437	5	E	5	AP2M1**	NP_004059	NM_004068.2	**-1.00**	0.55	0.72
H01-0110	2	B	2	ARCN1**	NP_001646	NM_001655.3	**-1.00**	0.67	0.31
H01-1112	12	E	8	COPB1**	NP_057535	NM_016451.1	**-1.00**	0.74	0.63
H01-0891	10	C	3	AP2A2*	NP_036437	NM_012305.1	**-1.00**	0.85	0.82
H01-0196	3	A	4	PDPK1**	NP_002604	NM_002613.1	**-1.00**	0.86	0.80
H01-1548	17	A	12	DUSP15	NP_542178	NM_080611.3	**-1.00**	0.86	0.80
H01-0687	8	B	3	ACTR2*	NP_005713	NM_005722.1	**-1.00**	0.87	0.79
H01-0222	3	C	6	PPP4C	NP_002711	NM_002720.1	**-1.00**	0.87	0.88
H01-1117	12	F	1	CRKRS	NP_057591	NM_016507.1	**-1.00**	0.87	0.72
H01-0317	4	C	5	TTK	NP_003309	NM_003318.2	**-1.00**	0.87	0.86
H01-0192	2	H	12	PDE2A*	NP_002590	NM_002599.1	**-1.00**	0.87	0.74
H01-1799	19	F	11	KLC3	NP_803136	NM_177417.1	**-1.00**	0.88	0.51
H01-0606	7	C	6	AKT1**	NP_005154	NM_005163.1	**-1.00**	0.88	0.76
H01-0341	4	E	5	PTP4A1	NP_003454	NM_003463.2	**-1.00**	0.88	0.86
H01-0572	6	H	8	CLTC**	NP_004850	NM_004859.1	**-1.00**	0.88	0.55
H01-0620	7	D	8	GAK*	NP_005246	NM_005255.1	**-1.00**	0.88	0.67
H01-0347	4	E	11	AXIN1*	NP_003493	NM_003502.1	**-1.00**	0.89	0.81
H01-0711	8	D	3	CPNE6	NP_006023	NM_006032.2	**-1.00**	0.89	0.88
H01-1816	19	H	4	CDC2L2	NP_076916	NM_024011	**-1.00**	0.89	0.81
H01-0136	2	D	4	MPP3*	NP_001923	NM_001932.2	**-1.00**	0.89	0.85
H01-0753	8	G	9	RRAS	NP_006261	NM_006270.2	**-1.00**	0.89	0.80
H01-0230	3	D	2	PRKCA	NP_002728	NM_002737.1	**-1.00**	0.89	0.79
H01-0628	7	E	4	HCLS1*	NP_005326	NM_005335.1	**-1.00**	0.89	0.81
H01-0336	4	D	12	VRK1	NP_003375	NM_003384.1	**-1.00**	0.89	0.67
H01-0319	4	C	7	TYK2*	NP_003322	NM_003331.1	**-1.00**	0.89	0.80
H01-0767	8	H	11	STK25	NP_006365	NM_006374.2	**-1.00**	0.90	0.91
H01-0378	4	H	6	RGS20	NP_003693	NM_003702.2	**-1.00**	0.90	0.86
H01-0715	8	D	7	HRBL*	NP_006067	NM_006076.2	**-1.00**	0.90	0.83
H01-0100	2	A	4	INPPL1*	NP_001558	NM_001567.2	**-1.00**	0.90	0.82
H01-0335	4	D	11	VIL2*	NP_003370	NM_003379.3	**-1.00**	0.90	0.76
H01-0179	2	G	11	MYH11	NP_002465	NM_002474.1	**-1.00**	0.91	0.74

H01-0763	8	H	7	CNKSR1*	NP_006305	NM_006314.1	**-1.00**	0.91	0.77
H01-0625	7	E	1	GRB7*	NP_005301	NM_005310.1	**-1.00**	0.91	0.87
H01-0784	9	B	4	DYRK2*	NP_006473	NM_006482.1	**-0.99**	0.91	0.82
H01-0250	3	E	10	PTPRH	NP_002833	NM_002842.1	**-0.99**	0.91	0.78
H01-0228	3	C	12	PRKAR1A**	NP_002725	NM_002734.1	**-0.99**	0.91	0.87
H01-0774	9	A	6	ARPC1A*	NP_006400	NM_006409.2	**-0.99**	0.91	0.82
H01-0206	3	B	2	PLCG1	NP_002651	NM_002660.1	**-0.99**	0.91	0.92
H01-0403	5	B	7	SYNJ1	NP_003886	NM_003895.1	**-0.99**	0.91	0.95
H01-1737	19	A	9	CPNE9*	NP_705899	NM_153635.1	**-0.99**	0.91	0.63
H01-0164	2	F	8	KIF3C*	NP_002245	NM_002254.2	**-0.99**	0.91	0.91
H01-0580	7	A	4	FRAP1**	NP_004949	NM_004958.2	**-0.99**	0.92	0.92
H01-1526	16	H	2	CDK10	NP_443713	NM_052987.1	**-0.99**	0.92	0.84
H01-0402	5	B	6	DDEF2*	NP_003878	NM_003887.1	**-0.99**	0.92	0.87
H01-0461	5	G	5	UBE2L6	NP_004214	NM_004223.1	**-0.98**	0.92	0.99
H01-0721	8	E	1	DYNC1LI2**	NP_006132	NM_006141.1	**-0.98**	0.92	0.90
H01-1684	18	E	4	CDC42EP1	NP_689449	NM_152243.1	**-0.97**	0.92	0.82
H01-0809	9	D	5	YWHAE	NP_006752	NM_006761.2	**-0.97**	0.92	0.91
H01-0171	2	G	3	MAP3K3	NP_002392	NM_002401.2	**-0.97**	0.92	0.78
H01-0749	8	G	5	PRKG1	NP_006249	NM_006258.1	**-0.97**	0.92	0.93
H01-0690	8	B	6	ARL7	NP_005728	NM_005737.2	**-0.97**	0.92	0.85
H01-0261	3	F	9	RAB13	NP_002861	NM_002870.1	**-0.97**	0.92	0.86
H01-0818	9	E	2	CCNI*	NP_006826	NM_006835.2	**-0.97**	0.92	0.93
H01-0709	8	D	1	STK10	NP_005981	NM_005990.1	**-0.97**	0.92	0.84
H01-1820	19	H	8	DFNB31	XP_027012	XM_027012.2	**-0.97**	0.92	0.86
H01-1875	20	E	3	RUSC2**	NP_055621	NM_014806	**-0.97**	0.93	0.79
H01-0614	7	D	2	MAP3K8*	NP_005195	NM_005204.2	**-0.97**	0.93	0.90
H01-0941	10	G	5	STXBP6	NP_054897	NM_014178.4	**-0.96**	0.93	0.90
H01-0106	2	A	10	AKT2*	NP_001617	NM_001626.2	**-0.96**	0.93	0.78
H01-0388	5	A	4	STX10	NP_003756	NM_003765.1	**-0.96**	0.93	0.91
H01-0578	7	A	2	DNM2*	NP_004936	NM_004945.1	**-0.95**	0.93	1.01
H01-0556	6	G	4	TAOK2**	NP_004774	NM_004783.1	**-0.95**	0.93	0.90
H01-0390	5	A	6	RNGTT	NP_003791	NM_003800.1	**0.95**	1.07	1.18
H01-1348	15	A	4	TENS1	NP_073585	NM_022748.6	**0.95**	1.07	1.18
H01-1869	20	D	9	NEDD4	NP_006145	NM_006154.1	**0.96**	1.07	1.34
H01-1144	12	H	4	PSCD1	NP_059430	NM_017456.1	**0.96**	1.07	1.12
H01-0541	6	F	1	LATS1	NP_004681	NM_004690.2	**0.96**	1.07	1.16
H01-1176	13	B	12	STX17	NP_060389	NM_017919.1	**0.96**	1.07	1.21
H01-1683	18	E	3	IPMK*	NP_689416	NM_152230.1	**0.97**	1.07	1.17
H01-1586	17	E	2	CREB1	NP_604391	NM_134442.2	**0.97**	1.07	1.22
H01-1550	17	B	2	PTPN13**	NP_542416	NM_080685.1	**0.97**	1.07	1.22
H01-0507	6	C	3	EPS8*	NP_004438	NM_004447.1	**0.97**	1.07	1.23
H01-1779	19	E	3	PDZD8*	NP_776152	NM_173791.1	**0.97**	1.07	1.23

H01-1309	14	F	1	RAP2C	NP_067006	NM_021183.1	**0.97**	1.07	1.23
H01-0303	4	B	3	AURKC	NP_003151	NM_003160.1	**0.97**	1.07	1.11
H01-0227	3	C	11	PRKAG1	NP_002724	NM_002733.1	**0.97**	1.07	1.14
H01-0615	7	D	3	CRK	NP_005197	NM_005206.2	**0.97**	1.07	1.18
H01-0297	4	A	9	STAT5A	NP_003143	NM_003152.2	**0.98**	1.08	1.26
H01-0202	3	A	10	PIK3C3*	NP_002638	NM_002647.1	**0.98**	1.08	1.09
H01-0992	11	C	8	ARHGAP11A*	NP_055598	NM_014783.1	**0.98**	1.08	1.21
H01-1851	20	C	3	UNC13A	XP_038604	XM_038604.4	**0.98**	1.08	1.14
H01-1299	14	E	3	TSC2**	NP_066400	NM_021056.1	**0.98**	1.08	1.41
H01-1833	20	A	9	PARD6B*	XP_030559	XM_030559.1	**0.98**	1.08	1.14
H01-0285	3	H	9	SH3GL2*	NP_003017	NM_003026.1	**0.98**	1.08	1.07
H01-0331	4	D	7	UBE2V2	NP_003341	NM_003350.2	**0.98**	1.08	1.24
H01-1401	15	E	9	GKAP1*	NP_079487	NM_025211.2	**0.99**	1.08	1.18
H01-0433	5	E	1	CCNG1	NP_004051	NM_004060.2	**0.99**	1.08	1.06
H01-0846	9	G	6	TRIO	NP_009049	NM_007118.1	**0.99**	1.08	1.23
H01-0122	2	C	2	CALM2	NP_001734	NM_001743.3	**0.99**	1.08	1.09
H01-1746	19	B	6	SGK3	NP_733827	NM_170709.1	**0.99**	1.08	1.13
H01-1484	16	D	8	SEC22L3	NP_116752	NM_032970.2	**0.99**	1.08	1.18
H01-1281	14	C	9	OSBPL8	NP_065892	NM_020841.3	**0.99**	1.08	1.16
H01-0207	3	B	3	PLCG2	NP_002652	NM_002661.1	**0.99**	1.08	1.09
H01-0407	5	B	11	MTMR2	NP_003903	NM_016156.2	**0.99**	1.09	1.13
H01-0449	5	F	5	PRKAR2A	NP_004148	NM_004157.1	**1.00**	1.09	1.26
H01-0107	2	A	11	ANXA8	NP_001621	NM_001630.1	**1.00**	1.09	1.27
H01-0785	9	B	5	GNAI3*	NP_006487	NM_006496.1	**1.00**	1.09	1.15
H01-1032	11	F	12	SARM1	NP_055892	NM_015077.1	**1.00**	1.09	1.01
H01-0738	8	F	6	PPP2R5A	NP_006234	NM_006243.1	**1.00**	1.09	1.11
H01-0674	8	A	2	SDCBP	NP_005616	NM_005625.1	**1.00**	1.09	1.16
H01-0266	3	G	2	RAP1A	NP_002875	NM_002884.1	**1.00**	1.09	1.05
H01-0879	10	B	3	RABGAP1**	NP_036329	NM_012197.2	**1.00**	1.09	1.12
H01-0093	1	H	9	ERN1	NP_001424	NM_001433.1	**1.00**	1.09	1.07
H01-1582	17	D	10	SYN3	NP_598344	NM_133633.1	**1.00**	1.09	1.19
H01-0251	3	E	11	PTPRJ**	NP_002834	NM_002843.2	**1.00**	1.10	1.17
H01-1418	15	G	2	KIF18A*	NP_112494	NM_031217.2	**1.00**	1.10	1.09
H01-0248	3	E	8	PTPN9	NP_002824	NM_002833.2	**1.00**	1.10	1.13
H01-0199	3	A	7	PFN2	NP_002619	NM_002628.2	**1.00**	1.10	1.15
H01-1750	19	B	10	CAMK2A	NP_741960	NM_171825.1	**1.00**	1.10	1.20
H01-0365	4	G	5	RGS5	NP_003608	NM_003617.1	**1.00**	1.10	1.21
H01-1882	20	E	10	DOCK10	NP_055504	NM_014689	**1.00**	1.10	1.12
H01-0089	1	H	5	DOCK1	NP_001371	NM_001380.1	**1.00**	1.10	1.22
H01-1893	20	F	9	SRGAP2	NP_056141	NM_015326	**1.00**	1.10	1.10
H01-1894	20	F	10	IQGAP3	NP_839943	XM_059223.4	**1.00**	1.10	1.05
H01-1837	20	B	1	SHD	NP_064594	XM_031857.3	**1.00**	1.10	1.09

H01-1576	17	D	4	PTPRR*	NP_570897	NM_130846.1	**1.00**	1.10	1.23
H01-0262	3	F	10	RAC2	NP_002863	NM_002872.3	**1.00**	1.10	1.06
H01-1788	19	E	12	TXLNA*	NP_787048	NM_175852.1	**1.00**	1.10	1.09
H01-0190	2	H	10	PAK3	NP_002569	NM_002578.1	**1.00**	1.10	1.13
H01-0132	2	C	12	CHML*	NP_001812	NM_001821.1	**1.00**	1.11	1.21
H01-0220	3	C	4	PPP2R3A**	NP_002709	NM_002718.2	**1.00**	1.11	1.13
H01-0904	10	D	4	RAB3GAP2*	NP_036546	NM_012414.2	**1.00**	1.11	1.18
H01-1864	20	D	4	PIK3R1*	NP_852556	XM_043865.4	**1.00**	1.11	1.07
H01-1488	16	D	12	MACF1*	NP_149033	NM_033044.1	**1.00**	1.11	1.06
H01-0731	8	E	11	PIK3CB	NP_006210	NM_006219.1	**1.00**	1.11	1.07
H01-0685	8	B	1	ARPC1B	NP_005711	NM_005720.2	**1.00**	1.11	1.24
H01-0996	11	C	12	KIAA0528*	NP_055617	NM_014802.1	**1.00**	1.11	1.31
H01-0333	4	D	9	VASP*	NP_003361	NM_003370.1	**1.00**	1.11	1.03
H01-1669	18	D	1	PHLDB2**	NP_665696	NM_145753.1	**1.00**	1.11	1.10
H01-0392	5	A	8	RIPK2*	NP_003812	NM_003821.2	**1.00**	1.11	1.58
H01-0334	4	D	10	VAV2*	NP_003362	NM_003371.1	**1.00**	1.11	1.20
H01-0608	7	C	8	ARHE	NP_005159	NM_005168.2	**1.00**	1.11	1.23
H01-1810	19	G	10	PAK7*	NP_817127	NM_177990.1	**1.00**	1.12	0.98
H01-0318	4	C	6	TXK	NP_003319	NM_003328.1	**1.00**	1.12	1.22
H01-1843	20	B	7	DLGAP3	XP_035601	XM_035601.5	**1.00**	1.12	1.08
H01-0031	1	C	7	RYR1*	NP_000531	NM_000540.1	**1.00**	1.12	1.26
H01-0602	7	C	2	ANXA3	NP_005130	NM_005139.1	**1.00**	1.12	1.15
H01-0741	8	F	9	PPP2R5E*	NP_006237	NM_006246.1	**1.00**	1.13	1.27
H01-0816	9	D	12	PLEKHC1**	NP_006823	NM_006832.1	**1.00**	1.13	1.15
H01-0194	3	A	2	PDE7A*	NP_002594	NM_002603.1	**1.00**	1.13	1.19
H01-0981	11	B	9	ULK2	NP_055498	NM_014683.2	**1.00**	1.14	1.33
H01-0330	4	D	6	UBE2N	NP_003339	NM_003348.1	**1.00**	1.14	1.11
H01-0389	5	A	5	CTNNAL1	NP_003789	NM_003798.1	**1.00**	1.14	1.47
H01-0652	7	G	4	PAMCI**	NP_005438	NM_005447.2	**1.00**	1.14	1.20
H01-1448	16	A	8	SYT3	NP_115674	NM_032298.1	**1.00**	1.14	1.08
H01-0296	4	A	8	STAT4*	NP_003142	NM_003151.2	**1.00**	1.15	1.28
H01-0017	1	B	5	PTEN**	NP_000305	NM_000314.1	**1.00**	1.15	1.41
H01-0198	3	A	6	PEX13	NP_002609	NM_002618.1	**1.00**	1.15	1.13
H01-0752	8	G	8	RANBP2*	NP_006258	NM_006267.3	**1.00**	1.15	1.08
H01-0442	5	E	10	DLG1	NP_004078	NM_004087.1	**1.00**	1.16	1.42
H01-0699	8	C	3	STX6	NP_005810	NM_005819.2	**1.00**	1.16	1.23
H01-0155	2	E	11	HCK*	NP_002101	NM_002110.1	**1.00**	1.18	1.11
H01-0223	3	C	7	PPP6C	NP_002712	NM_002721.3	**1.00**	1.20	1.18
