# Supplementary material for: siRNA screen of the human signaling proteome identifies the PtdIns(3,4,5)P3-mTOR signaling pathway as a primary regulator of transferrin uptake
Source: Genome Biol. 2007 Jul 19;8(7):R142. doi: 10.1186/gb-2007-8-7-r142 (PMC2323231; doi:10.1186/gb-2007-8-7-r142)
Supplement: Additional data file 4 — The 21 identified 'high confidence' genes that increase or decrease transferrin uptake in HeLa cells. [file gb-2007-8-7-r142-S4.pdf]

Additional data file 4: Genes increasing or decreasing transferrin uptake in HeLa cells.

Functional Category	Gene Name	Brief Description and/or Alias	Protein domain name
Growth/ mTOR signaling	FRAP1	mTOR	PI3Kc_related/FATC/TEL1
	PDPK1	3-phosphoinositide dependent protein kinase-1	S_TKc/PH_PDK1
	TSC2	tuberin/Rheb GAP	Tuberin/Rap_GAP
	PTEN	PI-3,4,5-trisphosphate 3-phosphatase	CDC14
	AKT1	protein kinase B	PH_Akt/S_TKc/S_TK_X
Misc. signaling	PLEKHC1	mig-2/ECM-actin junctions	B41(split)/Unc112(PH)
	TAOK2	MAP3K17/PSK/MT organization/p38 activation	S_TKc
	PTPN13	FAP-1/PTPL1/	KIND/FERM-C/B41/PDZ/PTPc
	PTPRJ	DEP1	PTPc/FN3
	PPP2R3A	Protein phosphatase 2-PR72/PR130 subunit	-
	PRKAR1A	PKA-regulatory subunit Iα/tse1	CAP_ED
Vesicular transport/ Compartmentalization	CLTC	clathrin heavy chain	Clathrin_propel/CLH/ Clathrin
	AP2M1	AP2μ2 chain	Adap_comp_sub
	AP2A1	AP2α1 chain	Adaptin_N,Alpha_adaptin_C
	ARCN1	coatomer subunit δ	Adap_comp_sub
	COPB1	coatomer subunit β1	Adaptin_N/COG5096
	DYNC1LI2	cyt. dynein 1 light intermediate chain 2	DLIC
	PAMCI	vesicles and endosomes localized/P-CIP1	RA (RalGDS/AF-6)
	RABGAP1	rab6 GAP/GAPCenA	PTB/Smc/TBC
	RUSC2	Iporin (interacts w/ rab1 & GOLGA2)	SH3/RUN
	PHLDB2	LL5β/vesicular/binds to γ-filamin & MT	PH_Gab/Smc

Gene names are listed according to the NCBI Refseq nomenclature. Protein domain names are as indicated in the NCBI CDD database.
